# Molecular origins of reduced activity and binding commitment of processive cellulases and associated carbohydrate-binding proteins to cellulose III

**DOI:** 10.1016/j.jbc.2021.100431

**Published:** 2021-02-19

**Authors:** Shishir P.S. Chundawat, Bhargava Nemmaru, Markus Hackl, Sonia K. Brady, Mark A. Hilton, Madeline M. Johnson, Sungrok Chang, Matthew J. Lang, Hyun Huh, Sang-Hyuk Lee, John M. Yarbrough, Cesar A. López, Sandrasegaram Gnanakaran

**Affiliations:** 1 Department of Chemical and Biochemical Engineering, Rutgers, The State University of New Jersey, Piscataway, New Jersey, USA; 2 Department of Chemical and Biomolecular Engineering, Vanderbilt University, Nashville, Tennessee, USA; 3 Department of Molecular Physiology and Biophysics, Vanderbilt University, Nashville, Tennessee, USA; 4 Department of Physics and Astronomy, Rutgers, The State University of New Jersey, Piscataway, New Jersey, USA; 5 Biosciences Center, National Renewable Energy Lab, Golden, Colorado, USA; 6 Theoretical Division, Los Alamos National Laboratory, Los Alamos, New Mexico, USA

**Keywords:** carbohydrate-binding proteins, cellulose, optical tweezers force spectroscopy, molecular dynamics, cellulases, carbohydrate-active enzymes, lignocellulosic biofuels, AFM, atomic force microscopy, CBM, carbohydrate-binding module, CD, catalytic domain, EA, extractive ammonia, FRAP, fluorescence recovery after photobleaching, MD, molecular dynamics, PMF, potential of mean force, XRD, X-ray diffraction

## Abstract

Efficient enzymatic saccharification of cellulosic biomass into fermentable sugars can enable production of bioproducts like ethanol. Native crystalline cellulose, or cellulose I, is inefficiently processed *via* enzymatic hydrolysis but can be converted into the structurally distinct cellulose III allomorph that is processed *via* cellulase cocktails derived from *Trichoderma reesei* up to 20-fold faster. However, characterization of individual cellulases from *T. reesei*, like the processive exocellulase Cel7A, shows reduced binding and activity at low enzyme loadings toward cellulose III. To clarify this discrepancy, we monitored the single-molecule initial binding commitment and subsequent processive motility of Cel7A enzymes and associated carbohydrate-binding modules (CBMs) on cellulose using optical tweezers force spectroscopy. We confirmed a 48% lower initial binding commitment and 32% slower processive motility of Cel7A on cellulose III, which we hypothesized derives from reduced binding affinity of the Cel7A binding domain CBM1. Classical CBM–cellulose pull-down assays, depending on the adsorption model fitted, predicted between 1.2- and 7-fold reduction in CBM1 binding affinity for cellulose III. Force spectroscopy measurements of CBM1–cellulose interactions, along with molecular dynamics simulations, indicated that previous interpretations of classical binding assay results using multisite adsorption models may have complicated analysis, and instead suggest simpler single-site models should be used. These findings were corroborated by binding analysis of other type-A CBMs (CBM2a, CBM3a, CBM5, CBM10, and CBM64) on both cellulose allomorphs. Finally, we discuss how complementary analytical tools are critical to gain insight into the complex mechanisms of insoluble polysaccharides hydrolysis by cellulolytic enzymes and associated carbohydrate-binding proteins.

Plant biomass, composed of polysaccharides like cellulose, is an ideal feedstock for bioconversion into various bioproducts like ethanol (1, 2). Cellulose is a β-(1→4)-glucose polymer that self-assembles to form crystalline fibrils that are recalcitrant to enzymatic depolymerization (3). Cellulolytic microbes (like *Trichoderma reesei* and *Clostridium thermocellum*) have therefore evolved with enzymes called cellulases that can deconstruct cellulose into fermentable sugars (4, 5, 6). Cellulases comprise two or more polypeptide domains called catalytic domains (CDs) and carbohydrate-binding modules (CBMs) (4). CBMs are characterized by a planar binding motif that is complementary to crystalline cellulose fibril structure to facilitate CD activity toward insoluble and structurally heterogenous cellulosic substrates (7). Although CBMs facilitate CD activity by proximity-based targeting effects, cellulolytic enzymes are inefficient for industrial applications often due to nonproductive interactions with the substrate that necessitate high protein loading requirements (4, 8).

Thermochemical pretreatment using acids, bases, or ionic liquids is therefore employed to increase polysaccharide accessibility to enzymes and reduce nonproductive cellulase binding (9, 10, 11). Pretreatment with anhydrous liquid ammonia results in conversion of native cellulose I to cellulose III allomorph (12), thereby improving hydrolytic activity of several fungal (13) and bacterial cellulase mixtures (14). However, processive exocellulases such as *Tr*Cel7A (or Cel7A from *T. reesei*) and *Tf*Cel6B (or Cel6B from *Thermobifida fusca*), which are workhorse cellulolytic enzymes, often show reduced activity on pretreated cellulose III for reasons poorly understood (14, 15). Although the processive mechanism of Cel7A on native cellulose I has been studied extensively using classical biochemical assays (16, 17, 18, 19) and molecular simulations (20, 21), there is limited consensus on how to monitor the initial enzyme association with the cellulose chain (18) or dissociation of nonproductively bound enzymes (16, 17) to identify rate-limiting steps impacting cellulose hydrolysis. Hence, there is a need for better experimental methods that can track cellulase binding and processive motility in real time with atomic-scale resolution for distinct substrates.

Single-molecule fluorescence imaging allows estimation of exocellulase binding kinetics parameters (*e.g.*, adsorption and desorption rates) (8, 22, 23), whereas high-speed atomic force microscopy allows tracking of motility of single cellulase molecules (24, 25). However, these methods cannot resolve the slower subnanometer translational rates of processive cellulases relevant to cellulose decrystallization and hydrolysis into cellobiose. We recently reported an optical tweezers force spectroscopy–based cellulase assay technique to track the single-molecule motility of Cel7A on native cellulose with subnanometer and millisecond resolution (26). Of interest, Cel7A CD in the absence of CBM1 showed lower dwell times between catalytic turnover steps suggesting that CBMs could impede full-length cellulase motility on native cellulose I owing to nonproductive binding. However, we lack a detailed understanding of the mechanistic role of CBMs in full-length processive cellulase binding and motility on cellulose I and other industrially relevant cellulosic substrates like cellulose III.

Here, we have applied our optical tweezer assay to investigate the initial binding stability of Cel7A and its processive motility on cellulose I and cellulose III. To understand the role of CBMs in our observed single-molecule binding instability of Cel7A toward cellulose III, we characterized the binding of CBM1 (from Cel7A) using classical “pull-down” binding assays and molecular dynamics simulations. We also developed a new optical tweezers–based CBM–cellulose bond “rupture” assay to characterize the binding behavior of single CBM1 proteins alone to distinct cellulose allomorph surfaces under applied force. To generalize these findings further, we characterized CBM3a (another type A CBM from *C. thermocellum*) using equilibrium pull-down and kinetic binding assays. We also characterized the binding partition coefficient of several other type A CBMs, belonging to Family 2a, 5, 10, and 64, to confirm that type A CBMs in general showed reduced binding toward cellulose III. Our results highlight some of the challenges associated with the use of overly simplistic Langmuir-type models to analyze classical protein–polysaccharide pull-down assay dataset. In summary, our work highlights how changes in CBM binding to distinct cellulose allomorphs can critically impact processive cellulase motility. Furthermore, our work highlights the necessity of using a multifaceted approach for characterizing the binding heterogeneity and multimodal nature of cellulase–cellulose interactions.

## Results

### *T. reesei* cellulase mixture shows improved activity toward cellulose III

**Figure 1:**
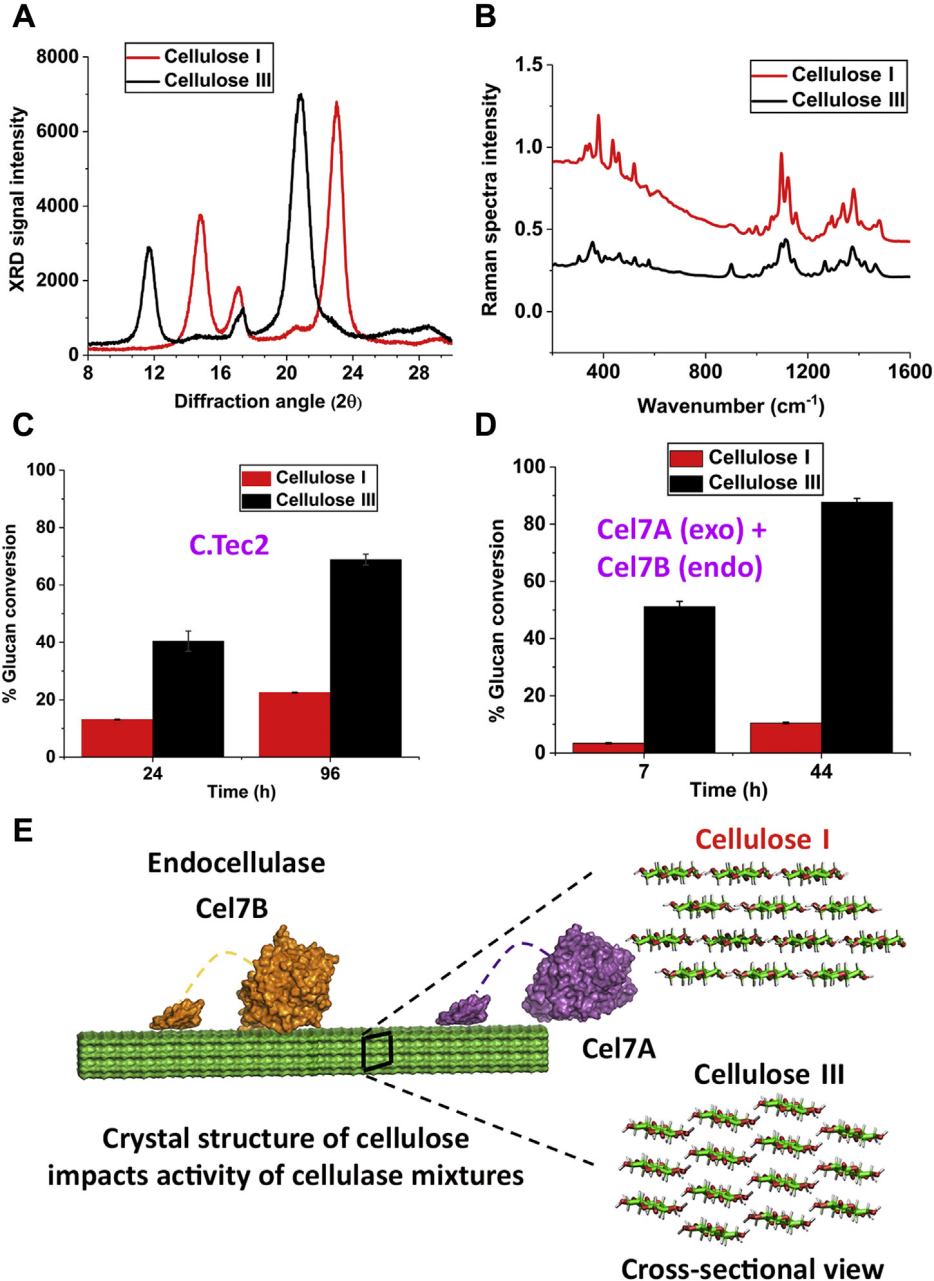
**Cladophora-derived highly crystalline cellulose III allomorph is more readily hydrolyzed by synergistic combinations of cellulases.***A,* XRD and (*B*) Fourier transform Raman spectra for cellulose I and cellulose III derived from Cladophora confirms respective allomorphic states. *C,* hydrolytic activity of Cellic C.Tec2 (Novozymes) cellulase cocktail toward cellulose I and cellulose III for varying hydrolysis times. *D,* hydrolytic activity of an equimolar mixture of *T. reesei* Cel7A exo- and Cel7B endo-cellulases, respectively, supplemented with 10% β-glucosidase, toward cellulose I and III. Specific activity for Cel7A alone can be found in Table S1.*E*, cross-sectional view of model cellulose I and III allomorphs depicting key morphological differences in fibril shape that impact endo–exo cellulase synergism toward cellulose III, as also reported previously (15). Published crystal structures of Cel7A (PDB code: 1CEL) and Cel7B (PDB code: 1EG1) were used to generate this figure. Here, hydrolytic activity is reported as mean value for replicate assays with error bars depicting one standard deviation.

*Cladophora* sp. (*Cladophora glomerata*)–derived highly crystalline cellulose I fibers were isolated, as described previously (26), followed by anhydrous liquid ammonia pretreatment to prepare cellulose III (27). Details about cellulose isolation, ammonia pretreatment, spectroscopic characterization, and enzymatic hydrolysis methods are provided in the SI Appendix Experimental Procedures section. Spectroscopic characterization using X-ray diffraction (XRD) and Fourier transform Raman spectroscopy were conducted to confirm the conversion of cellulose I to cellulose III allomorph following ammonia pretreatment and also measure substrate characteristics like cellulose crystallinity index (CrI) and crystallite size. Similar to previous work (27, 28, 29, 30), XRD equatorial reflections for (100), (010), and (110) crystallographic planes for native Cladophora cellulose I were at approximately 14.9°, 17.1°, and 23.0° Bragg angles (2ϴ), respectively (see Fig. 1*A*). As previously described (27, 31), equatorial reflections for (010), (002), and (100) crystallographic planes for Cladophora cellulose III were at approximately 11.8°, 17.4°, and 20.9° Bragg angles (2ϴ), respectively. Based on the Segal method, cellulose crystallinity index was estimated to be about 90% to 95% for both allomorphs. Cellulose crystallite size was about 8.5 to 9 nm for both allomorphs, estimated using the Scherrer equation based on the full width at half maximum of the equatorial plane reflection peak. See Figure S1 for an atomic force microscopy (AFM)-based analysis of individual crystallite fibers, which also agrees with the XRD results and expected crystallite shape as reported in previous AFM-based analysis of Cladophora-derived cellulose (23, 25). Cladophora cellulose-based crystallites were at least two to three times larger in cross-sectional diameter than previously reported for cellulose microfibrils derived from higher-order plants, such as cotton linters (15). Similar to previous reports (15, 32, 33), Raman spectroscopy also independently confirmed that native Cladophora cellulose I was completely converted into cellulose III following ammonia treatment (Fig. 1*B*).

Next, we performed enzymatic hydrolysis assays to test the activity of a *T. reesei*-based commercial cellulase enzyme mixture (*i.e.*, Cellic C.Tec2) toward cellulose I and cellulose III allomorphs. The commercial cellulase enzyme mixture showed ∼3-fold improved activity toward cellulose III *versus* cellulose I at the 24 and 96 h saccharification time points (see Fig. 1*C*). We also confirmed that a purified mixture of *T. reesei* endo- and exocellulases Cel7B and Cel7A, respectively, show up to 10- to 20-fold improved activity toward cellulose III (see Fig. 1*D*). These results support our previous observations that improved activity of cellulase mixtures toward cellulose III arises owing to improved endo–exo synergistic activity (14). Cellulose III has a slightly stepped or “jagged” surface due to the underlying modification of the crystal structure caused by *trans*-*gauche* to predominantly *gauche*-*trans* rotameric state of the C6-hydroxymethyl groups (see Fig. 1*E*). This jagged cellulose III surface has been shown previously to be more readily hydrated by water molecules, unlike cellulose I (13, 15), and was therefore hypothesized to impact cellulolytic enzyme binding and/or activity. Here, we also characterized the specific activity of purified Cel7A alone toward cellulose I and cellulose III at various enzyme loadings of 0.5, 2.5, and 10 mg/g (see Table S1). These results show up to 3-fold improved enzyme activity toward cellulose III at higher enzyme loadings, such as 2.5 and 10 mg/g; however, that difference becomes nearly indistinguishable at the lowest enzyme loading of 0.5 mg/g, similar to activity trends previously observed by Gao *et al*. (13) and Shibafuji *et al*. (22). The underlying molecular origins for decrease in processive bulk activity of Cel7A toward cellulose III at very low enzyme loadings is not clear currently. Previous single-molecule Cel7A motility assays have been conducted at high enzyme loadings where Cel7A “traffic jams” and poorly understood protein–protein interactions seem to play an important role in cellulose hydrolysis by cellulases (22). However, the activity of Cel7A on cellulose III in the absence of such surface crowding effects at the single-enzyme level has not been characterized using high-resolution optical tweezer–based tracking methods.

### Single-molecule Cel7A binding and initial substrate engagement is impaired on cellulose III

**Figure 2:**
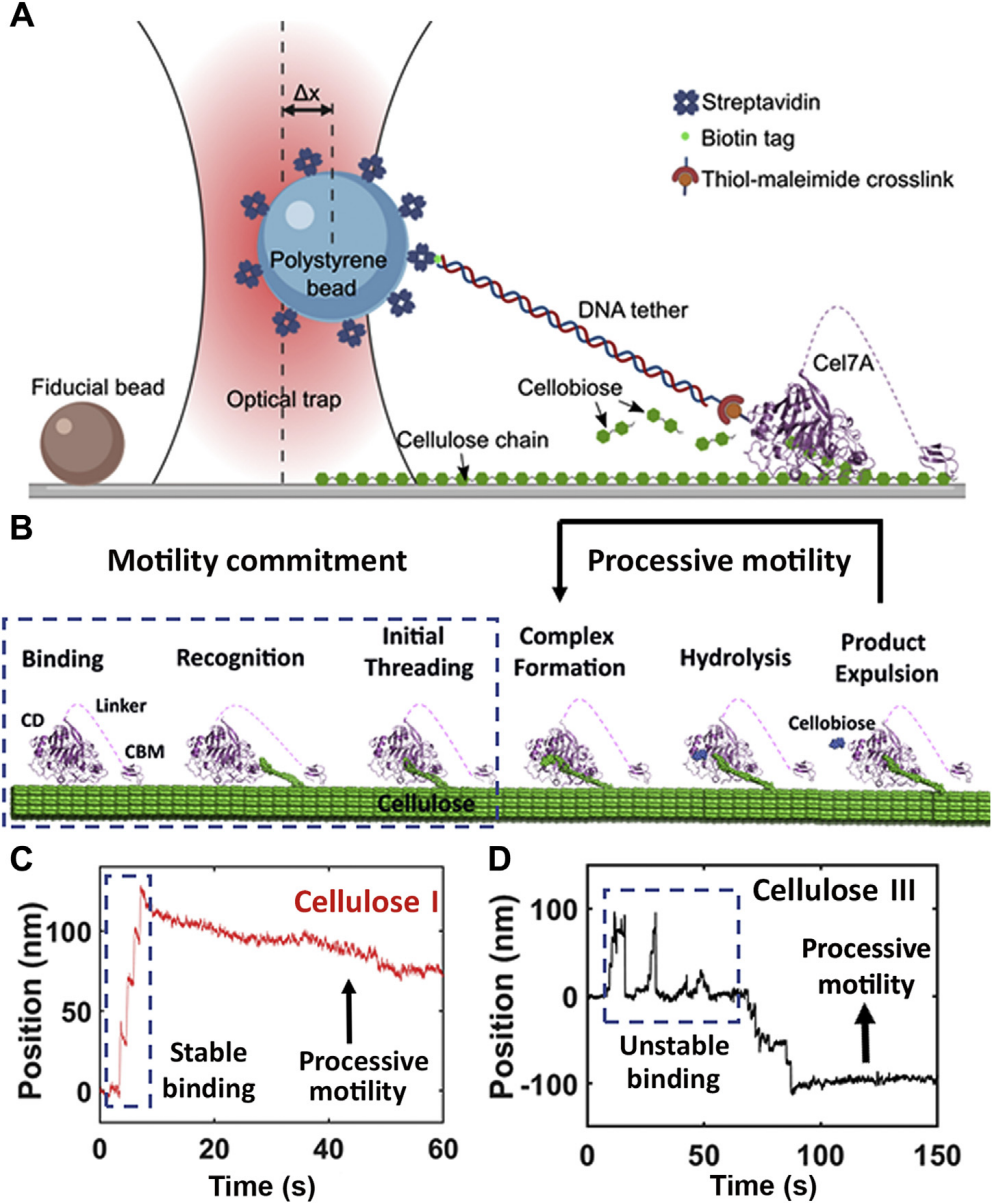
**Processive cellulase Cel7A shows unstable single-molecule binding on cellulose III.***A*, schematic of cellulase motility assay setup (not to scale) is shown where a streptavidin-coated bead is tethered to a single Cel7A molecule *via* a thiol-maleimide cross-link to a DNA linker containing a biotin tag on the opposite end and bound to cellulose to initiate Cel7A motility to produce cellobiose. Here, Δx represents the bead displacement from the trap center. Figure created with BioRender.com. *B*, processive cellulase Cel7A degrades cellulose *via* a multistep mechanism involving (i) enzyme binding to cellulose, (ii) recognition of cellulose reducing end by catalytic domain (CD), (iii) threading of cellulose chain through active site, (iv) formation of a catalytically active complex by nucleophilic attack, (v) glycosidic bond hydrolysis, and (vi) cellobiose product expulsion from active site and forward stepping of the enzyme. Steps (iv), (v), and (vi) are repeated multiple times, leading to processive motion until the enzyme desorbs from the surface. Steps (i), (ii), and (iii) precede the processive motion of enzyme and hence determine enzyme commitment to motility (collectively called here as “motility commitment”). *C*, single bead position trace representing initial stable binding to Cellulose I followed by Cel7A motility. *D*, initial unstable binding to cellulose III followed by eventual Cel7A motility. The position of the bead fluctuates significantly in the case of cellulose III to about 100 nm, indicating that the enzyme desorbs from the cellulose surface multiple times before initiating processive motion. Additional representative traces showcasing unstable protein binding prior to Cel7A motility initiation can be found in Figure S2. Published crystal structure of Cel7A (Protein Data Bank code: 1CEL) and Cel7B (Protein Data Bank code: 1EG1) were used to generate this figure. CBM, carbohydrate-binding module.

Single-molecule cellulase motility assays were performed on both cellulose allomorphs to study how subtle differences in cellulose crystal structure impact the binding and processive motility of Cel7A. Details regarding Cel7A motility assay and data analysis rationale are published elsewhere (26). Briefly, Cel7A was attached *via* sulfo-SMCC (*i.e.*, sulfosuccinimidyl-4-(N-maleimidomethyl)cyclohexane-1-carboxylate) cross-linking to a thiol tag on the end of a biotinylated 1010-bp DNA tether and attached to a 1.25 μm streptavidin-coated polystyrene bead (see Fig. 2*A*). The Cel7A functionalized bead was positioned directly above a cellulose fiber to initiate binding, and the bead position was monitored as the enzyme first bound, hydrolyzed, and processed along the cellulose surface for cellulose I or cellulose III fibril surface. Based on the mechanism for Cel7A action on cellulose (see Fig. 2*B*), we propose the term “motility commitment” or “binding commitment” to describe the steps prior to initiation of processive motility, *i.e.*, binding, recognition, and initial cellulose chain threading within the Cel7A active-site tunnel. During our motility assays, it was possible for us to observe the initial motility commitment of Cel7A for distinct cellulose allomorph surfaces immediately prior to processive motility initiation. To initiate the single-molecule Cel7A motility, a functionalized bead is positioned directly above a surface-affixed cellulose fiber and periodically gently pulled *via* the piezo stage to test for bound enzymes. Such initial binding is considered stable or committed when the Cel7A–cellulose bond survives, and the enzyme exhibits motility for a period greater than 10 s. Representative traces of binding stability/instability for Cel7A binding to cellulose I and cellulose III are shown in Figure 2, *C* and *D*, respectively (see Fig. S2 for additional representative traces). In some cases, the full-length Cel7A was seen to bind but not commit to significant motility on the cellulose surface highlighting nonproductively engaged cellulases. Alternatively, Cel7A–cellulose bond instability is revealed through initial bead displacement followed by rapid detachment. Given these criteria and observation times of 600 s for each trace, Cel7A–cellulose initial bond instability was determined to be significantly lower for cellulose I (12% of all traces, N = 17) than cellulose III (23% of all traces N = 13). Although this rapid bead detachment as shown in Figure 2*D* could have been driven in principle either due to improper CBM and/or by CD binding/engagement, the large 100-nm spikes in the highlighted region (labeled “unstable binding”) led us to hypothesize that the CBM likely plays a prominent role in this phenomenon owing to its primary function of increasing proximity of CD near cellulose surface (to within a few nanometers). Furthermore, we analyzed the subsequent processive motility cycles of Cel7A by extracting the enzymes step sizes and dwell time distributions as further discussed below.

### Cel7A shows marginally reduced hydrolytic velocity and longer dwell times between catalytic cycles on cellulose III

**Figure 3:**
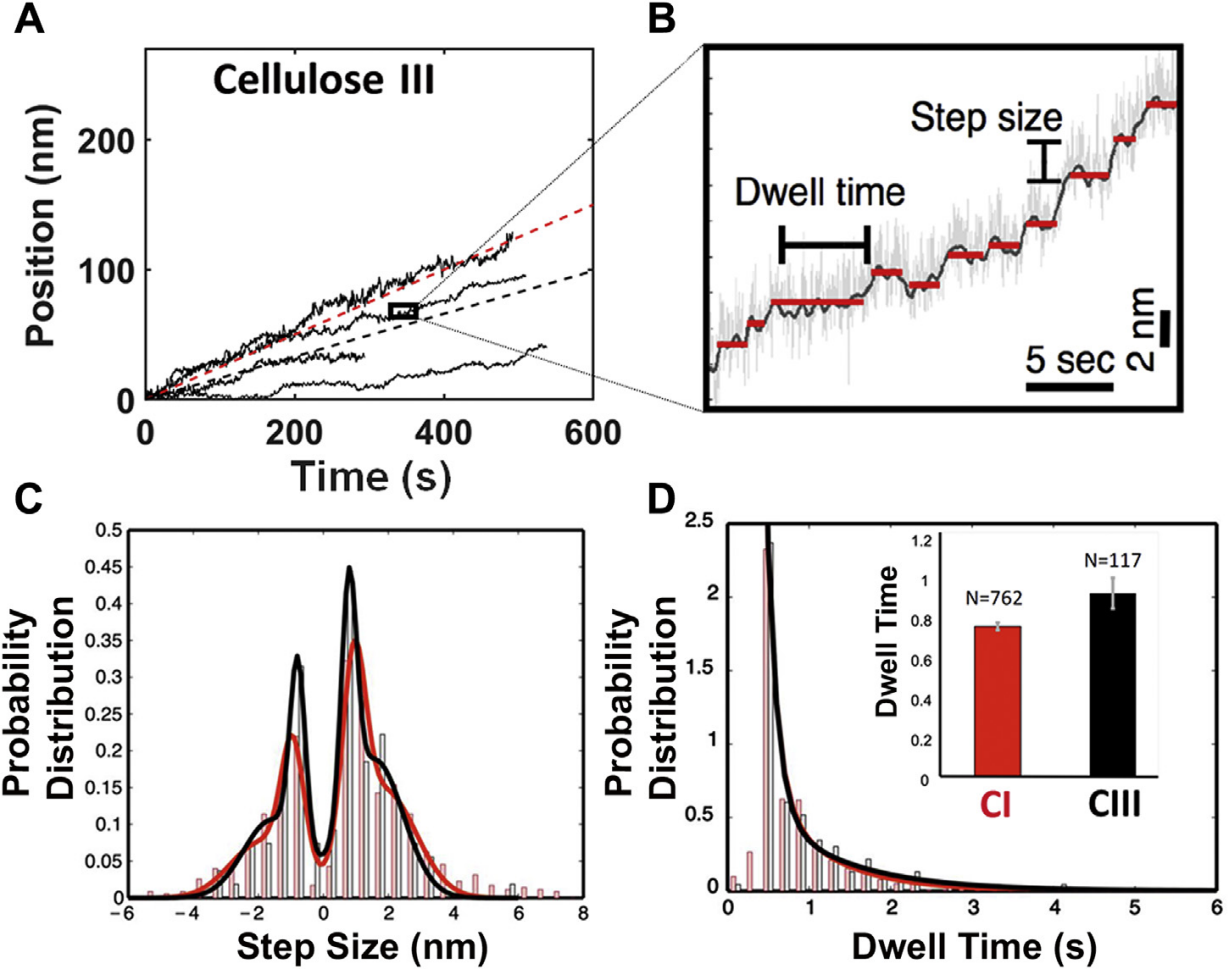
**Processive cellulase Cel7A shows reduced single-molecule processive velocity on cellulose III.***A*, representative traces of Cel7A enzyme motility on Cladophora-derived cellulose III (in *black*) are shown here. *Dashed lines* in (*A*) indicate average velocities, 0.25 ± 0.35 nm s^−1^ (SD; cellulose I; N = 68; in *red*) and 0.17 ± 0.14 nm s^−1^ (SD; cellulose III; N = 30; in *black*). Representative traces for cellulose I can be found in Figure S3. *B*, magnified view of individual motility cycle of enzyme that is made up of several dwells and steps. Dwell time and step size distributions are obtained as previously discussed by Brady *et al*. (26). *C*, all individual motility traces were analyzed to determine step-size distributions (as *bars*) fitted to Gaussian curves based on the fundamental (∼1 nm) and 2× fundamental steps expected for Cel7A cellodextrin products (*i.e.*, cellobiose) profile on cellulose I (in *red*) and III (in *black*). Slightly increased back stepping of Cel7A on cellulose III (39% reverse steps) *versus* cellulose I (35% reverse steps) is seen here. *D*, dwell time distributions (as *bars*) were fitted to single-exponential decay curves to estimate the characteristic dwell time constant (see *inset*) and were found to be higher for cellulose III (in *black*) *versus* cellulose I (in *red*). The average dwell times for cellulose I and cellulose III are 0.75 and 0.92 s, respectively.

Representative individual Cel7A processive motility traces and average enzyme velocity on cellulose I and cellulose III are shown in Figures S3 and 3*A* respectively, which capture the processive motion of single enzymes on the cellulose surface during its deconstruction into soluble sugars (namely cellobiose). The average Cel7A velocity on cellulose I was 0.25 ± 0.35 nm s^−1^ (SD; N = 68 motility traces), which is marginally higher than that seen on cellulose III, 0.17 ± 0.14 nm s^−1^ (SD; N = 30 motility traces). The dwell time and step size distributions were then extracted for each individual motility trace as described previously (26) and highlighted in Figure 3*B*. Extraction of the step size distributions from individual motility traces (see Fig. 3*C* for step size distributions on cellulose I [red] and cellulose III [black] overlaid) indicated that the mean step size for both cellulose I and cellulose III is close to the 1 nm length of the expected cellobiose product. However, the dwell time for cellulose III was 0.92 s as compared with 0.75 s for cellulose I (Fig. 3*D*). The increased dwell time, frequent reverse stepping or back motility, and marginally reduced forward enzyme velocity observed on cellulose III *versus* cellulose I partially explain the slightly lowered or comparable Cel7A bulk saccharification activity observed previously toward cellulose III at very low enzyme loadings (15). In summary, Cel7A shows impaired motility commitment (or initial binding) and slightly reduced processive motility (or hydrolytic velocity) on cellulose III. We hypothesize that processive cellulases like Cel7A show reduced binding/activity toward cellulose III likely due to impaired motility commitment driven by unstable binding to the cellulose surface. As shown in Figure 2*B*, the first step of motility commitment involving enzyme binding to cellulose is primarily driven by the CBM (34). Although the CD is responsible for processive motility, the CBM likely also plays a critical role by stepping in tandem with the CD (35). Hence, the rest of this study was aimed toward better understanding the role of CBMs in anomalous motility commitment and processive motility behavior on cellulose III, using a complementary suite of experimental and computational methods.

### CBM1 isolated from Cel7A displays lower binding affinity toward cellulose III

**Figure 4:**
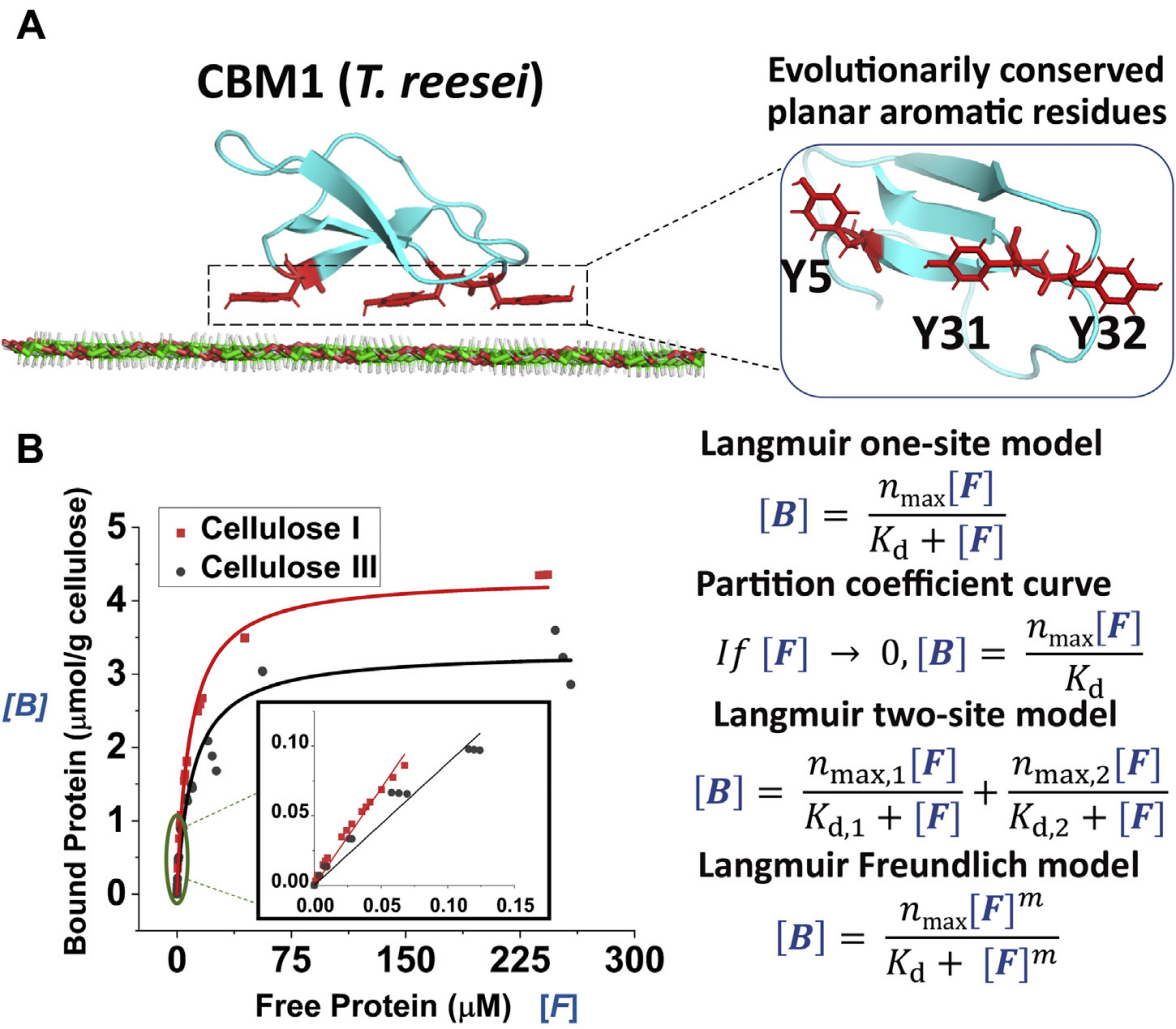
**CBM1–cellulose solid-depletion binding assay data and fitted Langmuir-type model equations.***A*, CBM1 (Protein Data Bank code: 1CBH) from Cel7A docked on the hydrophobic face of crystalline cellulose I with axial hydrogens is shown in *white outlines* (*left*). The planar binding motif comprising aromatic residues highlighted in *red* (Y5, Y31, and Y32) is shown in the *inset* (*right*). *B*, GFP-CBM1 (*T. reesei*) equilibrium binding data for Cladophora cellulose I and cellulose III to estimate equilibrium adsorption constants are shown here. Nonlinear relationship between bound and free GFP-CBM1 concentration for cellulose I (in *red dots*) and cellulose III (in *black dots*) is shown here for replicate assays. *Fitted line* depicts a Langmuir one-site model. *Inset graph* shows the linear region of this model to estimate partition coefficient. Relationship between bound and free protein for various adsorption models tested such as Langmuir one-site, two-site, and Freundlich models is shown here. Representative model fits for original CBM1–cellulose binding data are shown in Figure S4, with results summarized in Table 1.

Cel7A possesses a CBM from family 1 (called CBM1 hereon), whose structure–function relationships have been well characterized (36, 37, 38). However, CBM1 binding toward nonnative allomorphs such as cellulose III has not been studied in detail. CBM1 orients and binds to crystalline cellulose I through strong hydrophobic stacking interactions between conserved planar aromatic residues (Y5, Y31, Y32) and axially oriented hydrogen moieties of individual glucosyl units of the cellulose polymer chain (39), as illustrated in Figure 4*A*. Here, we characterized the equilibrium binding interactions of CBM1 toward cellulose I and cellulose III using solid-state depletion or classical protein–polysaccharide pull-down binding assays (40). CBM1 was tagged with green fluorescent protein (GFP) to allow protein quantitation based on fluorescence as described previously (41). Details regarding gene sequences, cloning, expression, and protein purification strategies for all CBMs tested in this study can be found in the SI Appendix Experimental Procedures section (41).

Classical pull-down binding assays employing an extensive range of protein concentrations (0–250 μM) resulted in protein–polysaccharide adsorption dataset for CBM1 as shown in Figure 4*B*. Langmuir one-site/two-site and Langmuir-Freundlich–based adsorption models (equations displayed in Fig. 4*B*) were fitted to the adsorption dataset using nonlinear regression, as described previously (7, 13, 40). The model-fitting outputs for all models tested here are shown in Figure S4. This analysis allowed estimation of the maximum available binding sites (*n*_max_) and equilibrium dissociation constant (*K*_d_), in addition to other model-specific parameters (Table 1). The total number of binding sites for cellulose I was always higher (∼1.2–1.5 fold) compared with cellulose III in all cases, except in the case of high-affinity binding sites (*n*_max_) for the two-site model. There was ∼1.2 to 7-fold reduction in binding affinity (*i.e.*, inverse of dissociation constant *K*_d_) for cellulose III depending on the exact fitted model. Our analysis indicates that the exact fold reduction in CBM binding affinity for cellulose III is highly dependent on the model used and highlights a potential limitation of classical binding assay methods. To further highlight limitations of the classical assay methods, we also performed data truncation analysis by trimming down our binding dataset for CBM1 to exclude higher protein concentrations (*i.e.*, included maximum concentrations up to 15 or 50 μM only) (see Table 2). We observed that the number of predicted binding sites for both cellulose I and cellulose III decreased by ∼1.3- to 1.8-fold for the truncated datasets. Of interest, our truncated dataset fitted models predicted a slightly weaker affinity of CBM1 toward cellulose I *versus* cellulose III, which was contrary to predictions made from model fitting to the full dataset. Hence, to resolve this apparent uncertainty in relative binding affinity trends due to limitations of classical binding assay methods, we resorted to potential of mean force (PMF) calculations to also theoretically estimate the CBM1–cellulose binding affinity using a first-principles approach.

**Table 1:** Langmuir binding model parameters for GFP-CBM1 adsorption to Cladophora-based cellulose I and III

	Cellulose I	Cellulose III
Langmuir one-site binding model		
*n*_max_	4.34 ± 0.05	3.32 ± 0.09
*K*_d_	8.69 ± 0.31	10.55 ± 0.91
RMSE	0.17	0.17
Langmuir two-site binding model		
*n*_max,1_	4.14 ± 0.00	2.81 ± 0.03
*K*_d,1_	13.68 ± 0.13	25.06 ± 0.95
*n*_max,2_	0.42 ± 0.01	0.75 ± 0.03
*K*_d,2_	0.13 ± 0.00	0.92 ± 0.07
RMSE	0.11	0.12
Langmuir–Freundlich binding model		
*n*_max_	4.80 ± 0.02	3.82 ± 0.02
*K*_d_	6.90 ± 0.04	7.77 ± 0.07
*m*	0.77 ± 0.00	0.73 ± 0.00
RMSE	0.14	0.14

RMSE, root mean square error.

Here, binding dissociation constant (*K*_d_; μM), maximum available binding sites (*n*_max_; μmol/g cellulose), and Freundlich power constant (*m*) fitted parameters are shown. Model fitting details for all Langmuir-based adsorption models are provided in the SI Appendix. The errors reported were standard errors to parameter fits obtained. Representative model fits for original CBM1–cellulose binding data are shown in Figure S4.

**Table 2:** Langmuir one-site binding model fitting analysis to truncated CBM1–cellulose I pull-down binding assay data set

	Cellulose I	Cellulose III
Fitted parameters for original data set (without truncation)		
*n*_max_	4.34 ± 0.05	3.32 ± 0.09
*K*_d_	8.69 ± 0.31	10.55 ± 0.91
RMSE	0.17	0.17
Fitted parameters for dataset truncated to 50 μM		
*n*_max_	3.95 ± 0.05	3.23 ± 0.14
*K*_d_	7.05 ± 0.24	9.88 ± 1.07
RMSE	0.008	0.16
Fitted parameters for dataset truncated to 15 μM		
*n*_max_	3.18 ± 0.07	1.82 ± 0.05
*K*_d_	4.68 ± 0.21	2.91 ± 0.21
RMSE	0.008	0.05

Dataset for CBM1 binding to Cladophora-based cellulose I was truncated to maximum 50 and 15 μM free protein concentration and fitted again using a Langmuir one-site binding model. Here, the model parameters binding dissociation constant (*K*_d_; μM) and maximum available binding sites (*n*_max_; μmol/g cellulose) are reported in addition to the root mean square error (RMSE) for model fitting. Standard error from the mean for each parameter is reported here.

### Molecular simulations predict lower CBM1 binding free energy toward cellulose III

**Figure 5:**
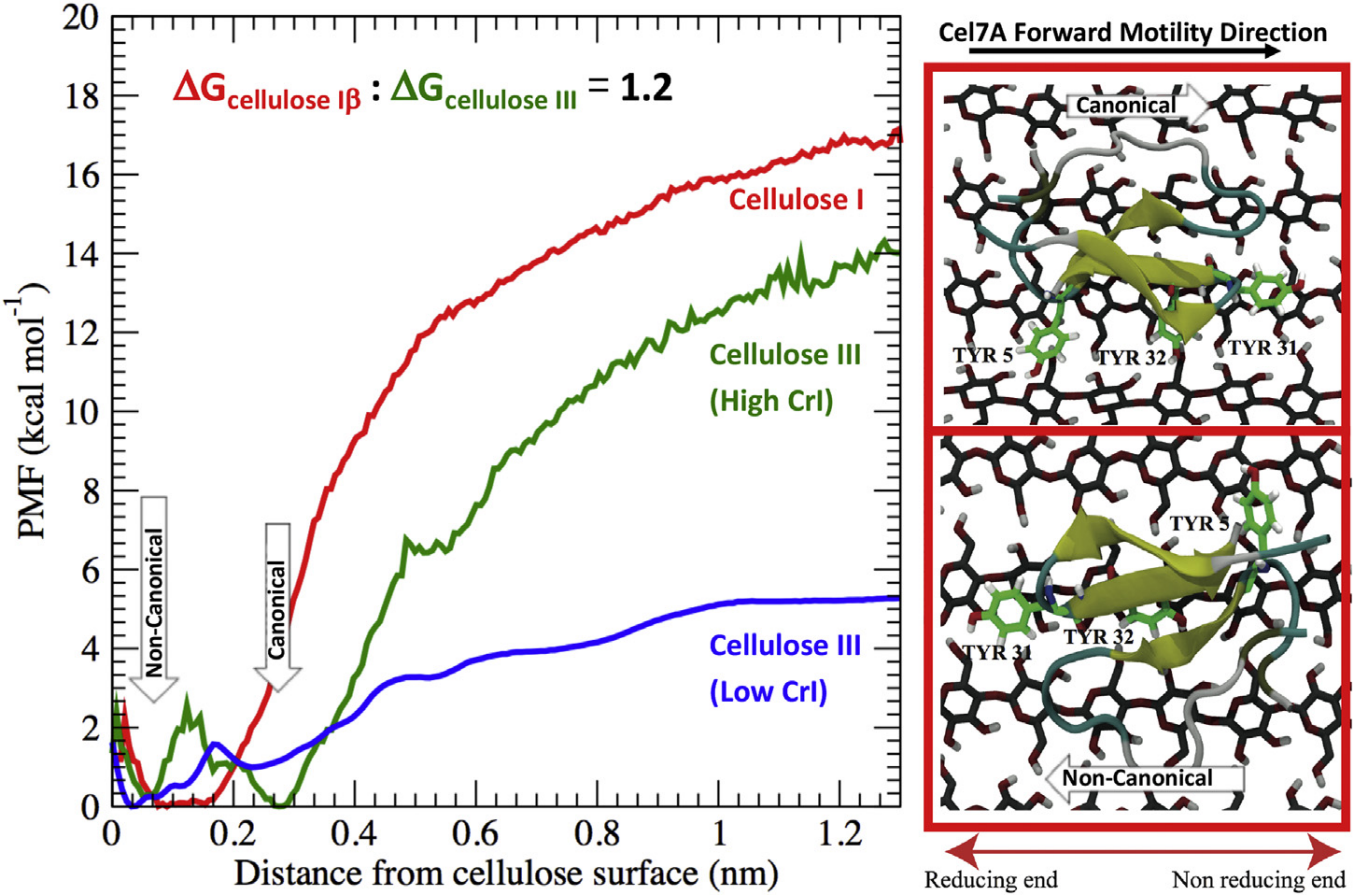
**Molecular dynamics simulations provide an atomistic basis for reduced affinity and distinct multimodal binding interactions of CBM1 to cellulose III allomorph surfaces.** Potential mean force (PMF) calculations were carried out to estimate the binding free energy of CBM1 with cellulose allomorphs to show that binding free energy is at least 1.2-fold higher for cellulose I (in *red*) *versus* high crystallinity cellulose III. High crystallinity index (CrI; in *green*) and low CrI (in *blue*) models of cellulose III were both studied here for sake of comparison (see SI Appendix for details). Note that the two energy wells for cellulose III correspond to the canonical and noncanonical orientations observed for bound CBM1. Canonical orientation refers to Y5 residue facing the reducing end, as it favors the processive motility of Cel7A from reducing to nonreducing end of cellulose chain. Figure *inset* here shows canonical (*top*) and noncanonical (*bottom*) orientations of CBM1 on high CrI cellulose III, along with the preferred direction of the processive Cel7A motility during cellulose saccharification. Additional details about the molecular dynamics analysis and root mean square fluctuations of critical binding motif aromatic residues due to improper CBM1 stacking to cellulose III surface are highlighted in Figure S5.

Unbiased MD simulations were first performed to obtain the preferred binding orientation of CBM1 on model cellulose I and III crystal surfaces (see Fig. S5). As shown in Figure S5, *C* and *D*, the CBM1 planar binding surface aromatic residues exhibit greater root mean square fluctuation on cellulose III, indicating improper stacking of aromatic residues specifically the Y5 residue. The results from unbiased MD simulations are discussed in detail in the SI Appendix Results and Discussion section. A PMF was then calculated to estimate the CBM1 binding free energy during adsorption to the hydrophobic surface of both cellulose allomorph models. As shown in Figure 5, in the case of cellulose I, only one PMF energy minimum well was observed corresponding to the dominant CBM1–cellulose configuration observed during the unbiased MD simulations whereby the Y31 residue faces the nonreducing end (*i.e.*, the expected canonical orientation based on native Cel7A favored activity from the nonreducing end of cellulose). However, in the case of highly crystalline cellulose III, two PMF energy minima wells were observed, one in which Y31 faces the reducing end closer to the surface and another in which it faces the nonreducing end further away from the surface. These configurations are annotated as noncanonical and canonical, respectively, in Figure 5. These two configurations are separated by roughly 0.2 nm in the PMF free energy diagram, where the distance is measured normal to the cellulose surface, with a marginal energetic barrier of 2 kcal/mol separating the two minima wells. A closer examination of the CBM1 structure revealed that if the protein binds in the so-called canonical orientation to the cellulose III surface at a shorter distance, then the Y5 residue exhibits significant steric clashes with the cellulose III adjacent surface chains (also shown in Fig. S5*D*). The impact of such steric clashes is also captured in the higher root mean square fluctuation values observed for the key binding motif aromatic residues when CBM1 is weakly bound to cellulose III. This explains why the “canonical” CBM1 configuration is observed only at slightly longer distances away from the cellulose III surface. Irrespective of the preferred orientation for CBM1 to cellulose III surface and the degree of model cellulose III crystallinity, the calculated free energy of binding for CBM1 was always lower for cellulose III compared with cellulose I. These results support predictions from certain Langmuir adsorption models where the estimated equilibrium binding affinity for CBM1 was lower for cellulose III than for cellulose I.

### Family 3a CBM also shows reduction in binding to cellulose III *via* both equilibrium and kinetic binding assays

To generalize our findings regarding reduced CBM binding affinity toward cellulose III beyond CBM1, we also characterized the equilibrium and kinetic binding behavior of another well-studied type A CBM from family 3a (also called CBM3a) from *C. thermocellum* (42, 43). Classical binding assays and adsorption model fitting analysis were performed in a similar way to CBM1 (see Table 3 for binding parameters and Fig. S6 for model fitting outputs). Reduced binding affinity (∼2- to 14-fold higher *K*_d_) was observed for cellulose III depending on the exact model used for data fitting. The total number of binding sites predicted for cellulose I was slightly lower than for cellulose III except when the dataset was fitted using a one-site model or in the case of high-affinity sites in the two-site model. A closer inspection of the binding assay dataset (Fig. S6) suggests that even at the highest CBM3a concentrations tested (∼50 μM), proper saturation behavior was not fully observed, which might lead to spurious binding parameters as previously discussed during truncation analysis of CBM1 binding data. An alternative approach is to characterize the partition coefficient, which is the linear slope of binding isotherm at lower protein loadings (as shown in inset of Fig. 4*B*). Here, we also characterized the partition coefficient of a larger library of type A CBMs (including CBM1 and CBM3a) and observed a clear reduction in binding toward cellulose III in all cases (see Fig. S7 for raw data and Fig. S8 for partition coefficient bar graph). The partition coefficient is the slope of the initial linear region of the binding curve between bound protein (μmol/g cellulose) and free protein (μΜ) as shown in Figure 4*B*.

**Table 3:** Langmuir binding model parameters for GFP-CBM3a adsorption to Cladophora-based cellulose I and III

	Cellulose I	Cellulose III
Langmuir one-site binding model		
*n*_max_	2.36 ± 0.10	1.48 ± 0.11
*K*_d_	2.15 ± 0.46	6.15 ± 1.75
RMSE	0.22	0.17
Langmuir two-site binding model		
*n*_max,1_	1.81 ± 0.03	3.66 ± 0.22
*K*_d,1_	16.64 ± 1.35	227.30 ± 17.8
*n*_max,2_	0.99 ± 0.03	0.67 ± 0.02
*K*_d,2_	0.28 ± 0.02	0.64 ± 0.05
RMSE	0.17	0.12
Langmuir–Freundlich binding model		
*n*_max_	3.21 ± 0.05	7.58 ± 0.50
*K*_d_	2.61 ± 0.06	19.10 ± 1.27
*m*	0.53 ± 0.01	0.42 ± 0.01
RMSE	0.17	0.13

RMSE, root mean square error.

Here, binding dissociation constant (*K*_d_; μM), maximum available binding sites (*n*_max_; μmol/g cellulose), and Freundlich power constant (*m*) fitted parameters are shown. Model fitting details for all Langmuir-based adsorption models are provided in the SI Appendix. The errors reported were standard errors to parameter fits obtained. Representative model fits for original CBM3a–cellulose binding data are shown in Figure S6.

**Figure 6:**
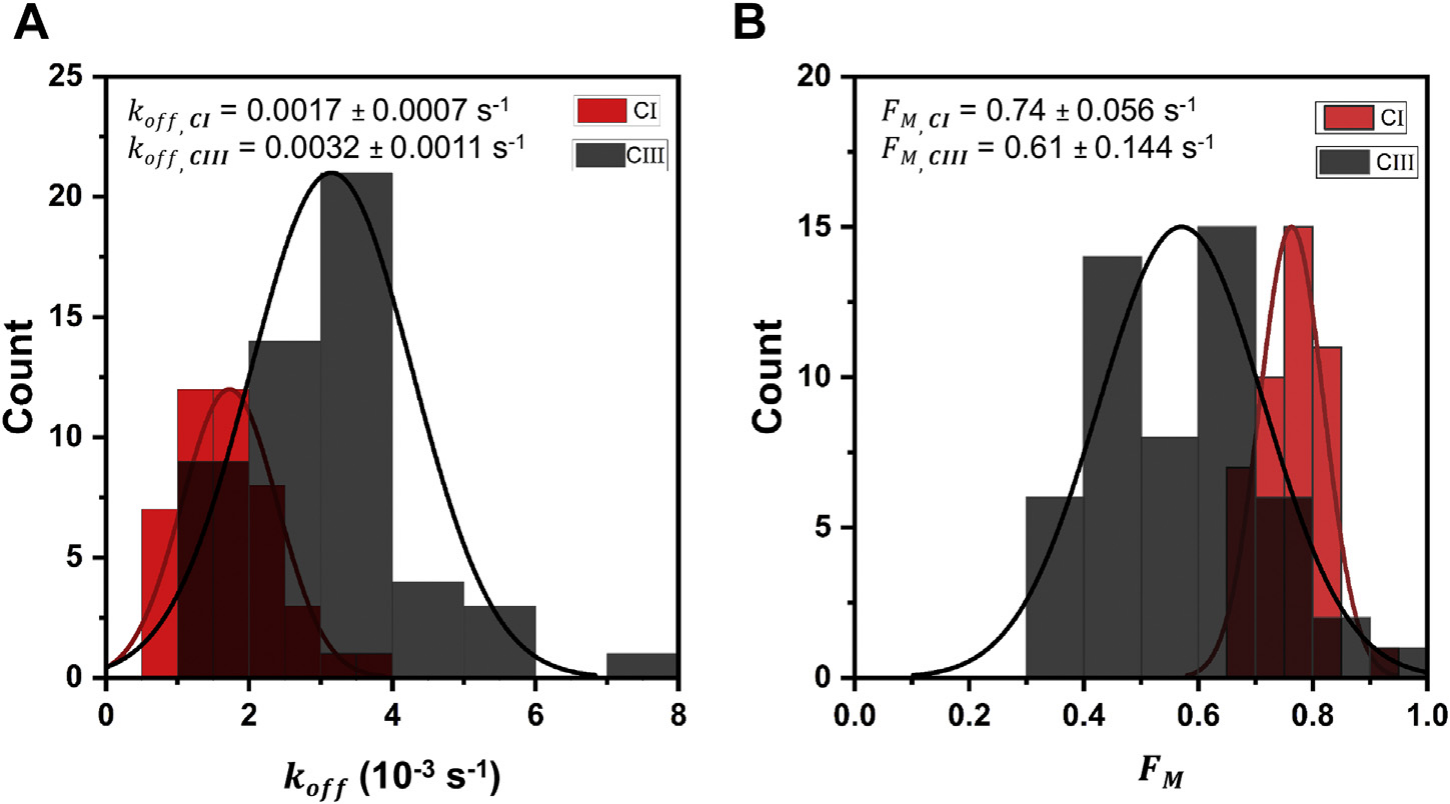
**Fluorescence recovery after photobleaching (FRAP)-based analysis of GFP-CBM3a binding kinetics to cellulose allomorphs indicates an increase in the dissociation off-rate (*k***_**off**_**) for CBM3a for cellulose III *versus* cellulose I.***A* and *B*, compare the dissociation off-rate and mobile fraction of reversibly bound proteins (or *F*_*M*_) histograms for GFP-CBM3a on Cladophora cellulose I (CI) *versus* cellulose III (CIII) with the Gaussian fit parameters (mean ± SD) as *insets*. Here, $F_{M}$ represents the fraction of reversibly bound GFP-CBM3a and based on the model used to analyze the FRAP data are dependent on the pseudoadsorption rate, desorption off-rate, concentration of protein in solution, and slope of the calibration curve between fluorescence intensity and protein concentration. Details about the FRAP model parameters and data analysis approach is provided in the SI Appendix Methods. Representative FRAP recovery curves are shown in Figure S9.

We further characterized the binding kinetics of CBM3a using fluorescence recovery after photobleaching (FRAP) and quartz crystal microbalance with dissipation (QCM-D). The raw data from FRAP and QCM-D assays are summarized in Figures S9 and S10, respectively. Briefly, similar to previous work (7, 44), GFP-CBM3a binding kinetic parameters to cellulose allomorphs were obtained by fitting the FRAP curves to a binding-dominated model ignoring any diffusion relevant contributions. Our FRAP analysis revealed that CBM3a gave a 1.9-fold increase in the desorption rate constant (*k*_off_) for Cladophora derived cellulose III compared with cellulose I (Fig. 6*A*). Similarly, QCM-D also showed an ∼3-fold increase in *k*_off_ for Avicel derived cellulose III nanocrystals (Table 4). A detailed discussion of FRAP and QCM-D results can be found in the SI Appendix Results and Discussion section. We were also able to fit another parameter *F*_M_, which represents the fraction of reversibly bound GFP-CBM3a and was used along with the desorption rate constant to draw conclusions about the relative change in adsorption rate constants as discussed in the SI Appendix (Fig. 6*B*). A key limitation of these assays is the inability to estimate the true adsorption rate constant (*k*_on_); however, the desorption rate constant showed a clear increase for cellulose III, corroborating the reduction in binding affinity as indicated by the solid-depletion assays. Overall, these results indicate that type A CBMs like CBM1 and CBM3a show reduced binding affinity toward cellulose III, potentially leading to impaired motility commitment of tethered processive cellulases. Since classical binding assay methods cannot resolve the various binding modes of CBM–cellulose interactions and how these modes differ in the case of cellulose III *versus* cellulose I, we developed a single-molecule CBM–cellulose bond rupture assay. In addition, results from this single-molecule assay can shed light on the suitability of using multisite models for analyzing classical pull-down binding assays.

**Table 4:** Kinetic rate constants for GFP-CBM3a adsorption and desorption toward nanocrystalline cellulose allomorphs estimated using Quartz Crystal Microbalance (QCM-D)-based binding assay data

Cellulose allomorph	*A* (×10^−12^ bound molecules)	$k_{\mathrm{on}}^{*}$(**s**^**−1**^)	*k*_off_ (×10^−3^ s^−1^)
Cellulose I	145.55 ± 0.40	0.13 ± 0.02	4.60 ± 0.21
Cellulose III	97.71 ± 1.62	0.14 ± 0.01	11.30 ± 0.04

Here, $k_{\text{on}}^{*}$ is a pseudoadsorption rate constant, which is the product of true $K_{\text{on}}$ and the free protein concentration, whereas *k*_off_ is the true desorption rate constant. Fitted parameter means and standard deviations from two replicate assays are reported here. Sauerbrey equation was used to obtain the mass of adsorbed protein on cellulose film (or as total number of bound molecules upon achieving full binding saturation as represented by “A” here) using the frequency change at third overtone. The equations used for raw QCM-D data fitting are shown in SI Appendix Experimental Procedures section. Representative QCM-D sensorgrams are shown in Figure S10.

### Single-molecule CBM–cellulose bond rupture assay reveals complex multimodal nature of CBM binding

**Figure 7:**
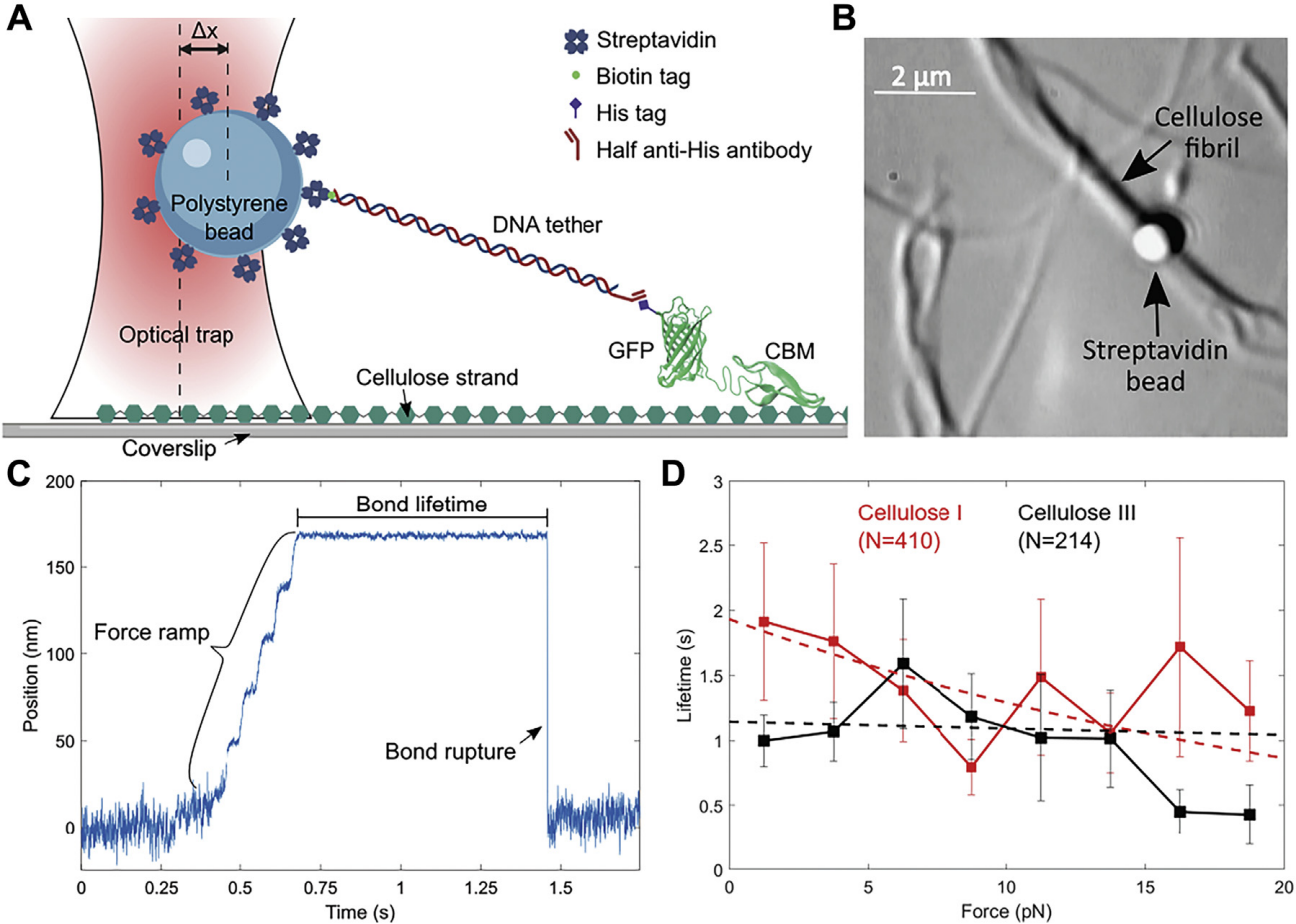
**Optical tweezers–based single-molecule bond “rupture” assay reveals the multimodal nature of CBM1–cellulose binding interactions.***A*, schematic of rupture assay setup (not to scale) is shown here where a streptavidin-coated bead is tethered to a single His-GFP–labeled CBM1 *via* a DNA linker containing an anti-His antibody Fab and a biotin tag on opposite ends. The biotin end specifically binds to streptavidin, whereas the ani-His antibody Fab specifically binds to the histidine tag of the GFP-labeled CBM1. Here, Δx represents that the bead displacement from the trap center. Figure created with BioRender.com. Published structures of CBM1 (Protein Data Bank code: 1CBH) and GFP (Protein Data Bank code: 2B3P) were used in this rendering. *B*, bright-field image of rupture assay showing Cladophora-based cellulose microfibrils localized on the glass cover slip. CBM–cellulose binding is facilitated by moving the optically trapped bead close to the fiber. Bead position is tracked by a detection laser as force is loaded across the bond. *C*, representative position trace for a single CBM–cellulose rupture event showing bond lifetime, and a single rupture is shown here. *D*, bond rupture force *versus* bond lifetime relationship for the CBM1–cellulose interaction on Cladophora cellulose I (*black*) and cellulose III (*red*) is shown. Lifetimes were binned at 2.5-pN intervals. Weighted single exponential fits are shown as *dashed lines*. Error bars depict standard error from the reported mean for each bin. N represents the total number of CBM–cellulose bond rupture events measured for each substrate. Additional supporting raw data scatterplots can be found in Figures S11–S14.

Here, we designed an optical tweezers–based CBM–cellulose bond rupture assay under applied force to systematically characterize the binding behavior of CBM1 (from Cel7A) toward Cladophora cellulose I and cellulose III. Our tweezer CBM–cellulose assay design is similar to the Cel7A enzyme motility assay as reported in Figure 2*A*. Here, instead of Cel7A, GFP-CBM1 was tethered *via* a 1010-bp DNA tether and attached to a 1.09-μm streptavidin-coated polystyrene bead (Fig. 7*A*). Cellulose fibers were affixed to a glass coverslip. For each single CBM–cellulose rupture assay run, individual beads were optically trapped and placed in the immediate vicinity of individual cellulose microfibers to facilitate a noncovalent CBM–cellulose bond formation (Fig. 7*B*). Upon stable noncovalent bond formation, the stage was moved to a fixed position to pull the DNA tether taut and exert a force on the CBM–cellulose bond. Total bond lifetime and rupture force were then calculated for each individual CBM–cellulose interaction till bond rupture took place (Fig. 7*C*). Hundreds of rupture events from individual assay runs were pooled and binned at 2.5-pN intervals for cellulose I and cellulose III to generate force-lifetime distribution plots (Fig. 7*D*). Raw force-lifetime scatterplots are provided in Figure S11*A*. Averaging all rupture events, we find that the mean lifetime of CBM1 binding to cellulose I was 1.41 ± 0.20 s (SEM; N = 410) and to cellulose III was 1.11 ± 0.12 s (SEM; N = 214). Since the bond rupture lifetime under applied force is related to the equilibrium binding off-rate, our rupture assay results are corroborated by the weaker binding affinity of CBM1 estimated by both the pull-down assay dataset as well as the PMF calculations. Of more importance, our bond rupture mean lifetime results suggest that simple one-site Langmuir adsorption models are more appropriate than complex multisite adsorption models to estimate the marginal differences in CBM1 binding affinity for distinct cellulosic allomorphs. Note that the standard deviation of lifetimes of the CBM1–cellulose I and CBM1–cellulose III bonds were 4.12 and 1.82 s, respectively. Although marginal differences can be seen at the lowest (0–2.5 pN) and highest (17.5–20 pN) rupture force ranges, one-way ANOVA test suggests that the lifetime dataset over the entire rupture force range is not statistically different (Fig. S11*B*).

Furthermore, although the average lifetimes show different profiles, there was also a broad spread in the distribution of observed bond lifetimes with a great deal of overlap between cellulose I and cellulose III indicating that multiple binding states with distinct characteristic bond lifetimes are possible for CBM1 binding to both cellulose I and III. As seen previously for protein–ligand interactions in other single-molecule studies (45), CBM–cellulose binding was expected to show classic slip-bond behavior; *i.e.*, as the rupture force increases, the total bond lifetime decreases. However, fits to the force-lifetime distribution failed to converge to a single exponential decay suggesting that multiple binding modes are likely present for CBM–cellulose. A classical unimodal slip bond would exhibit a single exponential decay (46); therefore, it suggests that CBM1 does not follow this simple model when interacting with either cellulose allomorph. Binding of CBM1 on cellulose instead revealed a spread with a more complex multimodal and heterogenous binding behavior. This multimodal distribution was independent of the source of cellulose, and similar results were also seen with filter paper–derived cellulose fibrils (Fig. S12). We also performed controls to test for artifacts associated with full anti-His antibody *versus* Fab fragment binding, but there was no significant difference seen in the multimodal distribution of the force-lifetime results (Fig. S13). Of interest, the multimodal distribution of the force-lifetime was sensitive to the CBM structure as illustrated by the differences in rupture force-lifetime distribution seen for wildtype CBM1 and its Y31A mutant, which has a minor modification to the planar aromatic binding residue (Fig. S14). Although the overall lifetime dataset over all rupture forces tested shows no significant difference based on the one-way ANOVA result, there seems to be significant difference in the bond lifetimes over certain rupture force ranges. The Y31A mutation is known to significantly lower CBM1 bulk-ensemble binding affinity toward native cellulose I (39), but it is unknown how this single mutation impacts the processive motility of the full-length Cel7A enzyme. Although these slight differences in CBM bond lifetimes might contribute to the reduced single-molecule velocity or initial binding commitment of Cel7A, the interactions of the CD with this substrate may play an equally important role.

**Figure 8:**
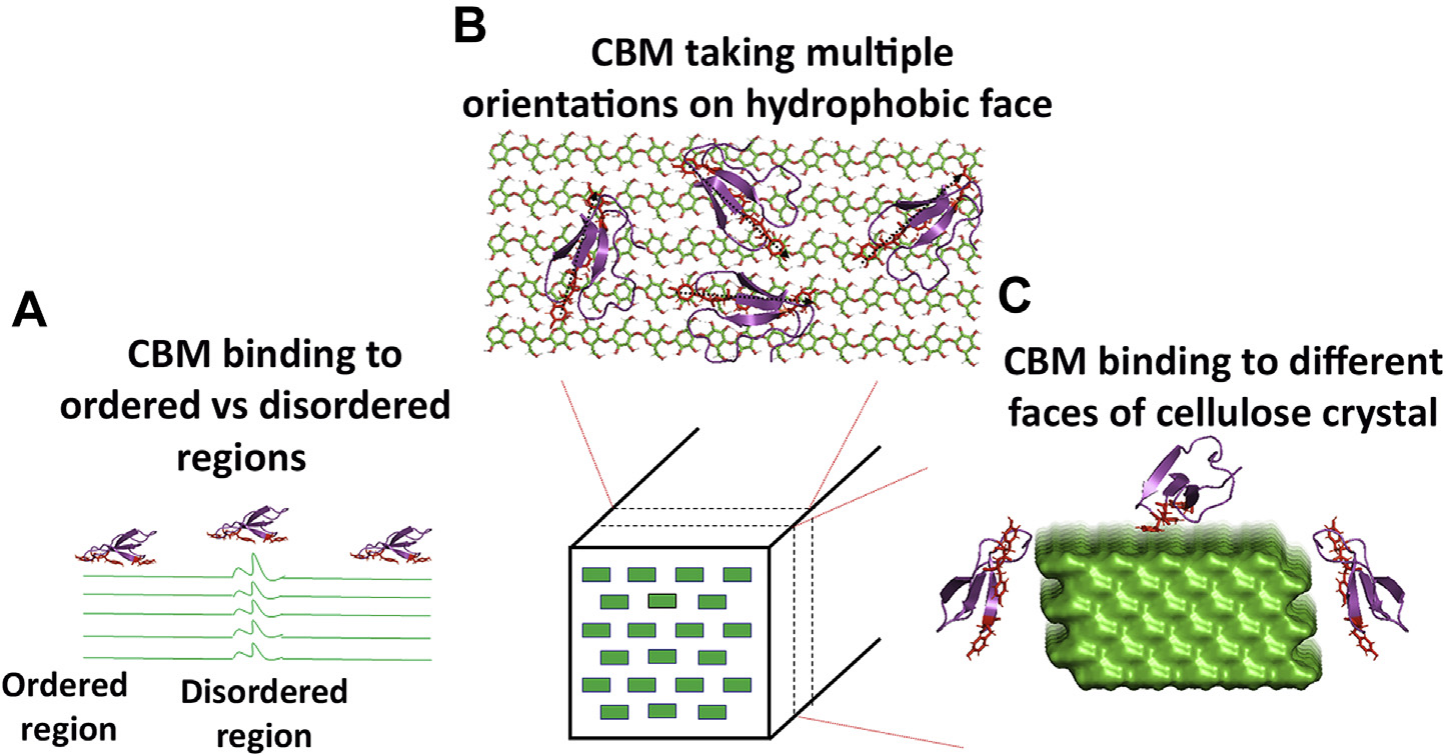
**Schematic outlining the three possible classes of binding sites theoretically accessible by CBM1 on native cellulose I fibers or crystal surface.***A*, cellulose chains are considered to possess local regions of disorder (also termed amorphous regions), and it is likely that the binding free energy to more ordered (or crystalline) regions is slightly different from that of highly disordered regions and hence be regarded as different classes of binding sites. *B*, previous molecular simulation studies show that the hydrophobic face of cellulose crystal is the preferred binding face for type A CBMs such as CBM1 (34). However, it is likely that the CBM possesses multiple binding orientations with respect to a cellulose chain due to nonspecific hydrophobic interactions which drive CBM–cellulose binding. *C*, in addition, although molecular simulations predict that the hydrophobic face is the “preferred” crystal face for CBM binding on microsecond time scales, transmission electron microscopy studies have shown the possibility of CBM binding to various other faces of the cellulose crystal (48). Overall, it is likely that the combination of all these potential binding sites, depending on cellulose source and overall ultrastructure, leads to the heterogeneity observed in binding of CBMs to distinct cellulose allomorphs. Here, CBM1 (Protein Data Bank code: 1CBH) from Cel7A was used to generate the figure.

We speculated that the observed multimodal distribution seen for the force-lifetime dataset indicates multiple classes of overlapping binding modes with contributions from different cellulose substructures (47), namely, crystalline regions with varying degrees of disorder, different crystal binding faces (48), and varied binding orientation/modes of CBM binding on the hydrophobic face of crystalline cellulose (as summarized in Fig. 8). However, owing to the highly crystalline nature of our Cladophora-derived cellulosic substrates (with ∼90%–95% crystallinity index) and the previous observations that CBM1 likely binds predominantly to one preferred cellulose crystalline face (48), we hypothesize that the multimodal distribution in the force-lifetime dataset could also arise from multiple equilibrium binding modes of CBM1 with distinct orientations on the preferred cellulose binding surfaces (see Discussion section below and supporting Monte Carlo simulation results highlighted in Fig. S16 and Table S3). It is also likely that some of these CBM orientations are productive for catalysis, whereas some orientations are nonproductive. For Cel7A to perform a successful processive step, the CBM needs to step in tandem with the CD along a cellulose chain (35). However, if the CBM orients itself in nonproductive orientations (across adjacent cellulose chains, for instance), we speculate that this could lead to increased dwell times for full-length Cel7A as seen on cellulose III. Additional mutant full-length Cel7A assays are necessary to unravel molecular origins of such multimodal binding behavior during cellulase catalytic turnover cycles.

## Discussion

Pretreatments can increase cellulose accessibility to facilitate efficient enzymatic saccharification (49). Extractive ammonia (EA) pretreatment converts cellulose I to cellulose III to reduce biomass recalcitrance toward enzymatic hydrolysis. EA pretreatment achieves cellulosic biomass hydrolysis yields equivalent to its precursor ammonia fiber expansion pretreatment but with 60% lower enzyme loading requirements (*e.g.*, 18.75 mg enzyme/g cellulose for ammonia fiber expansion *versus* 7.5 mg/g for EA-treated biomass hydrolyzed using commercial enzyme mixture consisting of 50% C.Tec2, 25% H.Tec2, and 25% Multifect Pectinase on a total protein basis) (12). However, there is a need to further reduce total enzyme loading equivalent to the range employed in a commercially viable corn starch liquefaction process using amylases (*e.g.*, less than 1 mg amylase/g starch). One approach to reduce enzyme loading is to identify the potential rate-limiting enzymes in a complex cocktail critical for cellulose III hydrolysis. Endocellulases have been identified to show improved activity toward cellulose III, at various enzyme loadings tested, but they concomitantly show lower binding to the substrate, unlike towards cellulose I. But it is surprising that exocellulases like Cel7A (*T. reesei*) and Cel6B (*T. fusca*) have mostly shown lower or comparable activity on cellulose III *versus* native cellulose I, particularly at ultra-low enzyme loadings as reported in this study. Although this is not detrimental to the action of cellulase enzyme mixtures, as both fungal and bacterial derived endo- and exocellulase mixtures have shown overall improved activity (up to 10-fold as reported here) toward cellulose III *versus* cellulose I largely owing to increased endo–exo cellulase synergy (14, 15), there is clearly room for making improvements in enhancing processive cellulase activity toward cellulose III. Both endo- or exocellulases were previously reported to exhibit lowered binding toward cellulose III during saccharification. Although these results can be explained based on the Sabatier principle recently applied to modeling cellulase action on cellulose (19), since tighter cellulase binding to cellulose need not always correspond to improved activity (13), we still lack a first-principles mechanistic basis for the reduced binding of most full-length cellulases observed to date toward nonnative cellulose III allomorph using advanced optical tweezers–based single-molecule assays (22).

Here, we developed and applied a single-molecule optical tweezer–based assay that allowed us to distinguish the initial enzyme binding commitment to repeated processive motility cycles that forms the basis of catalytic turnover of processive cellulases like Cel7A. Our results indicate that full-length Cel7A show impaired single-molecule motility commitment toward cellulose III, which was hypothesized to arise owing to unstable initial binding predominantly driven by the CBM. This hypothesis is also in alignment with reduced overall binding observed previously of full-length type A CBM-based cellulases to cellulose III (13, 15). Second, the Cel7A motility assay showed marginally lower processive velocity on cellulose III than cellulose I, which is consistent with the classical bulk activity assays conducted at very low enzyme loadings that confirmed no significant difference in Cel7A activity on either cellulose allomorph. Although the difference between velocities and dwell times between the two allomorphs seems minor, we clearly noticed differences in the binding stability of Cel7A to cellulose III. Previous AFM and super-resolution fluorescence based Cel7A single-molecule motility measurements on cellulose have been conducted at very high enzyme loadings where multiple Cel7A proteins often interact with each other to literally “push” stuck enzymes out of their way in so-called Cel7A traffic jams on the cellulose surface (22, 24). It is possible that similar protein–protein interactions play an important role in aiding cellulose deconstruction at higher cellulase loadings and could explain why higher Cel7A loadings result in higher activity toward cellulose III. Furthermore, Igarashi and co-workers (24) have speculated that, since cellulose III has a modified crystal surface with a larger exposed protein-binding surface than cellulose I, it is possible that multiple bound Cel7A enzymes can simultaneously deconstruct cellulose III to give improved hydrolysis yield but only at higher enzyme loadings (*e.g.*, ∼25–50 mg/g). However, in our tweezer-based Cel7A assays we only monitor one bound enzyme molecule at a time, without any interaction effects from other freely diffusing or surface-bound enzymes. Our motility results are therefore representative of single-enzyme behavior that would be expected as we drive down the total protein-to-cellulose loading to extreme enzyme-limiting conditions (*e.g.*, under 0.5 mg/g). It would be interesting to study if addition of exogenous, freely diffusing exocellulases would impact the observed motility of the DNA-bead tethered single-Cel7A enzyme in our assay to unravel the impact of protein–protein interactions on improved catalytic activity on cellulose III. Future studies could also explore the role of possible allosteric effects on processive cellulase CD interactions with the cellulose allomorph surface to help explain the slightly increased stalling of the enzyme and increased back-stepping seen for Cel7A on cellulose III in particular. Regardless, here we hypothesized that the initial binding commitment of cellulases driven by CBMs is likely a key limiting step to kick starting efficient cellulose saccharification.

Understanding CBM–polysaccharide binding interactions is critical to gaining mechanistic insights into biomass conversion (50, 51, 52) and developing more efficient industrial-grade enzymes (53, 54). Although molecular simulations have been employed to study specific steps of the Cel7A cellulase processive cycle such as chain decrystallization (55), glycosylation (20), deglycosylation (21), and dissociation (56), the role of CBMs in initial motility commitment of CDs has not yet been studied in detail (9, 12). From an evolutionary standpoint, type A CBMs and cellulase CDs have naturally evolved to breakdown native cellulose I (57) but not cellulose III. Therefore, here we used classical CBM–cellulose pull-down binding assays, molecular dynamics simulations, and optical tweezer–based bond rupture assays to obtain a comprehensive understanding of the binding interactions of a model CBM1 (isolated from Cel7A) toward cellulose I and cellulose III. Classical pull-down bulk ensemble binding assays have been employed extensively to study protein binding to insoluble polysaccharides like cellulose (40). Like previous reports, various adsorption models such as Langmuir one-site/two-site and Langmuir-Freundlich models were applied to extract phenomenological model-based parameters for CBM1 binding toward both cellulose allomorphs. Regardless of the change in binding affinity, we mostly observed a drop in the total available binding sites available for CBM1, which suggests that the surface properties of cellulose allomorph have a significant impact on binding and recognition by CBMs/cellulases (15). A similar reduction in CBM binding was observed for another cellulose allomorph (*i.e.*, cellulose II) using QCM-D as well (58), suggesting most CBMs likely display subtle differences in binding interactions toward distinct cellulose allomorphs. We further extended our study to other model type A CBMs (*e.g.*, CBM3a, CBM64) and confirmed reduced CBM binding partition coefficient observed toward cellulose III for all CBMs tested so far. Reduced mutant CBM3a and CBM64 binding toward distinct cellulose allomorphs further highlights the complex nature of CBM–cellulose binding interactions and its relationship to appended CD activity as shown in another recent study (59). Moreover, Langmuir adsorption models are applicable under some key assumptions (*e.g.*, complete reversibility of protein–ligand binding, absence of bound protein structural deformation or interactions with other bound proteins, absence of overlapping binding sites, and complete surface saturation achieved at the maximum protein loading tested), which can often lead to possibly spurious conclusions resulting from such analyses (60). We also studied a small-molecule CBM-surrogate such as calcofluor white to characterize its binding parameters toward distinct cellulose allomorphs to show that calcofluor also has lower affinity and binding sites available for adsorption to microcrystalline Avicel based cellulose III *versus* native cellulose I. But even for a simple stilbene-based derivative like calcofluor, we observed a concave upward behavior in Scatchard plots, which is indicative of overlapping binding sites and/or multiple classes of nonequivalent binding sites (see Fig. S15 and SI Appendix for supporting discussion). These results highlight the complexity of studying CBM–cellulose interactions using simple Langmuir-based adsorption models and the inherent heterogeneity of the substrate-binding sites that makes it challenging to gain deeper mechanistic insights from classical protein–polysaccharide pull-down assays alone.

To corroborate our results from CBM1 pull-down binding assay analyses, we performed molecular dynamics (MD) simulations to characterize CBM1 binding. MD simulations have been employed extensively to study cellulolytic enzymes (20, 61, 62, 63, 64) and offer detailed atomistic insights into the highly heterogeneous binding interactions of proteins with insoluble polysaccharides. MD simulations have revealed structural and dynamical features of cellulose III such as hydrogen bonding, solvent accessible surface area, and single-cellulose chains decrystallization free energy (15, 55). Few studies have been carried out to understand CBM binding to cellulose, whereas most work has been restricted to native cellulose I (34, 35, 65). Our PMF calculations from molecular simulations revealed that the binding free energy for CBM1 toward highly crystalline cellulose III is marginally (∼1.2-fold) lower than toward cellulose I, confirming our analysis of binding assays based on Langmuir one-site model. The decrease in PMF estimated binding free energy is over 3-fold for more disordered (*i.e.*, less crystalline) forms of cellulose III that are expected to be produced under certain low-temperature ammonia pretreatment conditions (27), but this was not the case in this study. Hence, these results further suggest the use of simpler adsorption models like Langmuir one-site model that yield a more representative average binding affinity and available binding sites, instead of using overparameterized multisite Langmuir type models that could result in estimation of spurious binding parameters. MD simulations also provided atomistic insight into the molecular origins of reduced CBM1 binding to cellulose III owing to improper Y5/Y31/Y32 aromatic residue stacking interactions and steric clashes with the jagged cellulose surface chains. Since C6-hydroxymethyl groups on the surface-exposed cellulose chains in native cellulose I are often highly disordered to adopt additional *gauche*-*trans-* and *gauche-gauche-*type rotameric states, it is likely that improper stacking of aromatic residues of CBMs with distinct cellulose crystal faces will impact the binding stability to even some forms of native cellulose allomorphs. Of interest, CBM1 also displayed a preferred noncanonical orientation on the surface of cellulose III, which could impair Cel7A CD motility. This could explain why intact Cel7A displayed impaired motility on cellulose III with longer dwell times than cellulose I, but more experimental work is needed with CDs alone to rule out any allosteric effects that could impact motility as well. Engineering CBMs to reduce steric clashes and enable preferred canonical orientation to aid in efficient cellulase processivity is an area where future advancements can be made using rational structure-guided enzyme engineering strategies.

Although classical pull-down binding assays and MD simulations explain how the impaired cellulase motility commitment on cellulose III could arise from CBM1, the CBM1–cellulose binding/unbinding forces relevant to the processive motility cycles of Cel7A were unclear. Hence, we developed and applied a single-molecule noncovalent bond rupture assay to characterize CBM–cellulose binding interactions under applied force. Single-molecule force spectroscopy has been employed previously to distinguish the nature of protein–ligand bonds (45) and infer multimodality or conformational transitions involved in protein–ligand binding interactions (66). However, the application of AFM-based force spectroscopy to study CBM–cellulose binding has revealed challenges in distinguishing specific *versus* nonspecific interactions (67). Here, we developed a novel single-molecule optical tweezer–based bond rupture assay with piconewton (pN) force resolution and millisecond (ms) time resolution (66) to understand the heterogeneity of CBM–cellulose unbinding behavior under the application of force. The ultimate goal of the bond rupture assay was to understand the role of CBM1 binding in the anomalous processive motility of Cel7A on cellulose III. CBM1 showed multimodal force-lifetime behavior toward both cellulose I and cellulose III with no statistically significant differences in mean bond lifetimes except under extreme force ranges where the differences were slightly more pronounced. Of interest, the rupture assay mean bond lifetime of CBM1 with filter paper–derived cellulose I fibrils was significantly higher (by ∼2-fold) than that of Cladophora cellulose I. Overall, these results highlight how subtle differences in cellulose fibril ultrastructure can play an important role in impacting CBM binding dynamics at the single-molecule level. Rupture assay bond lifetimes estimated from dynamic force spectroscopy assays can be used to predict protein–ligand unbinding off-rates that relate directly to the classical binding affinity constant (68). Considering the mean bond lifetime for CBM1 was only marginally higher for cellulose I *versus* cellulose III, these results further suggest that a simple one-site Langmuir adsorption model used to fit the pull-down binding assay data would be more appropriate than other multisite models that predict much larger differences in binding affinity. Our single-molecule CBM–cellulose bond rupture assay suggests that the binding behavior cannot be explained by the presence of just one or even two classes of unique and independent binding sites. However, fitting a high-quality binding assay dataset to a simple Langmuir one-site model can still yield a global average affinity constant that arises from a combination of binding sites or modes, rather than data overfitting *via* a two-site or more complex binding models. Our analysis also suggests the use of Langmuir one-site models to obtain binding parameters when studying protein–polysaccharide binding interactions, while also using complementary approaches to cross-validate the molecular-level origins of relative differences in binding behaviors observed for distinct ligands and/or protein mutants. Recent reports on even simpler protein–ligand systems like streptavidin–biotin suggests that ligand unbinding undergoes transition across multiple intermediate states as a function of the loading rate (*i.e.*, applied force), unlike the classical two-state models, to explain the long lifetime of the complexes (69). Therefore, further studies are necessary for the CBM–cellulose system at multiple loading rates. We speculate that the nonproductive binding of CBMs with high bond lifetimes could increase CD dwell time, and mutant CBMs/cellulases should be analyzed to test this hypothesis further.

Finally, we were interested to see if it would be theoretically possible to explain the multimodality observed for CBM–cellulose force-lifetime distributions using a simple geometrical probability-based model whereby the CBM is hypothesized to survey multiple binding orientations on the hydrophobic face of cellulose, assuming that different orientations would give a distinct bond lifetime at a given applied force. We were inspired by the classical Buffon needle problem and therefore developed a simple model based on this original problem to predict the probabilistic distribution of the orientation of CBM proteins on the surface of cellulose (70). Here the size of our needle is interpreted as the physical length of the planar binding motif surface (*e.g.*, Y5-Y31-Y32) known to participate in cellulose binding, whereas the distance between the adjacent cellulose chains on the hydrophobic binding surface is equivalent to the distance between the parallel lines between which the needle can fall on along the line axis or across the axis between crossing multiple lines based on the original Buffon problem. Our Buffon needle model for the wildtype CBM1 predicted that the distribution of CBM1 binding states should mostly align along the cellulose chain axis *versus* across the chain axis under the assumption that these states are energetically equivalent, as discussed in the SI Appendix Results and Discussion section (see Fig. S16). Alignment of the CBM needle along the cellulose chain axis is also supported by previous MD simulations (35), lending some credence to this overly simplistic geometrical interpretation of the CBM–cellulose binding problem. Of interest, “shortening” of the effective CBM needle length (*i.e.*, by mutation of Y31A for CBM1) increased the likelihood of along the cellulose chain/axis binding events as predicted by the Buffon model. It was interesting to note that our single-molecule tweezer–based CBM–cellulose rupture assay also indicated a 2.6-fold significantly higher rupture bond lifetimes (in 10–15 pN rupture force range) for the Y31A mutant compared with the wildtype CBM1 on cellulose I, suggesting the intriguing possibility that a subset of the force-lifetime data observed could be representative of specific CBM1 orientations on the cellulose surface. A similar flanking aromatic residue mutation on other type A CBMs planar binding sites was recently shown to also enhance engineered endocellulase catalytic toward native cellulose, possibly owing to reduced nonproductive mutant enzyme binding driven by particular binding orientations (59). Future work combining site-directed mutagenesis of CBMs, force spectroscopy rupture assays, and MD simulations is necessary to test the impact of specific CBM-binding motif mutations on altering certain binding modalities, as analogously illustrated by Jobst *et al.* (71) for the cohesin–dockerin binding system.

Binding modules like CBM1 play an oft-neglected synergistic role in the association of Cel7A CD to cellulose that likely fine-tunes the subtle balance between productive *versus* nonproductive binding (72). Our motility assays have, for the first time in the reported literature, captured the early steps of full-length cellulase complexation (also called Cel7A processive cycle motility commitment) to a cellulose reducing end before the catalytic processive cycle begins. Future work will address the role of CBMs in both the association and dissociation processes of full-length cellulases to obtain a better understanding of the relationship between binding affinity and overall catalytic efficiency for processive cellulases (19). Our work has also shown that, although the exact stalling force for halting processive cellulases like Cel7A likely exceeds 30 pN to prevent cellulase motility entirely (26), it is possible that particular CBM binding orientations on the cellulose surface could hinder cellulase motility or processive activity. However, the connection between data collected from single-enzyme motility/rupture assays, enzyme binding/activity, and enzyme–substrate structure dynamics still needs to be more clearly established. In addition, future work should address the interplay of CBM-driven binding affinity and hydrolytic activity of multimodular cellulases, using biochemical assays similar to those reported in a recent study that applied the Sabatier principle to characterize interfacial cellulose hydrolysis by bound cellulases (19). It is likely that the lower binding and improved activity of endocellulases and exocellulases toward cellulose III at certain enzyme loadings is in accordance with the Sabatier principle.

## Experimental Procedures

See supporting information for all experimental and computational methods used here.

## Data availability

All raw data contained within the article are available upon request from the corresponding author (Dr Shishir Chundawat, Rutgers University, shishir.chundawat@rutgers.edu).

## Supporting information

This article contains supporting information.

## Conflict of interest

S. P. S. C. declares competing financial interest having filed two patent applications on pretreatment processes to produce cellulose III–enriched cellulosic biomass for biofuels production (US20130244293A1 and WO2011133571A2). All other authors declare that they have no competing interests.
