# Supplementary material for: Molecular origins of reduced activity and binding commitment of processive cellulases and associated carbohydrate-binding proteins to cellulose III
Source: J Biol Chem. 2021 Feb 19;296:100431. doi: 10.1016/j.jbc.2021.100431 (PMC8010709; doi:10.1016/j.jbc.2021.100431)
Supplement: Supplementary file 1 — Supplementary information [file mmc1.pdf]

Supplementary Information (SI) for

Molecular origins of reduced activity and binding commitment of processive cellulases and associated carbohydrate-binding proteins to cellulose III

Shishir P. S. Chundawat,[a*] Bhargava Nemmaru,[a] Markus Hackl,[a] Sonia K. Brady,[b] Mark A. Hilton,[b] Madeline M Johnson,[b] Sungrok Chang,[b] Matthew J. Lang,[b,c] Hyun Huh,[d] Sang-Hyuk Lee,[d] John M. Yarbrough,[e] Cesar A. López,[f] Sandrasegaram Gnanakaran[f]

[a]Department of Chemical & Biochemical Engineering, Rutgers The State University of New Jersey, Piscataway, New Jersey 08854, USA.

[b]Department of Chemical and Biomolecular Engineering, Vanderbilt University, Nashville, Tennessee 37235, USA.

[c]Department of Molecular Physiology and Biophysics, Vanderbilt University, Nashville, Tennessee 37235, USA.

[d]Department of Physics and Astronomy, Rutgers The State University of New Jersey, Piscataway, New Jersey 08854, USA.

[e]Biosciences Center, National Renewable Energy Lab, Golden, Colorado 80401, USA.

[f]Theoretical Division, Los Alamos National Laboratory, Los Alamos, New Mexico 87545, USA.

Corresponding Author Name: Shishir P. S. Chundawat* (ORCID: 0000-0003-3677-6735)

Corresponding Author Email: shishir.chundawat@rutgers.edu

This PDF file includes:

Supplementary text (Experimental Procedures and Results)
Figures S1 to S16
Tables S1 to S3
Legends for Movies S1 to S3
SI References

Other supplementary materials for this manuscript include the following:

Movies S1 to S3

SI Appendix Experimental Procedures:

Crystalline Cellulose Isolation and Anhydrous Liquid Ammonia Pretreatment: High crystallinity cellulose I (called native Cladophora cellulose I) from *Cladophora* sp. (*Cladophora glomerata*) was isolated and characterized as described previously (1). High purity (>98% cellulose content, dry weight mass basis or dwb) plant-derived microcrystalline cellulose I (called Avicel cellulose I) was purchased from Sigma-Aldrich (Avicel PH-101, Lot No. BCBD6923V). These native cellulose samples were used to generate respective Avicel or Cladophora derived crystalline cellulose III using a suitable anhydrous liquid ammonia based pretreatment process (2, 3). All cellulose III samples were kindly treated by Dr. Leonardo Sousa using a typical anhydrous liquid ammonia treatment protocol as highlighted elsewhere (3). Briefly, cellulose III was prepared in a high-pressure stirred batch reactor at 90 °C for 30 min (for Avicel) or 4 h (for Cladophora) residence time using at least a minimum 6:1 anhydrous liquid ammonia-to-cellulose loading ratio (dwb). The reactor pressure was maintained constant at 1000 psi using nitrogen gas during the pretreatment, and ammonia was slowly evaporated from the reactor through a venting valve after the desired residence time. During this evaporation process, the temperature of the reactor was slowly decreased and kept stabilized at 25 °C. The treated cellulose sample was then removed from the reactor and placed overnight in the fume hood to evaporate any residual ammonia. All treated cellulose samples were stored at 4 °C in a zip sealed bag prior and were used directly without any further drying.

Cellulose Characterization using XRD & FT-Raman Spectroscopy: Details regarding the X-ray diffraction (XRD) method and data analysis methods/results are provided elsewhere (2, 3). Briefly, XRD was performed on an X-ray diffractometer with beam parallelized by a Gobel mirror (D8 Advance with Lynxeye detector; Bruker, Bruker AXS Inc., Madison, WI, USA). CuKα radiation (wavelength = 1.5418 Å) was generated at 40 kV with 40 mA current and the detector slit was set to 2.000 mm. Samples were analyzed using a coupled 2θ/θ scan type with a continuous PSD fast scan mode. The 2θ started at 8.000° and ended at 30.0277° with increments of 0.02151°, while θ started at 4.0000° and ended at 15.0138° with increments of 0.01075°. Step time was 1.000 s (i.e., 1025 total steps, effective total time 1157 s per run). Dry cellulose samples (approximately 0.5 g) were placed in a specimen holder ring made of PMMA with 25 mm diameter and 8.5 mm height, rotating at 5 degrees per minute during analysis. Cellulose crystallinity was estimated based on the Segal peak height (for Cladophora derived samples) and amorphous peak deconvolution based methods (4, 5). Please note that Miller indices used in this paper for each contributing predominant diffraction peak/s conform to the convention with 'c' as the fiber axis, a right-handed relationship among the axes and the length of a<b, as recommended recently by Alfred French (6), to avoid confusion with other naming conventions. Briefly, for the XRD Segal peak height method, cellulose crystallinity index was calculated from the ratio of the height of the (110) or (200) plane equatorial reflection peak and the height of the minimum between the (110) or (200) and (010) or (110) plane equatorial reflection peaks for Cladophora or Avicel PH-101 cellulose I, respectively. For cellulose III, cellulose crystallinity index was calculated from the ratio of the height of the (100) plane equatorial reflection peak and the height of the minimum between the (100) and (002) plane equatorial reflection peaks. Note that, the three main peaks for native Cladophora cellulose I one-chain triclinic unit cell have Miller indices of (100), (010) and (110), which are the counterparts to the (1-10), (110) and (200) peaks of Avicel PH101 cellulose I pattern. Peak deconvolution methods have been used extensively to calculate cellulose crystallinity index (5, 7–9). Avicel derived cellulose I and III samples were recently analyzed using the amorphous peak deconvolution method (10). XRD peak deconvolutions were carried out using PeakFIT (Version 4.12, Systat Software Inc, San Jose, CA) as described elsewhere (2, 5). For all peak deconvolutions F values are always > 30,000 while R-squares > 0.999.

Additional supporting details regarding the FT (Fourier Transform) Raman based spectroscopic characterization methods/results are provided here as well. Briefly, a MultiRam FT-Raman spectrometer (Bruker) was used to collect Raman spectra for cellulose samples. The FT-Raman spectrometer was equipped with a 1064-nm 1000-mW Nd:YAG laser. For Raman analysis, cellulose pellets were first prepared from either air-dried or lyophilized samples prior to analysis. In most cases, spectra with high signal-to-noise (S/N) ratios was obtained upon using a 660 mW laser power setting and collecting over 512 scans per sample. The spectra were converted to ASCII format and

exported to Microsoft Excel for direct plotting/analysis. The interconversion of cellulose I to III was confirmed based on previously published reports using Cladophora or cotton linters derived cellulose allomorphs (3, 11–14). Peak assignments of the vibrational spectrum of cellulose I and III have been described elsewhere (3, 13, 15). Briefly, 250-550 cm^{-1} region for cellulose has predominant group motions attributed to skeletal-bending modes involving C-C-C, C-O-C, O-C-C, and O-C-O internal bond coordinates. The 550-750 cm^{-1} region corresponds to mostly out-of-plane bending modes involving C-C-C, C-O-C, O-C-O, C-C-O, and O-H internal bond coordinates. The peaks around 900 cm^{-1} are shown to involve bending of H-C-C and H-C-O bonds localized at C-6 atoms of the hydroxymethyl group. The 950-1200 cm^{-1} region corresponds to mostly stretching motions involving C-C and C-O internal bond coordinates. The 1200-1500 cm^{-1} region corresponds to mostly bending motions involving H-C-C, H-C-O, H-C-H, and C-O-H internal bond coordinates. The region of 1400-1500 cm^{-1} for cellulose has been shown to be particularly sensitive to the CH_2 scissor bending modes that are sensitive to the *Trans-Gauche* or TG (1480 cm^{-1}) and *Gauche-Trans* or GT (1460 cm^{-1}) conformations of the C6-hydroxymethyl group (13).

Enzymatic hydrolysis of cellulose allomorphs: All Cladophora cellulose samples were subjected to enzymatic hydrolysis using a commercial cellulase cocktail at 0.5% glucan loading in a 1 ml reaction volume. All hydrolysis assays were carried out in 2 mL flat-bottomed microcentrifuge tubes using 5 mg of pre-weighed cellulose suspended in 500 µL of 50 mM Na-Acetate buffer, at pH 5.0 (for Cellic C.Tec2 (Novozymes A/S, Denmark)), along with suitably diluted stock enzyme solution to achieve desired enzyme loadings (i.e., mg total enzyme loaded per gram of added cellulose per well). The total Cellic C.Tec2 enzymes (Novozymes A/S, Denmark) loadings used during enzymatic hydrolysis was fixed at 5 mg/g glucan loading, unless specified otherwise. The protein concentration (193 mg/ml) for the C.Tec2 enzyme stock solutions was determined using the Kjeldahl method (16). Sodium azide was added to prevent any microbial growth (0.1% w/v final concentration). All tubes were incubated at 50 ˚C in an orbital shaking ThermoMixer (Eppendorf) incubator set at 1000 RPM for the desired saccharification time (0-96 h). A similar procedure was used for enzymatic hydrolysis using an equimolar mixture of purified Cel7A and Cel7B enzyme or Cel7A enzyme alone. For the Cel7A-Cel7B mixture experiment, the enzyme loading for Cel7A was kept at 10 mg enzyme per g cellulose and β-glucosidase was added to prevent inhibition by cellobiose. For the Cel7A activity assay experiment alone, three enzyme loadings were tested (0.5, 2.5, and 10 mg enzyme per g cellulose). Cel7A, Cel7B, and β-glucosidase were purified and isolated from commercial enzyme sources as outlined previously (17). The hydrolyzate supernatants were analyzed for total reducing sugar concentrations using the standard dinitrosalicylic acid (DNS) colorimetric assay as reported earlier (18). Briefly, 30 µl of the hydrolysate supernatant (w/wo 2-fold dilution) was incubated with 60 µl of DNS stock reagent in PCR tubes/plates at 95 °C for 5 min in an Eppendorf thermal cycler. After the PCR plates cooled down to room temperature, the DNS reaction mixture was transferred and diluted in DI water using a clear, flat-bottom microplate for finally measuring solution absorbance at 540 nm. Suitable reducing sugar standards (e.g., glucose standards ranging from 0.1–5 g/l) were included for the DNS assay. All hydrolysis experiments were carried out in duplicates. Error bars reported represent one standard deviation (±1σ) from mean values for replicate assays.

Sample preparation for AFM imaging of Cladophora CI and CIII: Approximately 5 mg of dried cellulose I (CI) and cellulose III (CIII) fibers derived from *Cladophora glomerata* were each added to a microtube and suspended in 1 ml of DI water. At first, the fibers were manually dispersed through pipetting the suspension up and down using a wide opening 1 ml pipette. Subsequently, the suspensions were sonicated for 1 minute (model FB705 Fisher Scientific, USA, settings 10% amplitude, 2 s on, 5 s off), then pipetted up and down until a segregation of the fibers was observed. Two hundred microliters (200 µl) of the resulting suspensions were transferred to new microtubes and filled up to 1 ml with DI water. The fibers were further fragmented by pipetting through a 1 ml pipette until all large aggregates were dispersed. The suspensions were stored at 4°C until use and resuspended prior to usage.

AFM imaging of Cladophora CI and CIII microfibrils: The microscope cover glasses (No. 1.5, 22x22mm, VWR, USA) were rinsed in the following order, DI water, acetone (NF/FCC grade, Fisher Scientific, USA) and DI water and then dried with a stream of nitrogen. Twenty microliters (20 µl) of cellulose I and III samples were each placed in the middle of the glass slide and dried over night at 50°C. Non-contact mode AFM measurements were carried

out with a Park systems NX10 AFM using non-contact cantilever (SSS-NCHR, Park Systems, South Korea) with a force constant of 42 N/m (specific range: 10-130) and a resonant frequency of 330 kHz (specific range: 204-497). For each of the substrates, 2.5 x 5 μm^2 sized areas were chosen at random places of the sample. Data were analyzed using the XEI software (Park Systems, South Korea).

Functionalization of beads for tweezer binding-rupture and motility assays: For the motility assays, purified Cel7A enzymes were tethered to polystyrene beads and assayed for their motility on Cladophora derived cellulose I and cellulose III based on identical methods reported earlier in our Cel7A tweezer motility study (1). For the rupture assays, CBM1 was tethered to polystyrene beads via the His_8-tag on the N-terminus of our purified GFP-CBM1 construct (details on GFP-CBM construct design and purification are provided below), with minor modifications from our previously published work (1). Using PCR, 1,010-bp DNA linkers were created from the M13mp18 plasmid template with a biotin tag on one end and an amine group on the other. The anti-His antibody was crosslinked to the amine group using a sulfo-SMCC (sulfosuccinimidyl 4-(N-maleimidomethyl)cyclohexane-1-carboxylate) intermediate. In the cases of using the anti-His Fab, the anti-His antibody was cleaved using 3-MEA (2-Mercaptoethylamine) before crosslinking. To functionalize the beads with GFP-CBM1, 1.09 µm streptavidin beads (Spherotech), biotin/anti-His functionalized DNA linkers, and His_8-tagged GFP-CBM1 constructs were incubated together in PBS at 4°C for 45 minutes on a rotator. After incubation, the beads were washed by spinning down at 9000 rpm for 3.5 minutes, removing the unreacted components in the supernatant, resuspending in 50 mM acetate buffer (pH 5.0), and sonicating for 2 minutes at 20% amplitude. This process was repeated two more times. Beads were functionalized such that, statistically, zero or one GFP-CBM1 molecule is bound to each bead. This was determined through serial dilution until a maximum of half the beads bound to cellulose fibers during the experiment.

Cellulose solution and slide preparation for tweezer binding-rupture and motility assays: Purified and dried cellulose samples (*Cladophora* based cellulose I or III) were used to create a heterogeneous cellulose mixture by first mixing the desired cellulose sample to deionized water in a 1 mg/mL ratio. The mixture was then sonicated for 2 minutes at 50% in a cup sonicator and vortexed for 15 s on high setting. The cellulose, still clumped at this point, was pulled up and down in solution with a 16-gauge syringe for 1-2 minutes before going back on the vortex for 15 s. These steps were repeated three times. The resulting mixture was then diluted in a 1:20 ratio by mixing 500 µL of the prepared solution with 500 µL deionized water. This slurry suspension was then stored at 4 °C. Whatman Grade 1 Filter Paper based cellulose stock suspension slurry was prepared as described previously (1), to be used for some control GFP-CBM1 binding-rupture assays. When preparing to load a slide, a small sample (~100 µL) of the stored cellulose mixture is removed from the stock and the cellulose pulled apart by sonicating for 2 minutes at 50% in a cup sonicated. This solution was directly loaded onto the glass slide. Slides are prepared by creating a 10-15 µL volume flowcell using a KOH etched coverslip and double-sided sticky tape. The stock cellulose solution (*Cladophora* based cellulose I or III) was then added to the flowcell and allowed to dry out in an oven at ~95 °C for an hour, allowing cellulose fibrils to non-specifically bind to the slide surface. The surface was then blocked with 10 mg/mL BSA in acetate buffer (pH 5.0) for 15 minutes to prevent non-specific sticking of the beads to the glass surface. Finally, the GFP-CBM1 functionalized beads solution was loaded onto the slide and the slide sealed shut. For the Cel7A motility assays, 0.75 µm non-functionalized polystyrene beads (Spherotech—PP-08-10) were allowed to nonspecifically adhere to the coverslip surface, in an incubation step before BSA blocking, to serve as fiducial markers allowing for instrumental drift tracking during data acquisition.

Single molecule tweezer binding-rupture assay data acquisition and analysis: CBM1 functionalized beads were trapped using a 1064-nm laser setup as described before (1), and placed alongside a surface-bound stationary fiber. Experiments were conducted at a fixed room temperature (21 °C). After position calibration and trap stiffness measurements, the bead was actively placed on a cellulose fiber roughly running along the axis of the microscope stage. Upon binding, the bead was centered, acquisition started, and a force applied to the tethered bead by stepping the piezo stage along the axis of the fiber. With force applied, the position of the bead is held until rupture. Once a tether is ruptured, it is sometimes possible to tether the bead to the fiber again, in which case, the same method of

force application is applied while data acquisition continues. Data were collected at a 3-kHz sampling frequency and then filtered with a 10-point exponential moving average before analysis. Custom Matlab codes were then used determine the rupture forces and the bond lifetimes of full ruptures. The force-lifetime data was binned every 2.5 pN and then we tried to fit the data to a single or a double exponential decay characteristic of a slip bond.

GFP-CBMs gene synthesis and cloning: *E. coli* codon optimized genes encoding CBMs, with additional flanking *AflII* and *BamHI* restriction sites, inserted into a standard pUC57-Kan vector were ordered from Genscript USA Inc (Piscataway, NJ). DNA sequences for all CBMs are provided in the table below. An *E. coli* expression vector pEC-GFP-CBM3a was kindly provided by the Fox lab (UW Madison). Sequence information regarding the family 3a CBM from *Clostridium thermocellum* expressed using this pEC vector have been published already (19). The pEC vector sequence map and strategies for primer design and CBM genes sub-cloning have been reported already (19, 20). Briefly, polymerase incomplete primer extension (PIPE) based ligation independent cloning approach was used to transfer the CBM nucleotide gene sequences from the respective pUC57 to pEC vector (21). For the creation of CBM1 Y31A mutant used for bond rupture assay (reported in **Fig. S14**), site-directed mutagenesis with complementary forward and reverse primers was used. Polymerase chain reaction (PCR) was catalyzed by Herculase II Fusion DNA polymerase (Agilent Technologies, Santa Clara, CA). Destination pEC vector and CBM insert gene amplification was carried out using suitably designed PIPE primer pairs. The PCR amplification of pUC57 and pEC vectors using corresponding vector/insert PIPE reactions primer pairs were carried out in separate tubes. After PCR, respective CBM PIPE reaction product aliquots (2 μL) were mixed together and immediately transformed into competent *E. coli* E. cloni 10G cells (Lucigen, Madison, WI). If the PIPE cloning strategy was not successful, the pUC57 and pEC vectors were digested using *AflII* (New England Biolabs, Ipswich, MA) and *BamHI* (Promega, Madison, WI) restriction enzymes. The restriction enzyme products were ligated using T4 DNA ligase (New England Biolabs) and the ligation mixture was instead transformed into competent *E. cloni* 10G cells. Individual transformant colonies were next screened by PCR amplification and transformants containing inserts with the approximate correct size were identified by agarose gel electrophoresis. Plasmids isolated from positive colonies were sequenced to confirm nucleotide identity at the UW Biotechnology Center (and/or at Genscript, Piscataway, NJ). Transformed strains were stored as 20% glycerol stocks were maintained at -80 ˚C, while all relevant pEC-GFP-CBM plasmids were also maintained at -80 ˚C for long-term storage.

**CBM Family**	**CBM Gene Sequence**	**Organism Source**
1	CCGGGTCCGACCCAGAGCCATTATGGCCAGTGCGGTGGTATTGGTTA TAGCGGTCCGACCGTGTGCGCAAGCGGTACCACCTGCCAGGTGCTG AACCCGTATTATAGCCAGTGCCTG	*Trichoderma reesei*
2a	GCGGCGAGCGGCGCACGTTGCACCGCAAGTTATCAAGTGAATAGCG ATTGGGGGAACGGTTTCACGGTTACCGTCGCAGTTACCAACTCAGGT TCTGTTGCTACCAAAACCTGGACGGTGTCGTGGACCTTCGGCGGTAA TCAGACTATCACCAACAGCTGGAACGCGGCGGTCACACAGAACGGC CAGAGTGTGACTGCACGTAACATGAGCTACAATAATGTTATTCAACC AGGCCAAAATACGACCTTTGGTTTTCAAGCCTCGTACACGGGCAGTA ACGCAGCACCGACCGTTGCGTGCGCGGCGAGT	*Acidothermus cellulolyticus*
2a	Refer to Lim et al. 2014 (20)	*Streptomyces sp. SirexAA-E*

3a	Refer to Whitehead et al. 2017 (19)	*Clostridium thermocellum*
5	ATGGGTGATTGTGCTAACGCAAATGTCTATCCGAACTGGGTGTCTAAAGATTGGGCGGGTGGTCAACCGACGCATAACGAAGCGGGTCAGAGCATTGTGTATAAAGGCAACCTGTACACCGCGAATTGGTACACCGCATCAGTGCCGGGTTCAGACTCATCGTGGACGCAGGTTGGTAGTTGTAATTGA	*Erwinia chrysanthemi*
10	ATGGGCAATCAACAATGTAACTGGTATGGCACCCTGTATCCGCTGTGTGTGACGACGACGAATGGCTGGGGCTGGGAAGATCAACGCAGCTGCATCGCCCGTAGCACCTGCGCGGCTCAACCGGCACCGTTTGGCATCGTGGGTAGCGGCTGA	*Cellvibrio japonicus*
64	CCGACCCCGTCTGGCGAATATACGGCGATTGCCCTGCCGTTTACCTACGATGGCGCCGGTGAATATTACTGGAAAACCGACCAATTCAGCACCGATCCGAATGACTGGTCACGTTATGTCAACTCGTGGAATCTGGATCTGCTGGAAATTAACGGTACCGACTACACGAATGTGTGGGTTGCACAGCATCAAATCACGCCGGCTAGTGATGGCTACTGGTATATTCACTACAAAGGCTCGTATCCGTGGTCGCATGTGGAAATCAAA	*Spirochaeta thermophila DSM 6192*

The sequence-verified plasmid, named pEC-GFP-CBM, encoded a 5' His_8-tag, followed by GFP, a linker sequence, and the relevant CBM. The linker sequence encoded for a 42 amino acid linker peptide reported previously by Takasuka and co-workers (22). Here, we have characterized in detail two distinct but structurally homologous family 2a CBMs from *Streptomyces* sp. SirexAA-E and *Acidothermus cellulolyticus*. *Streptomyces* sp. SirexAA-E was recently identified by GLBRC researchers for its high cellulolytic ability (23). In addition, we have also characterized two distinct but structurally homologous family 1 CBMs from *Trichoderma reesei* belonging to Cel7A (cellobiohydrolase I or CBHI) and Cel6A (cellobiohydrolase II or CBHII) processive cellulases that gave similar results to CBM1 from Cel7A. However, here we exclusively report results for CBM1 from Cel7A. Previous studies have shown that the structure and function relationship of homologous CBMs belonging to the same family are often similar, as also seen in our current work when comparing binding results between the two family 2a CBMs.

Small-scale expression testing of GFP-CBMs: pEC-GFP-CBM plasmids were first transformed into BL21-CodonPlus-RIPL [λDE3] (Stratagene, Santa Clara, CA) or Rosettagami 2 [DE3] (Novagen, Santa Clara, CA) *E. coli* competent strains for small-scale protein induction/expression optimization screening. After cells were grown to the optical density of 0.5-0.7 (mid-exponential phase) in a non-inducing medium (24), expression was induced in either an auto-induction medium (24) at 25 ˚C or in LB medium with varying concentrations of IPTG (0.1-1mM) during incubation at 16 ˚C, 25 ˚C, or 37 ˚C. Apart from GFP-CBM2a (ActE), as also reported previously (20), soluble cytoplasmic protein production was observed for all other GFP-CBMs in nearly all of the expression conditions tested. Superfolder enhanced GFP (or eGFP) tag has been reported to increase soluble fusion protein production yields by likely preventing aggregation of hydrophobic proteins like CBMs (25). Nevertheless, small-scale immobilized metal affinity chromatography with Ni^{2+}-NTA (immobilized metal affinity chromatography or IMAC) purification and cellulose binding assays were conducted for all soluble protein fractions to confirm

cellulose-binding activities and hence identify optimum cell culture conditions for large-scale protein production/purification to run all reported bulk and single-molecule CBM binding assays.

Large-scale expression of GFP-CBMs: *E. coli* BL21-CodonPlus-RIPL [λDE3] (Stratagene, Santa Clara, CA) or Rosettagami 2 [DE3] (Novagen, Santa Clara, CA) competent strains were transformed with the relevant pEC-GFP-CBM plasmid based on the small-scale expression results. Suitable transformants were inoculated into 50 mL of chemically defined non-inducing medium (24), in the presence of 50 μg/mL kanamycin and 25 μg/mL chloramphenicol selection antibiotics. The non-inducing medium contained 2 mM $MgSO_4$, a 1:1000 dilution of trace metal salts mixture (equivalent to 50 mM Fe^{3+}, 20 mM Ca^{2+}, 10 mM Mn^{2+}, 10 mM Zn^{2+}, 2 mM Co^{2+}, 2 mM Cu^{2+}, 2 mM Ni^{2+}, 2 mM Mo^{6+}, 2 mM Se^{4+}, 2 mM H_3BO_3) into the medium, 0.5% glucose, 0.25% aspartate, 50 mM NH_4Cl, 25 mM KH_2PO_4, 25 mM Na_2HPO_4, 5 mM Na_2SO_4, 0.01% methionine, 1% of 17 amino acids (except cysteine, tyrosine, and methionine) each, and a vitamin cocktail (200 nM of vitamin B12, nicotinic acid, pyridoxine, thiamine, p-aminobenzoic acid, and pantothenate; 5 nM folic acid, and riboflavin). The culture was incubated overnight at 25 ˚C and then used to inoculate 2 liters of auto-induction medium (24). The auto-induction medium contained 1.2% tryptone, 2.4% yeast extract, 2.3% KH_2PO_4, 12.5% K_2HPO_4, 0.375% aspartate, 2 mM MgSO4, 0.8% glycerol, 0.015% glucose, and 0.5% α-lactose. The cultures were grown at 25 ˚C for ~27 h. The cells were harvested by centrifugation at 8000xg for 10 minutes at 4˚C and the cell pellet was stored at -80˚C until further use. All chemicals were purchased from Sigma-Aldrich (St. Louis, MO).

Purification of GFP-CBMs: The recovered cell pellet was thawed and re-suspended in 150 mL of ice cold 20 mM phosphate, pH 7.4, containing 500 mM NaCl, 20% v/v glycerol, 10 μg/ml lysozyme, and a protease inhibitor cocktail (containing benzamidine, EDTA and E-64 protease inhibitor from Sigma-Aldrich). The cells were sonicated with an ultrasound sonicator (550 Sonic Dismembrator, Fisher Scientific, Pittsburgh, PA) fitted with a microprobe (1-inch probe diameter) at 4 ˚C for 5 min with 10-s on-bursts and 30-s off periods. The cell debris containing the inclusion bodies was pelleted at 21,000 rpm at 4 ˚C (30 min) and the supernatant was collected in all cases except for GFP-CBM2a (ActE). Details regarding GFP-CBM2a (ActE) expression and purification are provided elsewhere (20). Briefly, due to the insolubility of the expressed GFP-CBM2a (ActE) under all conditions tested, this protein construct was first isolated from inclusion bodies, refolded, and then purified using IMAC as described previously (20). For all other GFP-CBMs, IMAC using Ni^{2+}-NTA based columns/media (GE Healthcare) was first used to isolate and purify His_8-tagged proteins from the *E. coli* cell lysate. All column-based protein purifications were carried out on a ÄKTA-FPLC system (GE Healthcare, Pittsburgh, PA). The cell lysate supernatant was first loaded onto the IMAC column at a medium flow rate of 1 – 2 ml/min. The column was then washed with buffer A (100 mM MOPS, pH 7.4, containing 10 mM imidazole and 100 mM NaCl), followed by additional washing using 95% IMAC buffer A spiked with 5% IMAC buffer B (100 mM MOPS, pH 7.4, containing 500 mM imidazole and 100 mM NaCl), and last followed by elution in 100% IMAC buffer B at a flow rate of 5 ml/min. Protein purity and molecular weight at each stage of the protein purification process was examined by SDS-PAGE (Criterion XT Bis-Tris Precast Gels, Bio-Rad). The presence of partially cleaved GFP-CBMs was identified in the IMAC-B eluents for some protein constructs (namely CBM1, CBM2a, CBM5, CBM10), which necessitated further purification using an amorphous cellulose or hydrophobic interaction affinity-based purification method, as already outlined elsewhere (20, 26), to isolate the intact protein fractions. Briefly, for cellulose affinity-based purification method, IMAC-B protein eluents were directly applied to a phosphoric acid swollen amorphous cellulose (PASC) media at the recommended loading (~200 mg crude protein added per gram dry weight cellulose) for preparative-scale purification (26). The amorphous cellulose slurry was prepared ahead of time and preequilibrated in a 50 mM pH 6.5 MES buffer (equilibration buffer or buffer A) at the desired solids concentration (10 g/L), prior to addition of the IMAC-B protein eluent. The crude protein-cellulose slurry was then intermittently and gently mixed at room temperature for a total incubation time of 0.5 h. The protein bound to PASC was then separated from the unbound protein in the supernatant by gentle centrifugation at 3500xg for 10 min at 25˚C. The recovered PASC pellet was then resuspended in a wash buffer (i.e., equilibration buffer+1M NaCl), using a 4:1 buffer to PASC pellet ratio (v/v), and gently mixed at room temperature for 10 mins to remove non-specifically bound proteins. The recovered PASC pellet containing the adsorbed GFP-CBMs was then finally suspended in

100% ethylene glycol elution solution, using a 4:1 glycol to PASC pellet ratio (v/v). The final ethylene glycol concentration of ~80% (v/v) was sufficient to elute a significant fraction of reversibly bound GFP-CBMs into the supernatant. The eluted protein rich supernatant was separated from PASC pellet and stored in 80% glycol solution at −20 ˚C for short term storage or immediately concentrated using IMAC columns prior to buffer exchange into 10 mM pH 6.5 MES (or pH 5.5) buffer and storage at −80 ˚C for long term storage in 0.5-1 ml aliquots. The molecular weight of the intact purified GFP-CBM monomers was confirmed by SDS-PAGE to match with the predicted translation products. Protein concentrations were estimated spectrophotometrically at 280 nm using the extinction coefficients calculated from the amino acid sequences for each construct. The histidine tags were not removed and have been reported to not influence CBM binding to cellulose (20, 27).

GFP-CBM 'pull-down' binding assays with cellulose allomorphs: Cladophora based cellulose allomorphs, prepared as described above, were used to estimate binding affinity and partition coefficients for GFP-CBMs. All cellulose binding assays were carried out in 2 mL microcentrifuge tubes using 5 mg of pre-weighed cellulose suspended in 500 μL of 10 mM MES buffer, at pH 5.5 (for CBM1) or 6.5 (for all other CBMs), containing 2.5 mg/mL of bovine serum albumin (BSA) to minimize non-productive protein binding to the tube wall and/or denaturation at the air-buffer interface during mixing. Purified GFP-CBMs aliquots were thawed and buffer-exchanged (PD-10 desalting column, GE Healthcare) into 10 mM MES, at desired pH, and added to each 2 mL tube to achieve a final solution concentration. The final concentration typically ranged from 1-500 μg/mL for estimating partition coefficients or from 1 μg/mL to 10 mg/mL for full scale binding affinity measurements. The tubes were mixed in an orbital mixer set at 1000 rpm (ThermoMixer, Eppendorf) at 25˚C for 1.5-2 h to allow binding equilibrium to be reached. Controls with only proteins but no substrate were mixed to track total added protein concentration, while never-mixed controls with only proteins/buffer were used as controls to account for possible protein loss during mixing. Insoluble cellulose was recovered by centrifugation at 17000xg for 2 min at 25˚C and 200 μL of the supernatant (w/wo suitable dilution in identical buffer) containing unbound protein was assayed for GFP fluorescence (488 nm excitation and 509 nm emission; with 495 nm cut-off) to quantify the total fraction of bound protein versus the original added amount (2). All assays were performed in at least duplicate for each protein loading condition (~22 protein loadings). Error bars reported for all bulk binding assays represent one standard deviation (±1σ) from mean values of replicates.

Calcofluor White dye 'pull-down' binding assays with cellulose allomorphs: Calcofluor White dye (Sigma-Aldrich) binding assays on Avicel PH-101 based cellulose I and III were performed in 1.5 ml Eppendorf tubes at varying dye concentrations, similar to previously reported cellulose-solute binding assay methods (2, 17). Briefly, 5 mg of Avicel cellulose-I or cellulose-III was added to each well by pipetting suitable aliquots of a uniformly suspended cellulose slurry prepared in deionized water. Suitable calcofluor dilutions were prepared in deionized water (at pH 7.0) to achieve the desired concentration in each tube. Note that 30 mM NaCl was added to each tube. For cellulose blanks, deionized water alone instead of cellulose was added to make up the volume instead to the final desired 620 μl level. All binding assays were performed with three replicates per assay condition. The tubes were then shaken in Thermomixer at 1000 rpm for 1 h at room temperature. After 1 hour, all tubes were centrifuged at 17000xg for 5 minutes at 25˚C and supernatant was removed from each well to be transferred into opaque microplates for reading calcofluor fluorescence. The plates were read using a Molecular Devices M5e spectrophotometer at the following settings: 365 nm excitation, 450 nm emission. Langmuir based models were also fitted to the binding dataset as described below. Error bars reported for all binding assays represent one standard deviation (±1σ) from mean values from three replicates.

Langmuir-type adsorption model fitting to pull-down adsorption assay data: The bound (μmol protein/g cellulose) and free (μM) protein concentrations from the binding assays were fit to a Langmuir single-site, two-site, and Langmuir-Freundlich adsorption models to determine maximum binding capacity ($n_{\max}$) and equilibrium dissociation constant (K_{d}) for each cellulose allomorph and GFP-CBM combination. The model equations are displayed in **Figure 4B**. The linear range of the binding curve was used to estimate the partition coefficient (α) as described previously (20). The data from pull-down binding assays is processed to yield free protein and bound

protein concentrations (or calcofluor concentrations) in μM and μmol/g cellulose respectively in Excel™. This data set was then exported into OriginPro 2019. OriginPro software already has several non-linear curve fitting options available. However, separate functions were created for Langmuir single-site, two-site, and Langmuir-Freundlich models using the custom function builder tool. Similar Langmuir-type models have been used previously for Cel7A-cellulose binding analysis (2, 17). These functions were then used to fit binding assay data with the following settings for curve fitting: Levenberg-Marquardt algorithm with a tolerance of $1e^{-9}$. If the fitting analysis did not converge in Origin for more complex models such as Langmuir two-site and Freundlich models, Excel Solver™ was used for curve fitting in those cases. The sum of errors was specified as objective function and parameters were estimated using the GRG non-linear method. Parameter uncertainties were then calculated using an elaborate process involving Monte Carlo simulations as detailed elsewhere (28).

Reversible binding of GFP-CBMs to cellulose*:* The application of Langmuir-type adsorption models is dependent on reversible binding of protein to substrate. Hence, reversible binding properties of the GFP-CBMs to Cladophora cellulose I and cellulose III, was determined as described previously (20, 29). This approach was used to confirm the reversible binding of GFP-CBM1 and GFP-CBM3a to both Cladophora cellulose allomorphs in the pull-down binding assay setup (data not shown). Briefly, after the binding equilibrium was re-established after dilution of the mixture, the newly estimated bound/free protein concentration was confirmed to lie along the adsorption isotherm curve as already shown by us previously for CBM2a (ActE) (20). Since the partition coefficient was determined regardless of the dilution ratio, this result confirmed that the protein showed indeed reversible adsorption to cellulose. The presence of excess BSA prevented non-specific binding of CBMs to various surfaces like plastic tube walls (30), which along with reduced possible GFP-CBM denaturation at air-liquid interfaces during extensive mixing at low protein concentrations due to presence of sacrificial BSA (31), also minimized bias in reversibility binding measurements of GFP-CBMs particularly to cellulose III.

Molecular dynamics simulation of CBM1 and cellulose allomorphs interactions: All molecular dynamics (MD) simulations were conducted using the Amber force field for CBM1 protein (1cbh pdb code) (32), TIP3P explicit water model (33), and the Glycam force field for representing the cellulose fiber (34). As described previously (35), X-ray and neutron diffraction coordinates were used to generate one rhomboid cellulose Iβ fibril and one rhomboid cellulose III fibril (36, 37). Each fibril consisted of 36 glucan chains each. Two different types of crystalline cellulose III were used for the simulations, one with lower crystallinity and another with high crystallinity (based on the available crystalline core cellulose crystal structure). However, since no detailed structural information is available for lower crystallinity cellulose III (3), an idealized model structure was obtained by increasing the simulation temperature to 400 K starting with the high crystallinity cellulose III structure to increase overall disorder within the crystalline fiber. The cellulose Iβ rhomboid shape was chosen as the base case control because of its wide hydrophobic surfaces accessibility which has shown to be preferred binding site for Type-A CBMs like CBM1 (38, 39). Furthermore, this hydrophobic binding surface plane of cellulose Iβ is also identical to cellulose Iα (40), from a crystallographic point of view, which would aid in drawing similar conclusions when assessing CBM1 binding to hydrophobic surfaces of native cellulose I fibrils from Cladophora, which is mostly enriched in cellulose Iα unlike cellulose I fibrils from Avicel derived cellulose nanocrystals that are enriched in cellulose Iβ.

Two types of simulations were run: (a) First, unbiased simulations were used to probe the binding dynamics of CBM1 starting from the hydrophobic surfaces of cellulose I and III microfibrils on microsecond timescales. MD simulations were conducted in order to populate the most preferred orientations of CBM1 on either cellulose allomorph surface. CBM1 was first aligned in the canonical direction for the reducing end specific action of Cel7A to processively hydrolyze cellulose chain (i.e., the Y31 residue end of CBM1 pointing towards the nonreducing end of cellulose). If no stable binding was seen, as was the case with cellulose III, the CBM1 was aligned in the most stable conformation identified in the unbiased simulations (i.e., Y31 residue end of CBM1 pointing towards the reducing end of cellulose). From these unbiased simulations, we were able to calculate the distribution frequency of the most preferred rotameric states for the closely cellulose-contacting aromatic residues in CBM1. (b) Second, the preferred orientation inferred from unbiased simulations for CBM1 on cellulose I and III allomorphs were used

in order to estimate their respective association free energy with the cellulose fiber. Thus, a potential of mean force (PMF) was calculated to estimate the CBM1 binding free energy during adsorption to the hydrophobic surface of all cellulose allomorphs. We used the coordinates of the last frame from the unbiased CBM1-cellulose systems at 310 K. A total of 14 independent windows per system were used, which were spaced apart by 1 Å. A restraining potential of 1000 kJ mol^{-1} nm^{-2} was applied to the center of mass (COM) of CBM1 with respect of the COM of either cellulose I or cellulose III along the normal (z) coordinate. For each window, 1.5 µs long simulations were performed. The desorption free energy was reconstructed using the weighted histogram approach and convergence was assessed via block averaging by dividing the trajectory in three independent blocks. Umbrella sampling-based simulations were conducted along a path where the CBM1 translated from the hydrophobic surface of the microfibril to the bulk solvent. The position of the potential changes every 1 Å along the pulling reaction coordinate (in this case the z vector). Harmonic potential applied to the center of mass of the protein. Along the trajectory, the two most populated configurations are shown here in the main text figure, together with the protein residues in close contact with the external cellulose surface. These configurations also correspond to the minimum wells seen in the energy plots.

All simulations were performed using a 2-fs time step using GROMACS software. The LINCS algorithm was applied to constrain all bond lengths with a relative geometric tolerance of 10^{-4}. Non-bonded interactions were handled using a twin-range cutoff scheme. Within a short-range cutoff of 0.9 nm, the interactions were evaluated every time step based on a pair list recalculated every five-time steps. The intermediate-range interactions up to a long-range cutoff radius of 1.4 nm were evaluated simultaneously with each pair list update and were assumed constant in between. A PME method was used to account for electrostatic interactions with a grid spacing set to 0.15 nm. During the equilibration (0.1 µs), systems were coupled using a Berendsen barostat to 1.0 bar via an isotropic pressure approach, with relaxation time of 1.0 ps. Afterwards, system was coupled to a Parrinello barostat algorithm and constant temperature was maintained by weak coupling of the solvent and solute separately to a velocity-rescaling scheme with a relaxation time of 1.0 ps.

Preparation of Avicel cellulose nanocrystals through acid hydrolysis for QCM-D: Avicel cellulose III (or CIII) was prepared from Avicel PH-101 (also referred to as Avicel cellulose-I (or CI) (Sigma Aldrich Lot#BCBG9043V) as described previously (3). Nanocrystals were prepared from both Avicel cellulose-I and Avicel cellulose III using the same procedure. Briefly, 2 g of Avicel was added to 70 ml 4 N HCl in a glass beaker and placed in a pre-heated water bath at a temperature of 80° C. The slurry was stirred every half hour using a spatula to ensure cellulose is well suspended. After 4 hours of reaction time, the acid hydrolysis mixture was diluted with 50 ml deionized water. The slurry was then aliquoted into 50 ml centrifuge tubes, with 40 ml slurry in each tube and centrifuged at 1600xg for 10 minutes at 25 ^{0}C. The supernatant was decanted, and the cellulose pellet was washed with 10 ml deionized water. The wash steps were repeated, and the supernatants were discarded until they turned hazy around pH 3.3. The haziness of supernatant indicates evolution of cellulose nanocrystals and hence these supernatants were collected into a separate bottle for future usage.

Preparation of cellulose thin films for QCM-D: This procedure was developed based on similar previous studies investigating cellulose hydrolysis and binding of cellulases using QCM-D (41). Briefly, 4.95 MHz quartz crystal sensors (0.55" diameter), with SiO_2 coating were purchased from Filtech (product code QSX0303). The sensors were first rinsed in water, followed by ethanol and then blow-dried. The sensors were then immersed in 0.02% PDADDMAC (Sigma Aldrich 409022), which serves as an anchoring layer for cellulose, for 1 hour at 25°C with orbital mixing. This was followed by washing with deionized water for 1 hour with orbital mixing at 25°C. The sensors were then blow-dried and spin-coated using a pre-cycle spin for 3 s at 1500 rpm, followed by a spin cycle for 60 s at 3000 rpm. This spin coating step was repeated 10-20 times to obtain a uniform cellulose film thickness of ~20 nm (as measured using the QSoft software using Sauerbrey model). In a previous study (41), a lesser number of spin-coating steps was used to achieve the same thickness, however, this discrepancy may be related to the nanocrystal slurry concentration and hence needs to be optimized accordingly.

Fluorescence recovery after photobleaching (FRAP) to study CBM-cellulose binding kinetics: We assembled a simple fluidic chamber using a glass coverslip (24 mm X 30 mm, No. 1.5, Thermo Scientific) and a holed glass slide (470150-480, Ward's Science) that was pre-cleaned with acetone (A949, Fisher chemical), methanol (A454, Fisher chemical), and distilled water. To improve the cellulose sample adhesion, the glass substrates were treated with plasma (PDC-001, Harrick Plasma) for 10 minutes. A drop of 20 µL cellulose solution was deposited on a coverslip, which was dried in a hybridization oven (VWR) at 50°C overnight. A sample chamber was assembled by attaching the coverslip to a glass slide with multiple pieces of double-sided 3M tape; the gap between the edges of two adjacent tapes formed a channel. The open edges were sealed with 5 minutes epoxy glue (Devcon). Next, 100 µL of 5 µM GFP-CBM3a buffered solution (10 mM MES pH 5.5; no BSA included) was finally injected into the sample chamber through the holes on the glass slide, which then was incubated for 10 minutes before running FRAP experiment.

For FRAP experiment, we used a Total Internal Reflection Fluorescence (TIRF) microscope, which was homebuilt with an inverted microscope (Ti-E, Nikon), high NA objective lens (CFI-apo 100X, NA 1.49, Nikon), 488 nm laser (Coherent) and EMCCD camera (iXon Ultra-888, Andor) (42). We scanned through the cellulose sample to find an area to be imaged and acquired a reference wide-field fluorescence image at a low 488 nm excitation power (2-3 mW) prior to photobleaching. Guided by the pre-bleach image, we decided on multiple locations to be photobleached, and created focused laser spots at the corresponding positions using a spatial light modulator (X13138-01, Hamamatsu). Typically, a maximum of 25 spots on the cellulose fibers along with 2 spots on the background area were chosen. We locally photobleached the selected spots with illumination of a strong 488 nm laser power (50-120 mW) for 1 minute, and then time-lapse wide-field fluorescence images were acquired for 40 minutes to monitor fluorescence recovery. Focus drift was actively compensated with Perfect Focus System (Nikon) while XY-drift was corrected using ImageJ software (NIH).

Custom written MATLAB scripts were employed for the extraction of the recovery curves and data analysis. The photobleached segments were automatically identified by subtracting pre-bleach from post-bleach images. The recovery curves were corrected for non-specific GFP-CBM binding to the glass slide within a segment by setting all pixel values below a segment-specific threshold to zero. The average pixel intensity value of each segment before photobleaching was used to normalize the recovery curve. Additionally, the FRAP signal was baselined to zero relative intensity based on the first FRAP data point to account for the non-zero dark current of the camera and inhomogeneous illumination across the FOV as well as inefficient photobleaching. Each FRAP curve was fitted to the model given in equation below developed by Moran-Mirabal (43) using a Levenberg–Marquardt curve fitting approach built-in MATLAB. The baseline correction is then added to $F_{M,fit}$ to obtain the true F_M value.

$$I(t) = F_M * \left(1 - e^{k_{\text{off}} * t}\right)$$

where

$$F_M = \frac{k_{\text{on}}^* C_F \alpha}{k_{\text{off}}}$$

With $I(t)$ being the normalized intensity value at time t, F_M being the fraction of reversibly bound GFP-CBM3a, k_{off} the desorption rate constant for GFP-CBM3a, k_{on}^* the pseudo-adsorption rate constant assuming a first order binding reaction, C_F the concentration of protein in solution and α the slope of calibration curve between fluorescence intensity and protein concentration. Fits with an $R^2 \leq 0.85$, $F_M \leq 0$ or $F_M \geq 1$ were excluded from further analysis. The collection of fit parameters for cellulose allomorph (F_M, k_{off}) were then fitted to a Gaussian distribution to extract the mean and standard deviation of each parameter. Under the assumption that C_F and α are similar in the case of cellulose I (CI) and cellulose III (CIII), the above equation can be reduced to the following:

$$\frac{F_{M,CI}}{F_{M,CIII}} = \frac{k_{\text{on,CI}}^*}{k_{\text{on,CIII}}^*} \times \frac{k_{\text{off,CIII}}}{k_{\text{off,CI}}}$$

This equation can then be used to infer the ratio of $k^*_{\text{on,CI}}$ and $k^*_{\text{on,CIII}}$ and hence comment on how binding rate constant on one allomorph compared to another (see ref. (43) for a detailed overview of this model equation).

Quartz crystal microbalance with dissipation (QCM-D) based CBM-cellulose binding assay and data analysis: Binding assays were performed using QSense E4 instrument (NanoScience Instruments). Quartz sensors with cellulose thin films were mounted and equilibrated with buffer (10 mM MES pH 6.5; no BSA included) at a flow rate of 100 µl/min for 10 minutes using a peristaltic pump. The flow of buffer was then stopped, and the cellulose films were left to swell overnight in buffer. The frequency and dissipation changes were tracked for all harmonics and the cellulose films were considered amenable to binding studies if the third harmonic stabilized after overnight equilibration in the buffer. Proteins of interest (i.e., GFP-CBM3a) were diluted to a concentration of 1 µM beforehand and flown over the sensors at a flow rate of 100 µl/min for 10 minutes. All proteins tested, attained saturation within 10 minutes as noticed from frequency and dissipation traces. The CBM-cellulose system was left to equilibrate for at least 30 minutes. Unbinding of proteins was tracked by flowing 10 mM MES pH 6.5 buffer at 100 µl/min for at least 30 minutes. The sensors were finally treated with 5% contrad solution followed by deionized water at 100 µl/min for 10 minutes, to remove any traces of protein left in the tubing. The frequency data was converted to mass deposited on sensor using Sauerbrey equation, which was in turn converted to the number of molecules of protein deposited on cellulose surface. The QCM data was then used to obtain pseudo-association rate constant (k^*_{on}) and dissociation rate constant (k_{off}) using the equations provided below:

<u>Binding rate equation:</u>

$$[EC] = A(1 - e^{-(k^*_{\text{on}})t})$$

<u>Unbinding rate equation:</u>

$$[EC] = Ae^{-(k_{\text{off}})t}$$

Where $[EC]$ = Number of molecules; t = Time (minutes)

Buffon needle model analysis of CBM orientation probability distribution on cellulose surface: Buffon's needle model arose from a problem first posed by the eponymous French mathematician Buffon (44, 45). The original model is an analytical solution to the question of the probability of a short needle crossing the parallel lines on a ruled paper or wooden floor. An analytical problem to this solution does exist and the numerical solution to this problem would involve the Monte Carlo method. Here, we have adopted the Monte Carlo approach to simulate the CBM-cellulose system with some simplifications which render the binding process completely from a simply geometric probability point-of-view and without any energetic constraints taken into consideration. The CBM was assumed to be a needle with length of 2.08 nm representing the distance between centers of flanking planar Tyrosine rings (Y5 and Y31) based on the published NMR structure (PDB ID: 1cbh) (see **Fig. S16A**). Cellulose-I binding surface was assumed to be an array of parallel lines, each line representing a cellulose chain, with an inter-chain distance of 0.8 nm (see **Fig. S16B**). In the energetically favorable configuration of this system as predicted by MD simulations, CBM1 aligns with two aromatic residues (Y31, Y32) on one cellulose chain and another aromatic residue (Y5) on an adjoining cellulose chain. Hence, any event where the CBM needle lands on a chain perfectly (C_0), crosses only one chain (C_1) were clustered into a category called events 'along the chain'. Similarly, events where the CBM needle crosses two chains (C_2) and three chains (C_3) were clustered into a category called events 'across the chain'. The configuration of this system at any time can be defined by the angle Θ between the cellulose chains and the CBM needle. A Monte Carlo simulation was then conducted with 100,000 trials whereby a random number generator was used to sample the angle Θ (see **Fig. S16C-D**). Configuration from a given trial can then be classified into one of the five categories mentioned above ($C_0 – C_3$) and eventually, the probability of CBM crossing two chains or more. See **Fig. S16** for additional methodology related details. The fraction of events along and across the chain are computed based on the following formulae.

$$Fraction\ of\ events\ along\ the\ chain = \frac{C_0 + C_1}{C_0 + C_1 + C_2 + C_3}$$

$$Fraction\ of\ events\ across\ the\ chain = \frac{C_2 + C_3}{C_0 + C_1 + C_2 + C_3}$$

SI Appendix Results and Discussion:

AFM imaging of Cladophora cellulose allomorphs: Previous studies on cellulase or CBM binding have often employed two-site binding models (46), with the assumption of two distinct classes of binding sites arising from any of the following phenomena: (i) binding to amorphous/crystalline regions or (ii) binding to two different faces of crystalline cellulose fiber, and/or (iii) due to different orientations of CBM on hydrophobic face of cellulose. Since Cladophora derived cellulose is a highly crystalline substrate and Type-A CBMs bind to crystalline regions predominantly, a small number of high affinity amorphous binding sites seems unlikely. However, the availability of different crystal faces for binding to non-native allomorph cellulose III needed to be examined and hence we performed AFM imaging on both Cladophora cellulose I and cellulose III microfibrils to verify the general morphological differences between two substrates (see **Fig. S1**). AFM imaging indeed suggests that the Cladophora cellulose I elementary fibrils have two distinct crystalline planar surfaces likely available for CBM binding unlike cellulose III, consistent with predicted crystal structure of algal cellulose and morphological changes undertaken following ammonia treatment (37, 40, 47). This change in cellulose I fibril shape is expected to take place during ammonia pretreatment due to a solid-state polymorphic transformation that alters the cellulose III unit cell crystal structure (37, 48–50).

Truncation and sensitivity analysis for CBM1 binding data set: We varied the free protein concentrations over nearly four orders of magnitude (0.02-250 μM for CBM1 and 0.02-50 μM for CBM3a) to accurately capture binding interactions at both low (<10-fold of min K_d) and high (>10-fold of max K_d) protein-to-substrate saturation concentrations. This broad range of protein concentrations is often recommended to properly characterize CBM adsorption to polysaccharides (51), but is not always followed by researchers, which could explain some of the discrepancy in the literature for reported $n_\mathrm{max}/K_\mathrm{d}$ values for various CBMs. Langmuir two-site models are often interpreted as consisting of two different classes of binding sites (46), one with high affinity and another with low affinity (a higher value of K_d corresponds to a lower affinity). Langmuir-Freundlich model, on the other hand, also provides information regarding the binding cooperativity for a given substrate (52). However, since most studies do not validate all model assumptions, it is very challenging to draw any molecular-level understanding of protein adsorption process using such traditional approaches alone. Furthermore, since the root-mean square errors from the model fits to the experimental data are nearly comparable in most cases, it is difficult to choose an appropriate model based on goodness of model fit without any observer bias. Details regarding sensitivity of results to truncation of original CBM1 data set is discussed already in the main manuscript (see **Table 2**). Furthermore, we also performed sensitivity analysis for the original CBM1 to cellulose I binding data set by studying the variation in RMSE when the binding parameters ($n_\mathrm{max}, K_\mathrm{d}$) are varied individually by 10% and 20% each (see **Table S2**). This sensitivity analysis revealed that the n_max parameter is quite sensitive to error in data acquired at higher protein concentrations, emphasizing the need for enough replicates while probing higher protein concentrations.

MD simulations reveal improper stacking interactions of aromatic residues with cellulose III: MD simulations were conducted to identify the preferred conformations of CBM1 on each cellulose allomorph surface and estimate the theoretical binding affinity of CBM1 for the most preferred orientation. Two different types of crystalline cellulose III models were used for the simulations, one with lower crystallinity and another with high crystallinity (based on the available crystalline core cellulose crystal structure). A recent study (3) has shown that CBM3a binding to cellulose-III closely depends on the cellulose III fibril crystallinity. Therefore, we were interested in modeling the binding of CBM1 to two types of cellulose III crystals with varying degree of crystallinity for this study. However, since no detailed structural information is available for lower crystallinity cellulose III, a modeled structure was obtained by increasing the simulation temperature to 400 K starting with the high crystallinity cellulose III structure to increase overall disorder within the crystalline fiber. Previous studies employing MD simulations (39) and CBM-cellulose EM-immunolabeling experiments (53) have already shown that the preferred face for binding of CBM1 to cellulose I is the hydrophobic face. Therefore, the hydrophobic faces of cellulose I and cellulose III were initially chosen to study the binding preferences of CBM1 in this study as well.

As shown in **Fig. S5A**, the unbiased MD simulations revealed that the most populated configurations for CBM1 on cellulose I have the CBM1 Y31 residue facing towards the non-reducing end of the cellulose chain (as also reported previously (38)). This chain-end preference could have evolved from the reducing end specificity of the Cel7A catalytic domain to which the native CBM1 is attached at the C-terminus. Furthermore, it was also observed that CBM1 remained stably bound to cellulose I surface regardless of whether the Y31 residue was facing the reducing or non-reducing end. Interestingly, CBM1 was stably bound to the cellulose III surface (for both low and high crystallinity forms of cellulose III) only when the Y31 residue was facing the reducing end of cellulose. See **Movies S1-S3** for representative visualization of CBM1-cellulose allomorphs interactions from unbiased MD simulations. The relative diffusivity of CBM1 and the RMSF for key aromatic residues is provided here as well (see **Fig. S5B-C**). The improper stacking of Y5 residue on cellulose III specifically is highlighted in **Fig. S5D**.

CBM3a exhibits increased desorption rate constant towards cellulose III: In addition to equilibrium binding assay methods, we used FRAP to study GFP-CBM3a binding to Cladophora derived cellulose allomorphs (with native cellulose enriched in Iα) and QCM-D to study GFP-CBM3a binding to plant-derived Avicel microcrystalline cellulose allomorphs (with native cellulose enriched in Iβ) (54) to obtain a more comprehensive picture of CBM3a binding kinetics towards various cellulose allomorphs.

Procedures for preparation of cellulose nanocrystals, nanocellulose thin film deposition, QCM-D binding assay, and data analysis procedures are outlined in the SI appendix materials and methods section. Sauerbrey equation was used to obtain the mass of adsorbed protein on cellulose film using the frequency change at third overtone (55). The number of protein molecules was then estimated from the mass of adsorbed protein and this resulted in QCM sensorgrams as shown in **Fig. S10A-B.** This data was then analyzed using exponential fitting routines (see exponential fits to acquired QCM raw data in **Fig. S10C-D**), to obtain a pseudo association rate constant (k_{on}^{*}) and dissociation rate constant (k_{off}). While k_{on}^{*} did not show a significant difference between the two allomorphs, CBM3a gave a nearly 3-fold increase in $k_{\mathrm{off}}$for cellulose III (**Table 4**). In addition, the maximum amount of protein bound to cellulose III was 1.5-fold less. These observations align well with the reduction in binding of CBM3a to cellulose III observed using the classical pull-down binding assay method. A similar reduction in CBM binding was observed to another non-native allomorph (i.e., cellulose-II) using QCM-D (56).

Next, we performed FRAP experiments, with details regarding the assay method and data analysis outlined in the SI appendix experimental procedures section. Briefly, similar to previous work (43, 46), GFP-CBM3a binding kinetic parameters to cellulose allomorphs were obtained by fitting the FRAP curves to a binding-dominated model ignoring any diffusion relevant contributions (see supplementary information **SI Appendix Fig. S9** for representative FRAP images and recovery traces). Our FRAP analysis revealed that CBM3a gave a 1.9-fold increase in k_{off} for cellulose III compared to cellulose I (**Figure 6**). F_M and k_{off} were together used to extrapolate the ratio of adsorption rate constants for cellulose I and cellulose III (see **Experimental Procedures** for model equations) which was calculated to be 0.64 $\pm$ 0.29. Hence, the difference in adsorption rate constants between the two allomorphs could only be marginal whereas the desorption rate constant alone was deduced to be significantly lower for cellulose III. In summary, solid state depletion equilibrium binding assays as well as both QCM and FRAP binding assays together provide complementary information on the differences in binding affinity and kinetic rate constants for CBM binding to cellulose allomorphs. However, neither of these methods alone provide a detailed molecular-level understanding of Type-A CBM binding, particularly for weaker binders like CBM1 that impact cellulase binding and activity towards distinct allomorphs.

Reduced partition coefficients of Type-A CBMs towards cellulose III: Since it was challenging to pick a suitable Langmuir model to identify a clear molecular-basis for reduced CBM1 or CBM3a binding to cellulose III, we also estimated the partition coefficients (i.e., $n_{\mathrm{max}}/K_{\mathrm{d}}$) for a larger library of several Type-A CBMs for both cellulose allomorphs at room temperature under non-saturating protein loadings (raw data provided in SI Appendix **Fig. S7** and **Fig. S8** for bar graph). The relative binding order of CBM families based on estimated partition coefficient

value was roughly similar between both allomorphs, where Family 1~Family 2a << Family 64 < Family 3a < Family 5~Family 10. However, the partition coefficients for all Type-A CBMs tested were always significantly lower for cellulose III. The decrease in partition coefficients ranged from 2-fold to 13-fold depending on the CBM family, with CBM64 and CBM1 showing the largest (~13.3-fold) and smallest (~2.3-fold) fold change between the two allomorphs, respectively. Note that the GFP domain alone had insignificant binding affinity towards either cellulose allomorph (~0.1 L/g partition coefficient), suggesting that the CBM domains alone were largely responsible for binding of GFP-CBM fusion proteins to either cellulose allomorph.

Reduced calcofluor dye adsorption to cellulose allomorphs: Calcofluor is a trans-stilbene based fluorescent dye that has a low molecular weight (917 g/mol) and a core structure analogous to the planar interface of Type-A CBMs involving both aromatic and polar functional groups that provides the thermodynamic driving force for binding to complementary cellulose surfaces. Calcofluor is thus an ideal inorganic CBM-like surrogate probe useful for understanding the multivalent binding interactions. Interestingly, Calcofluor dye was also found to show reduced partition coefficient towards microcrystalline cellulose III by 2.6-fold versus native cellulose I (see **Fig. S15A**). Calcofluor is expected to bind cellulose with high affinity, based on reports on how it impacts *in vivo* cellulose synthesis (57) and histological staining analysis of cell wall polysaccharides (58). However, to the best of our knowledge, a direct measurement of calcofluor affinity to microcrystalline cellulose has not been reported. Based on our two site Langmuir model fitting analysis, we found that calcofluor white dye binds to microcrystalline cellulose I with a lower K_{d1} of 38.8 μM to 81% of total available sites and K_{d2} of 1.2 μM to the remaining 19% of total available sites ($n_{\max}$ total was 21.5 μmol/g cellulose). For cellulose III, we found that calcofluor white dye binds with a higher K_{d1} of 79.3 μM to 70% of total available sites and K_{d2} of 1.8 μM to the remaining 30% of total available sites ($n_{\max}$ total was 9.2 μmol/g cellulose). Based on single site model fitting analysis, we found that calcofluor white dye binds to microcrystalline cellulose I with a K_{d} of 18±3 μM and $n_{\max}$ of 19.9±1.0 μmol/g cellulose, while for cellulose III a K_{d} of 15±4.5 μM and $n_{\max}$ of 7.7±0.5 μmol/g was estimated. However, the two-site model gave a much better fit for the data unlike the one-site model. Three-site model gave no further improvement to model fitness for either cellulose I or III. While, these values are likely dependent on the buffer ionic strength conditions, calcofluor has lower affinity and binding sites available for adsorption to microcrystalline cellulose III versus native cellulose I. It is known that strong non-covalent interaction forces stabilize interaction of calcofluor along the repeating ~1 nm cellobiosyl-unit of cellulose along the chain axis (58). This could explain why calcofluor dye binds in a well-defined orientation parallel along the fiber axis to both chitin and cellulose (59). However, altering the crystal structure of native cellulose I to cellulose III could destabilize calcofluor binding due to steric clashes with adjacent cellulose chains due to changed crystal morphology and/or increased fibril hydrophilicity. These findings shed further light into the thermodynamic mechanism driving reduced binding interactions of critical planar CBM binding surface associated amino acid residues to cellulose III. Interestingly, as reported previously for CBM1 binding to crystalline cellulose (52), Scatchard plot analysis for calcofluor binding to cellulose was also non-linear and concave-upward (see SI appendix **Fig. S15B**). Calcofluor dye binding to multiple classes of non-equivalent binding sites could provide a classical interpretation of concave-upward Scatchard plots (60). Nevertheless, when characterizing bulk ensemble binding interactions to highly heterogenous crystalline cellulose microfibril surfaces, even for simple CBM-analogues like calcofluor, our work brings to the light the severe challenges associated with choice of multi-site adsorption models and possible misinterpretation of results.

Buffon needle model for predicting CBM1 orientations: The model formulation and Monte Carlo simulation procedure used here are described in detail in **SI Appendix Experimental Procedures** section. Our simple Buffon needle model predicted that the geometric probability of a CBM1 wild type 'needle' to bind along a single cellulose chain is ~42%, while the remaining ~58% of events would include binding across multiple cellulose chains (ignoring any energetic barriers to binding orientation) (see **Table S3**). Interestingly, if a mutation on CBM1 (Y31A for instance) is considered as having reduced needle length, that would increase the percentage of events along the chain to 90%. Hence, performing these planar aromatic residue mutations and testing the impact of these mutations on the heterogeneity of CBM binding to cellulose studied using bond rupture assays could give us some insight into

the role played by CBM binding orientations on observed heterogeneity. Preliminary data from our CBM-cellulose bond rupture assay indeed shows that there are some distinct differences in the force-lifetime plots for CBM1 wild-type and Y31A mutant on cellulose I (e.g., see 0-2.5 pN and 10-15 pN ranges in **Fig. S14**). However, it needs to be emphasized that this is an over-simplistic model and an understanding of the energetic constraints could better simulate the complex reality of CBM-cellulose interactions. Moreover, we currently lack the ability to theoretically relate this simple model's predictions back directly to the bond rupture assay results. Most previous CBM binding focused studies (38, 39, 61) have not emphasized the possible orientations of CBM1 on the surface of cellulose I. Beckham *et al.* (38) previously showed that although CBM1 prefers to bind along the cellulose chain as well, slightly rotated (by ~10-15°) CBM1 orientations across multiple cellulose chains are energetically feasible as well on individual fiber surfaces. More detailed MD simulations and corresponding rupture assays need to be conducted to check how mutations of CBMs impact along the chain axis versus across multiple chain axis binding and its potential impact on non-productive processive cellulase binding.

SI Appendix Figures & Tables

Fig. S1. AFM imaging of Cladophora-derived cellulose I or CI (A, D) and cellulose III or CIII (B, E) elementary fibrils, the scale bar is 500nm. The black line in panel A and B corresponds to the height signal (forward and backward) shown in panel D and E. As is can be seen in panel D and E, there is no difference in the forward and backward signals, hence only the forward signal was used to analyze the height profile dimensions which are depicted in panel C. Cellulose I fiber showed a peak with a clear shoulder while no such shoulder was seen for the cellulose III fibril, which is consistent with the modification in fiber shape after ammonia pretreatment.

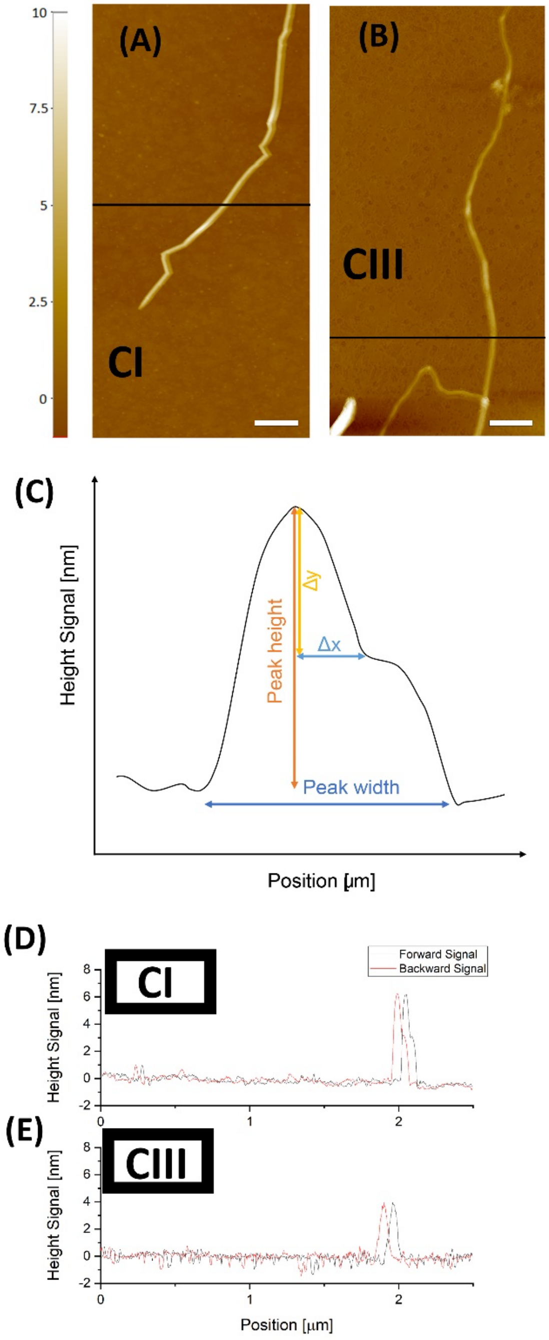

Fig. S2. Single-molecule optical tweezers-based verification of binding stability and instability for full length Cel7A cellulases processively hydrolyzing Cladophora based cellulose I and III. Three representative traces each are shown for the conditions of stability on cellulose I (red), stability on cellulose III (blue) and instability on cellulose III (black). Cel7A was found to be more often stably bound to cellulose I than to cellulose III before initiating stable processive motility accordingly. Of the traces showing instability, Cel7A was likely to have more rupture events during initiation of the processive catalytic cycle on cellulose III than on cellulose I.

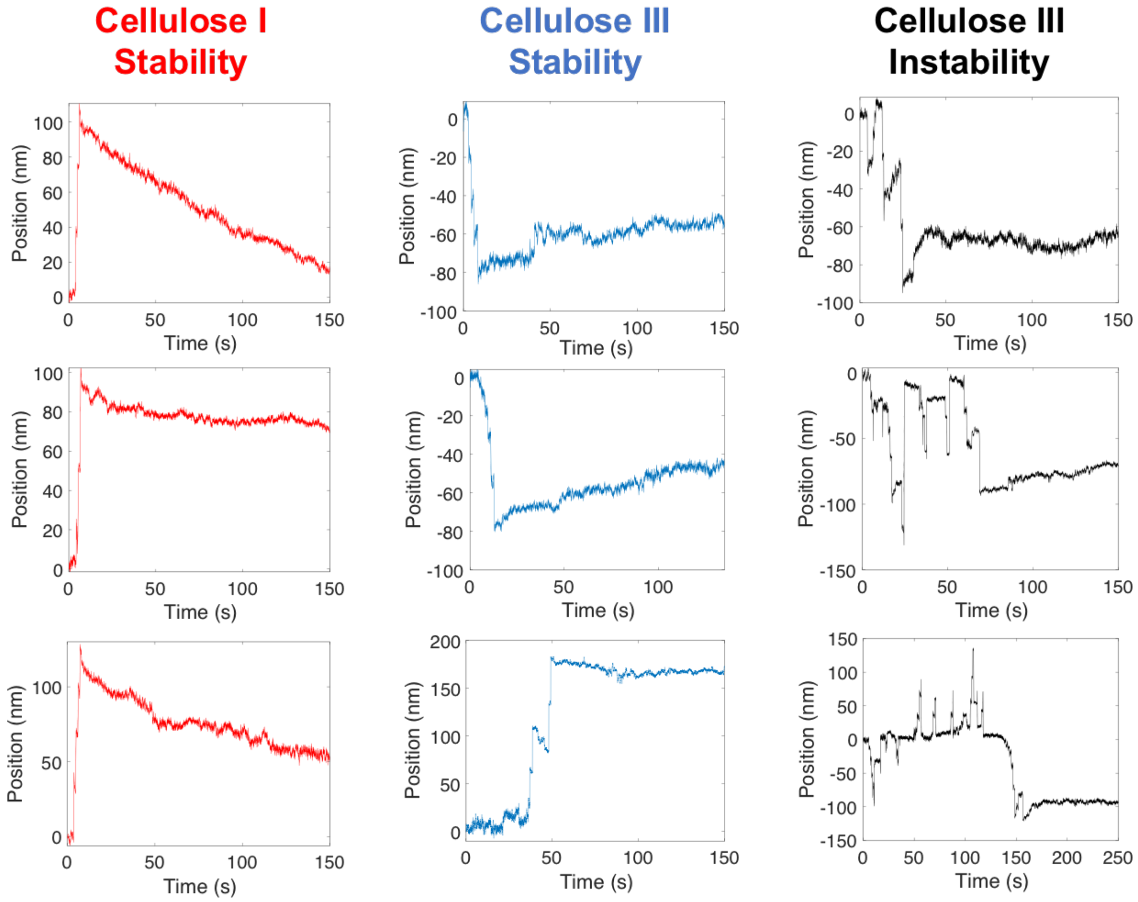

Fig. S3. Representative motility traces for Cel7A on Cladophora cellulose I. Dashed lines indicate average Cel7A velocities during processive hydrolysis of cellulose as 0.25 ± 0.35 nm s^{-1} (s.d.; for cellulose I; N=68; in red) and 0.17 ± 0.14 nm s^{-1} (s.d.; for cellulose III; N=30; in black).

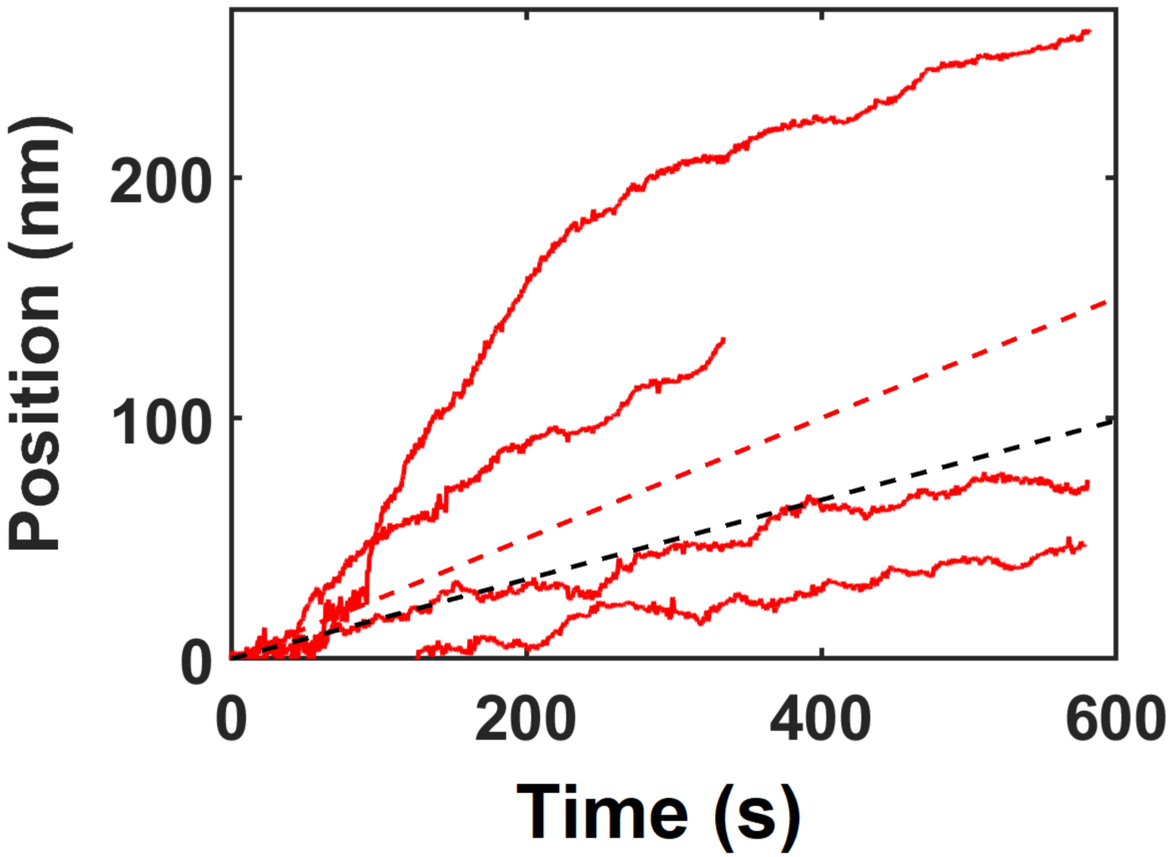

Fig. S4. Langmuir-type adsorption model fits (in red) for CBM1 binding data (in black) to Cladophora-derived Cellulose I (A, C, E) and Cellulose III (B, D, F).

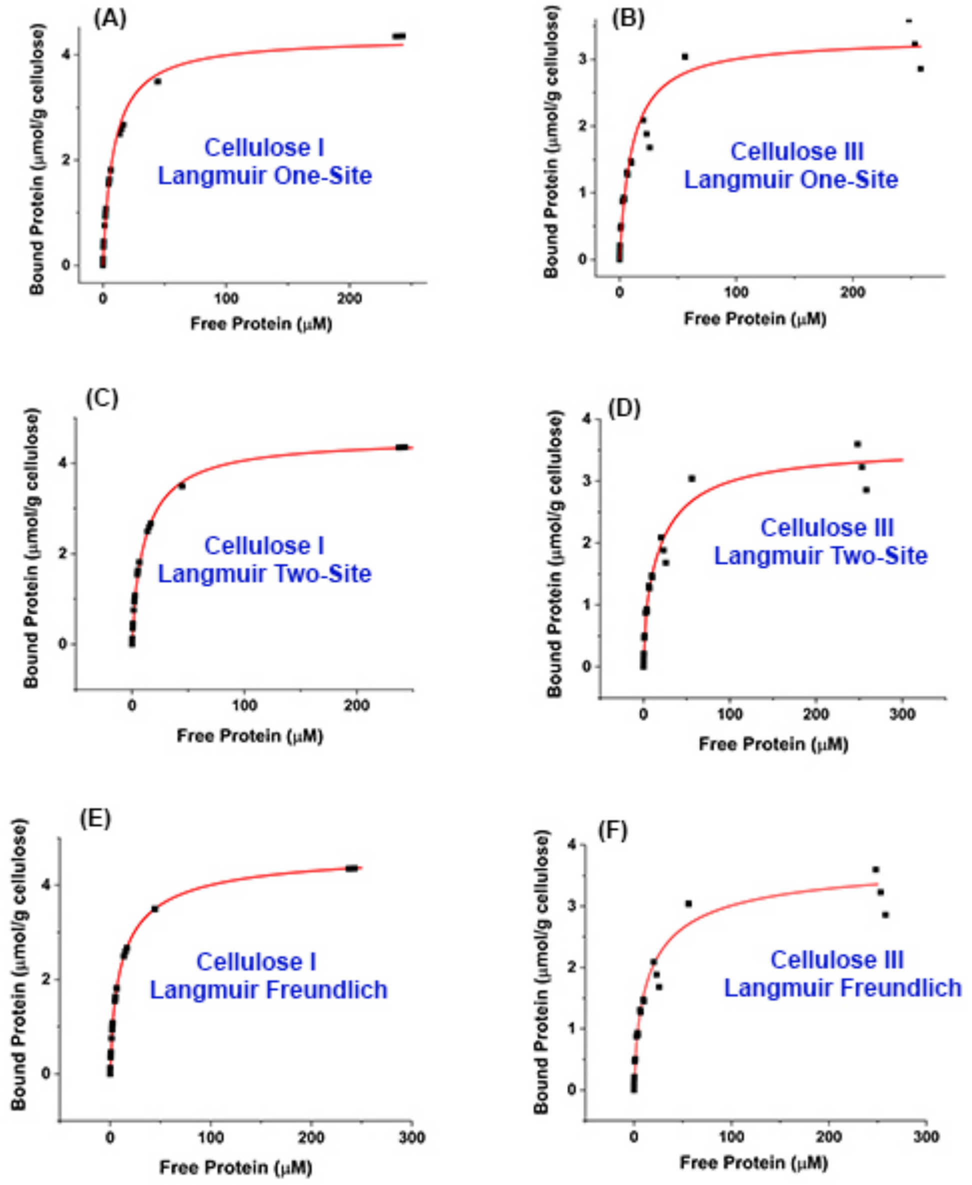

Fig. S5. (A) Side and cross-sectional snapshot view of CBM1-cellulose unbiased MD simulations conducted for distinct cellulose ultrastructures; cellulose I and cellulose III (with varying degrees of surface chain order or crystallinity index-CrI). Arrow direction indicates the preferred canonical binding orientation of CBM1 for cellulose I. (B) Average diffusivity of CBM1 on cellulose allomorph surfaces was estimated from the trajectories of the unbiased MD simulations to show significantly higher values for cellulose III versus cellulose I, again suggestive of weaker CBM1 binding interactions with the former substrate. (C) Steric clashes of planar CBM1 binding surface aromatic residues with cellulose III surface provides an atomistic basis for reduced binding towards cellulose-III. Root mean square fluctuations (RMSF) values seen for CBM1 planar binding surface tyrosines (Y5, Y31, and Y32) was significantly higher for cellulose III of decreasing crystallinity. (D) Representative image from MD simulations highlighting the improper stacking of Y5 residue on cellulose III is highlighted here.

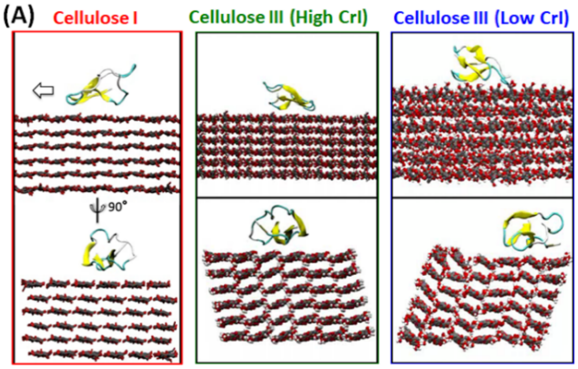

(B)

CBM1 Diffusivity (cm^2/s)	Cellulose I	Cellulose III (High CrI)	Cellulose III (High CrI)
ρ	1.4×10^{-11}	6×10^{-9}	2.3×10^{-5}

(C) Root mean square fluctuation (RMSF) of key residues

CBM1 Residue #	Cellulose I	Cellulose III (Low CrI)	Cellulose III (High CrI)
Y5	0.44	0.65	0.9
Y31	0.47	0.54	0.9
Y32	0.35	0.4	0.6

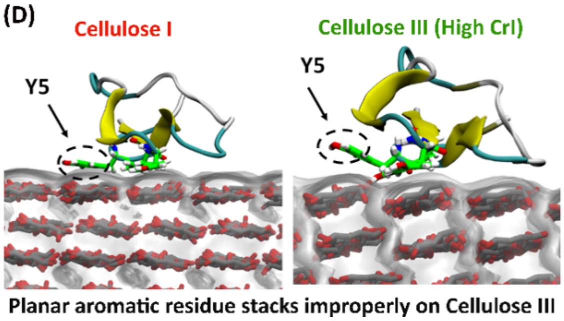

Fig. S6. Langmuir-type adsorption model fits (in red) for CBM3a binding data (in black) to Cladophora-derived Cellulose I (A, C, E) and Cellulose III (B, D, F).

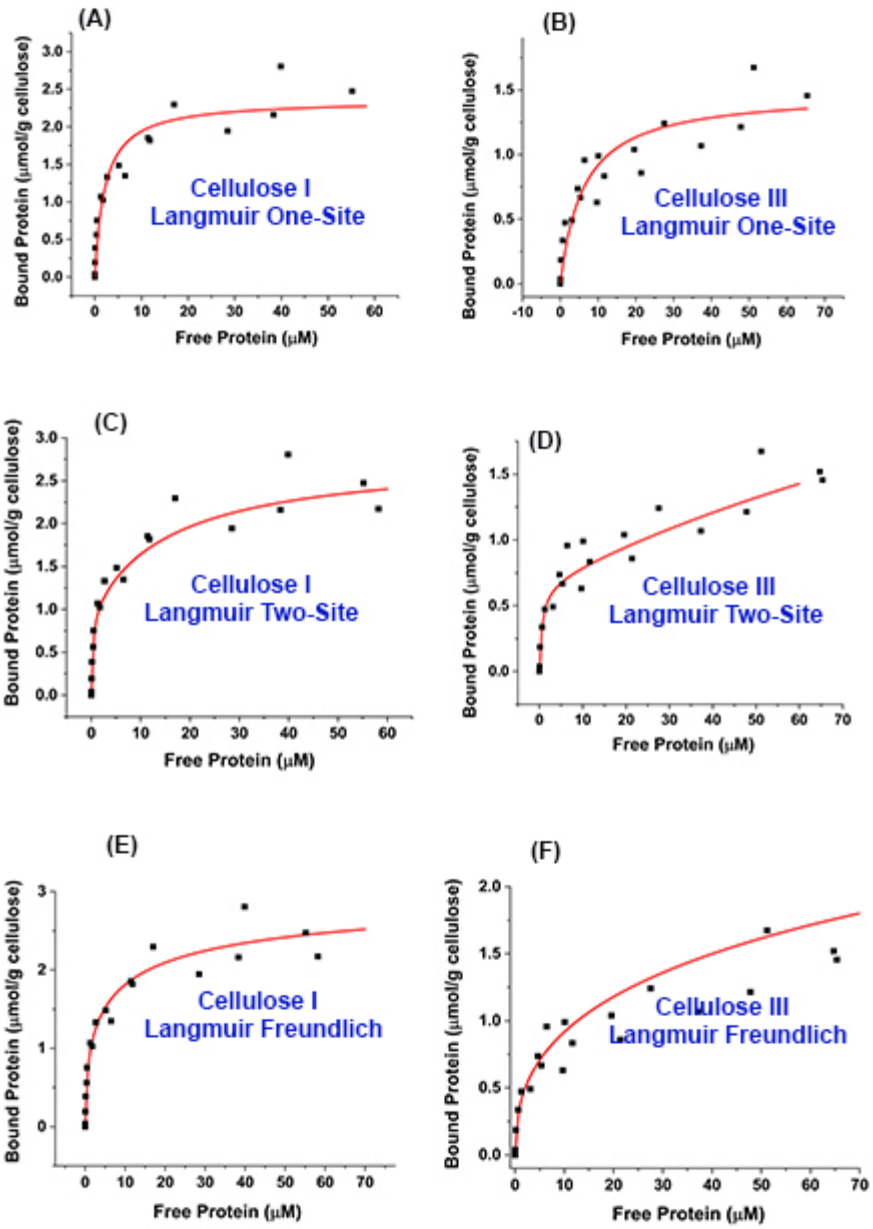

Fig. S7. Protein adsorption data and fitted partition coefficient slopes for various Type-A CBMs to Cladophora-derived Cellulose I (in black) and Cellulose III (in red).

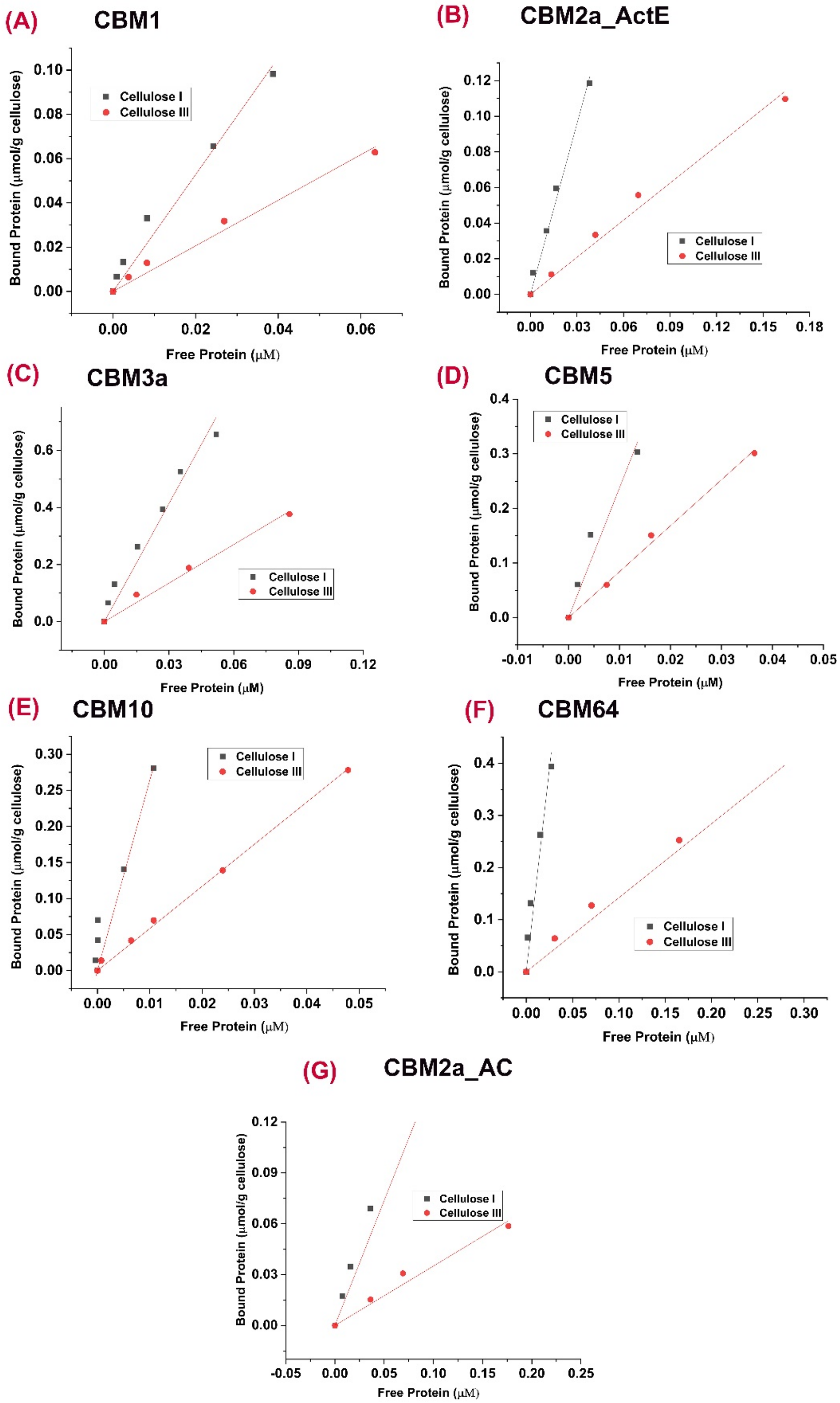

Fig. S8. Comparison of partition coefficients (liters/gram) estimated for various Type-A CBMs towards Cladophora based cellulose I (in red) and cellulose III (in black) are shown here. Error bars are standard deviations for reported mean values. The partition coefficient bars for GFP only control protein are not clearly visible since it exhibits very low binding to either cellulose allomorph.

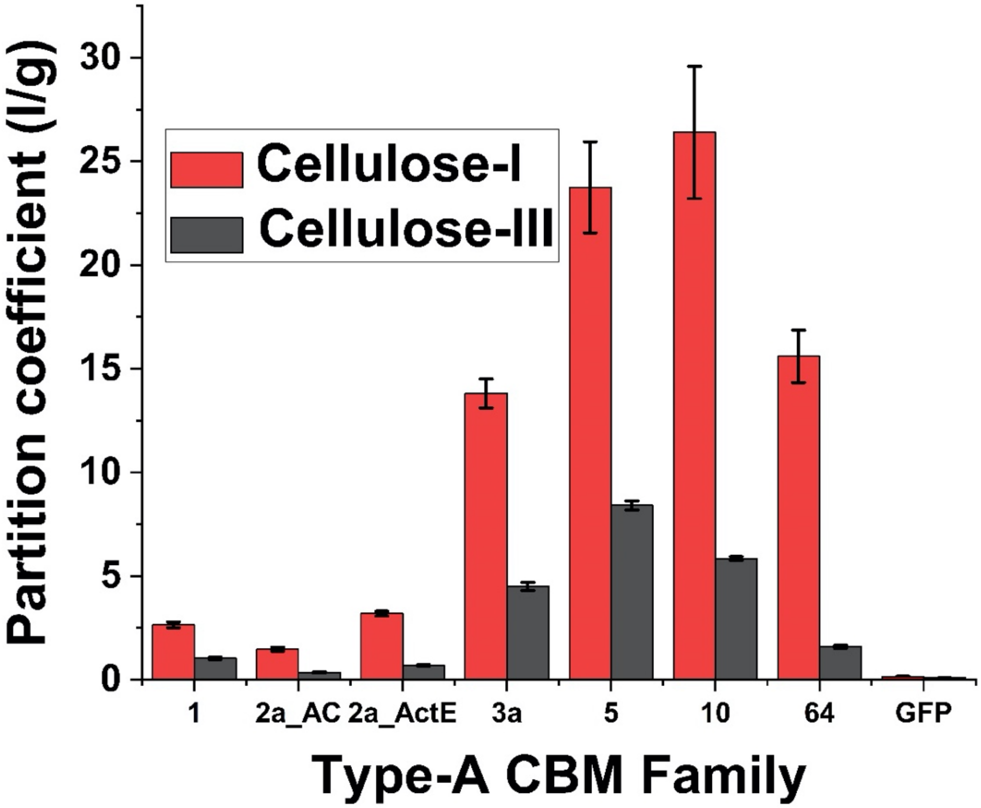

Fig. S9. Characterization of GFP-CBM3a binding kinetics to Cladophora cellulose I (CI) and cellulose III (CIII) using FRAP. Panel A-C show representative snapshots of the FRAP image acquisition with panel A as the image before photobleaching, B first frame after local photobleaching, and panel C at the end of the image acquisition, the scale bar is 10µm. Panel D and E show example recovery curves for cellulose I and III, respectively. Note that the shown recovery curves are baselined adjusted to zero as mentioned in the experimental procedures section.

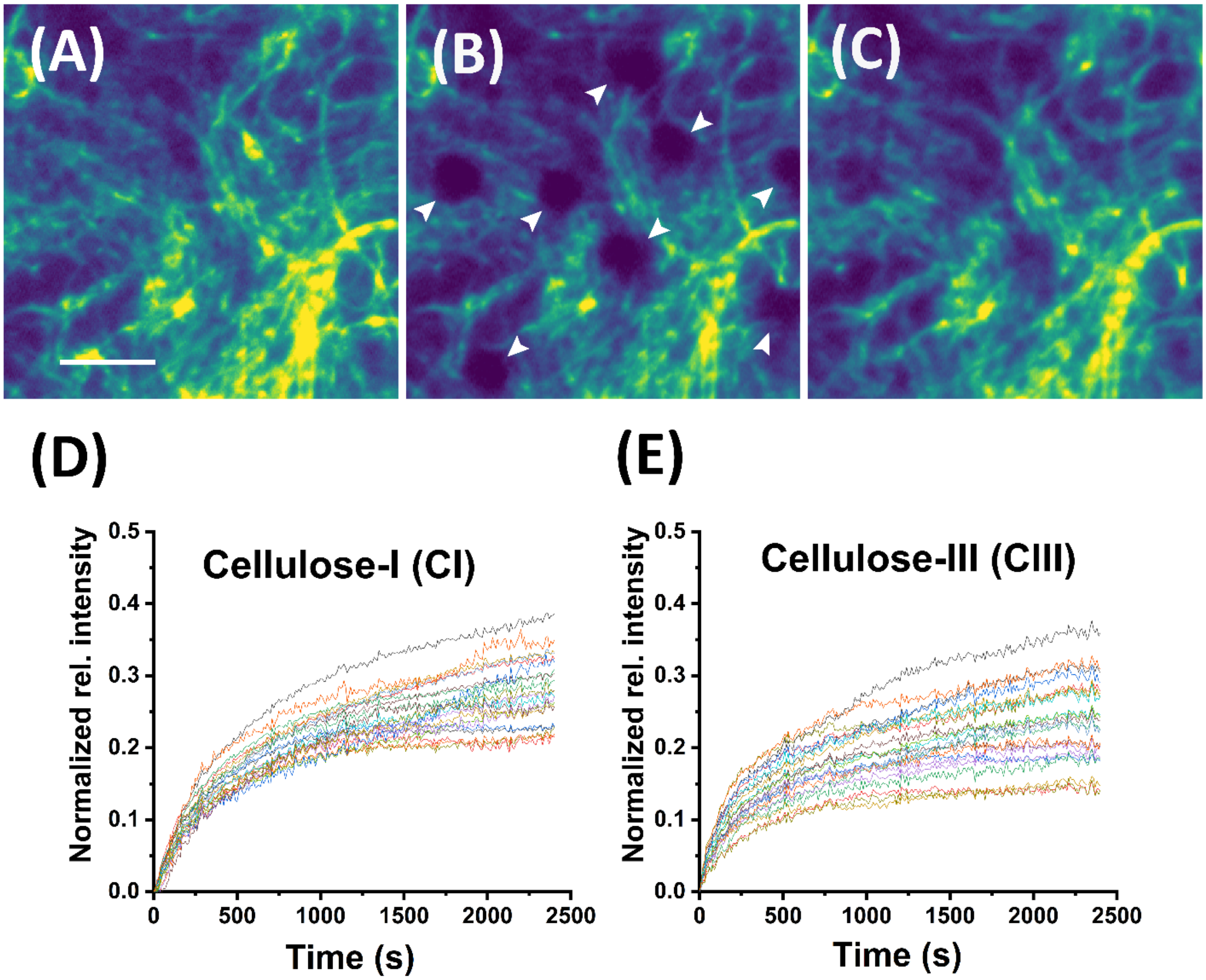

Fig. S10. Quartz crystal microbalance (QCM) based analysis of GFP-CBM3a binding parameters towards Avicel based cellulose I (CI) and cellulose III (CIII) nanofibrils. Representative QCM traces for binding/unbinding dynamics of GFP-CBM3a towards cellulose I (A) and cellulose III (B) are shown below. Representative binding model fits (in green) to raw QCM data (in black) is shown for binding (C) and unbinding (D) regimes for the sensorgram reported in (A).

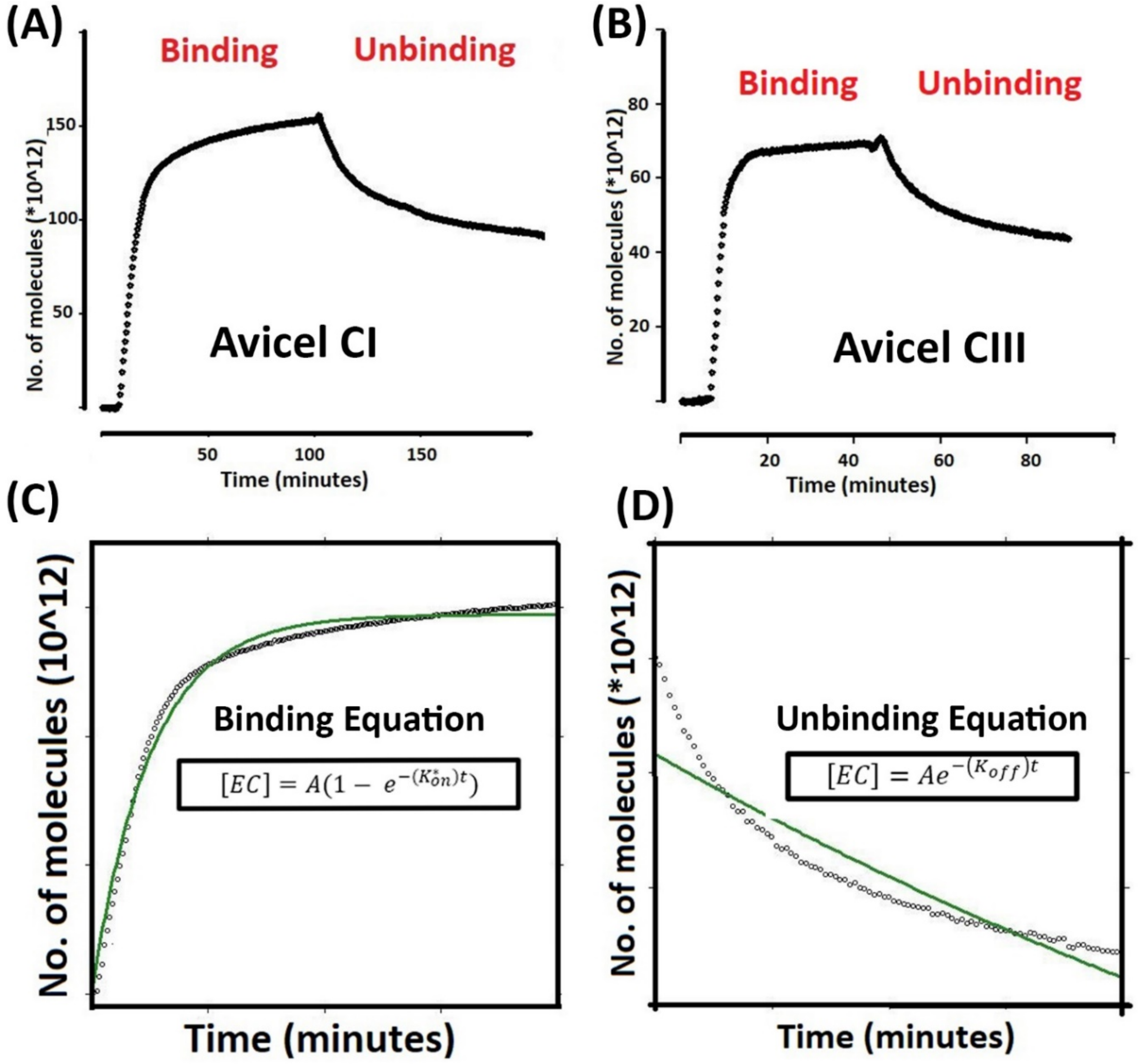

Fig. S11. (A) Force vs lifetime raw data scatterplot for the CBM1 non-covalent bonds to Cladophora celluloses I (blue) and III (brown) are shown here. Total number of individual rupture events measured (N) on cellulose I is 410 and on cellulose III is 214. For visual clarity, we omitted data points above 20 pN or 12 s from the scatterplot (31 for cellulose I; and 5 for cellulose III). But we did not exclude any data from our report or analysis. Our one-way ANOVA test (B) concluded that there was no significant difference (p=0.20) between the two entire datasets or at 2.5 pN intervals. Such a wide variance in lifetimes further supports our claim of multiple binding regimes.

(A)

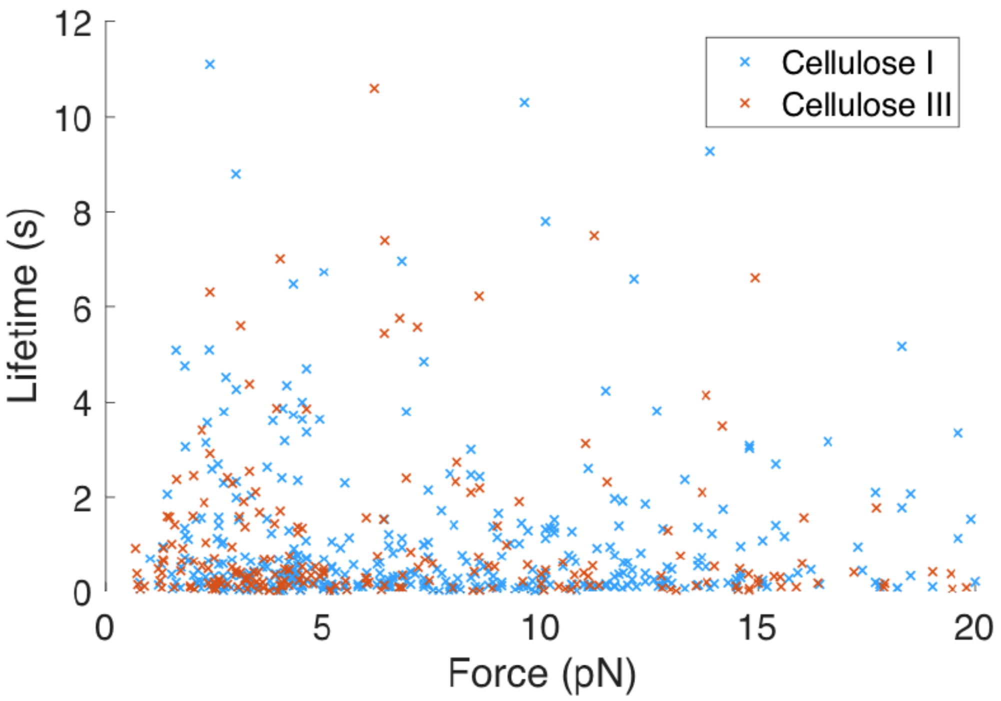

(B)

Force Range	Mean Lifetime Cellulose I (s)	Mean Lifetime Cellulose III (s)	ANOVA Result
All Forces	1.46 ± 0.22	1.01 ± 0.07	F(1,536) = 1.67, p = 0.20
0-2.5 pN	1.91 ± 0.60	1.00 ± 0.20	F(1,78) = 1.91, p = 0.17
2.5-5 pN	1.76 ± 0.59	1.07 ± 0.23	F(1,178) = 0.84, p = 0.36
5-7.5 pN	1.76 ± 0.70	1.59 ± 0.50	F(1,60) = 0.04, p = 0.85
7.5-10 pN	0.79 ± 0.21	1.18 ± 0.33	F(1,71) = 0.96, p = 0.33
10-12.5 pN	1.49 ± 0.60	1.02 ± 0.48	F(1,70) = 0.16, p = 0.69
12.5-15 pN	1.06 ± 0.31	1.01 ± 0.37	F(1,52) = 0.02, p = 0.93
15-17.5 pN	1.72 ± 0.84	0.45 ± 0.17	F(1,21) = 1.17, p =0.29
17.5-20 pN	1.23 ± 0.38	0.43 ± 0.23	F(1,20) = 1.82, p =0.19

Fig. S12. Force vs lifetime relationships for the CBM1 non-covalent bonds to Cladophora derived cellulose I (blue) and filter paper (green) derived cellulose microfibrils. Both force-lifetime distributions failed to converge to the classical slip bond model and revealed that the CBM1-cellulose interaction is multimodal across different native cellulosic substrates. Interestingly, the reported mean lifetime of the CBM1-filter paper cellulose bond (3.03 ± 0.37 SEM) is higher than that of the CBM1-cladophora cellulose bond (1.41 ± 0.20 SEM).

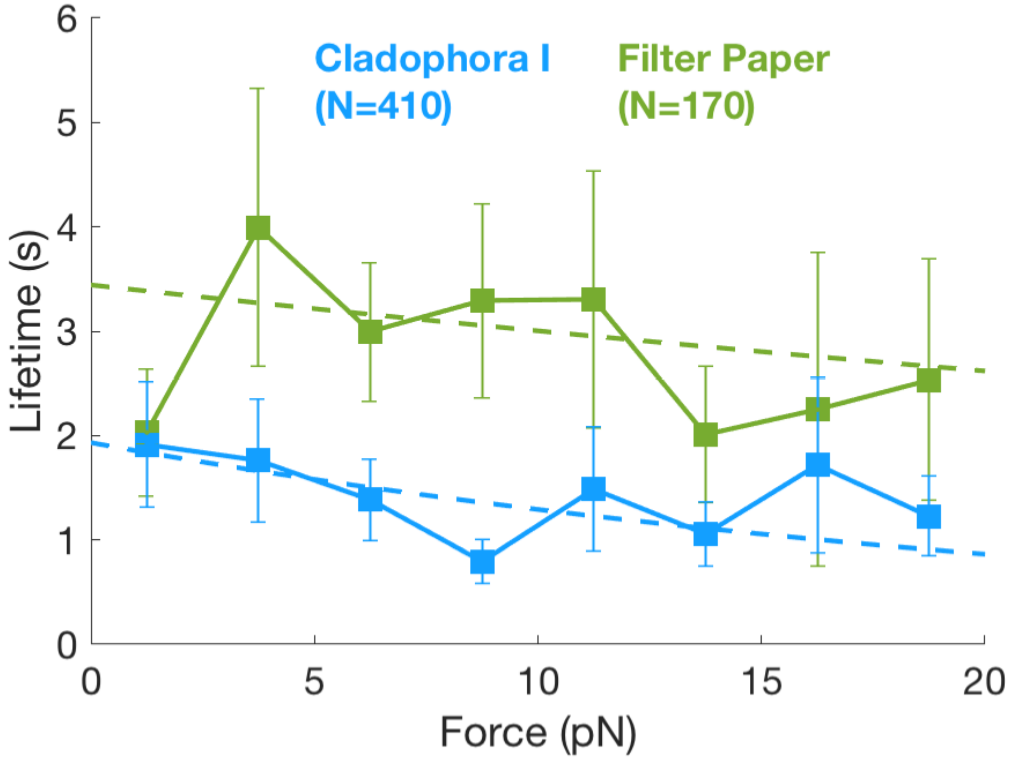

Fig. S13. (A) Force vs lifetime raw data scatterplot for the CBM1 non-covalent bond on *Cladophora* cellulose I using an anti-His Fab in the assay construct (blue) and using full anti-His antibody (brown). Total number of events measured (N) using the full antibody is 233 and using the Fab is 187. For visual clarity, we omitted data points above 20 pN or 12 s from the scatterplot (8 for full antibody; 23 for Fab). We did not exclude any data from our report or analysis. Our one-way ANOVA test (B) concluded that there was no significant difference between the two entire datasets or at 5 pN intervals. Because of the statistical similarity, we combined both datasets to represent our CBM1-cellulose I data.

(A)

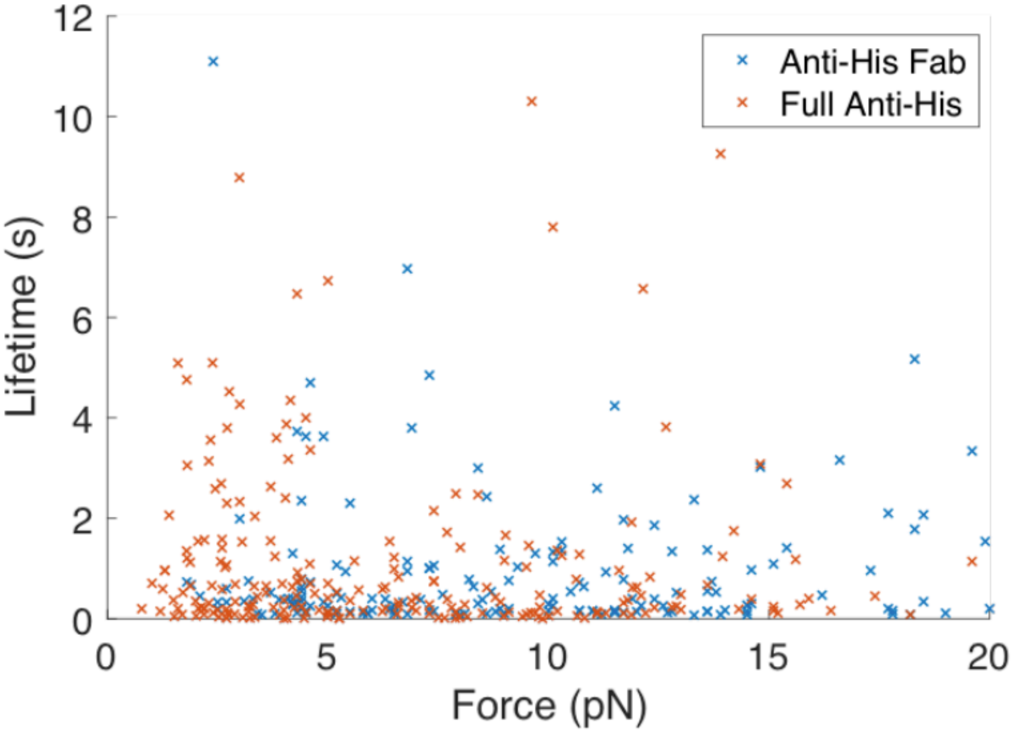

(B)

Force Range	Mean Lifetime Anti-His Fab (s)	Mean Lifetime Full Anti-His (s)	ANOVA Result
All Forces	1.78 ± 0.37	1.04 ± 0.18	$F(1,370) = 2.74$, $p = 0.10$
0-5 pN	1.91 ± 0.60	1.55 ± 0.57	$F(1,149) = 0.13$, $p = 0.72$
5-10 pN	1.29 ± 0.41	0.83 ± 0.18	$F(1,104) = 1.10$, $p = 0.30$
10-15 pN	2.19 ± 0.90	0.75 ± 0.12	$F(1,86) = 3.00$, $p = 0.09$
15-20 pN	1.66 ± 1.06	1.28 ± 0.40	$F(1,25) = 0.13$, $p = 0.72$

Fig. S14. (A) Force vs lifetime raw data scatterplot for the CBM1 non-covalent bond on *Cladophora* cellulose I using the wild-type CBM1 protein (blue) and using the Y31A CBM1 mutant (brown). Total number of events measured (N) using CBM1 is 410 and using the mutant is 93. For visual clarity, we omitted data points above 20 pN or 12 s from the scatterplot (31 for CBM1; 11 for Y31A-CBM1). We did not exclude any data from our report or analysis. Our one-way ANOVA test (B) concluded that there was no significant difference between the two entire datasets or at the 0-5 pN, 5-10 pN, and 15-20 pN ranges. However, there was a significant difference observed at the 10-15 pN range indicating that structural changes on CBM does indeed affect the CBM1-cellulose interactions measured using our single-molecule rupture assay method. Future single molecule studies could explore the effects of other protein structural changes on binding to cellulose.

(A)

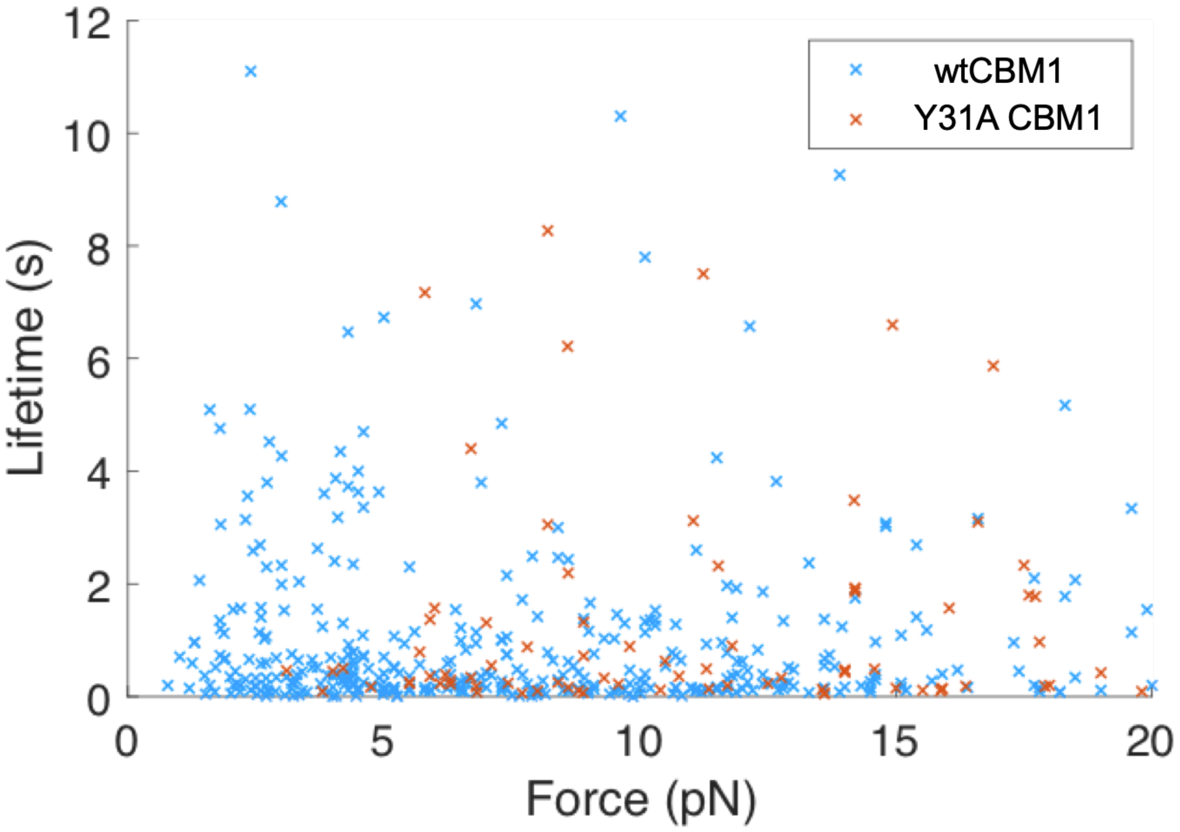

(B)

Force Range	Mean Lifetime wtCBM1 (s)	Mean Lifetime Y31A CBM (s)	ANOVA Result
All Forces	1.46 ± 0.22	2.25 ± 0.48	F(1,455) = 2.36, p = 0.13
0-5 pN	1.80 ± 0.46	0.33 ± 0.08	F(1,154) = 0.34, p=0.56
5-10 pN	1.06 ± 0.22	1.94 ± 0.71	F(1,139) = 2.44, p = 0.12
10-15 pN	1.34 ± 0.40	3.55 ± 1.08	F(1,115) = 5.66, p = 0.02
15-20 pN	1.45 ± 0.51	1.19 ± 0.39	F(1,41) = 0.13, p = 0.71

Fig. S15. Langmuir-type adsorption data (symbols) or model (dotted lines) fits (A) and Scatchard-plot representation of adsorption data (B) for Calcofluor White dye binding data to Cladophora-derived Cellulose I (in red) and Cellulose III (in grey). Here, the dotted lines represent Langmuir-two site model fits. Error bars depict standard deviation from the reported means for replicate measurements (often hidden due to size of symbol).

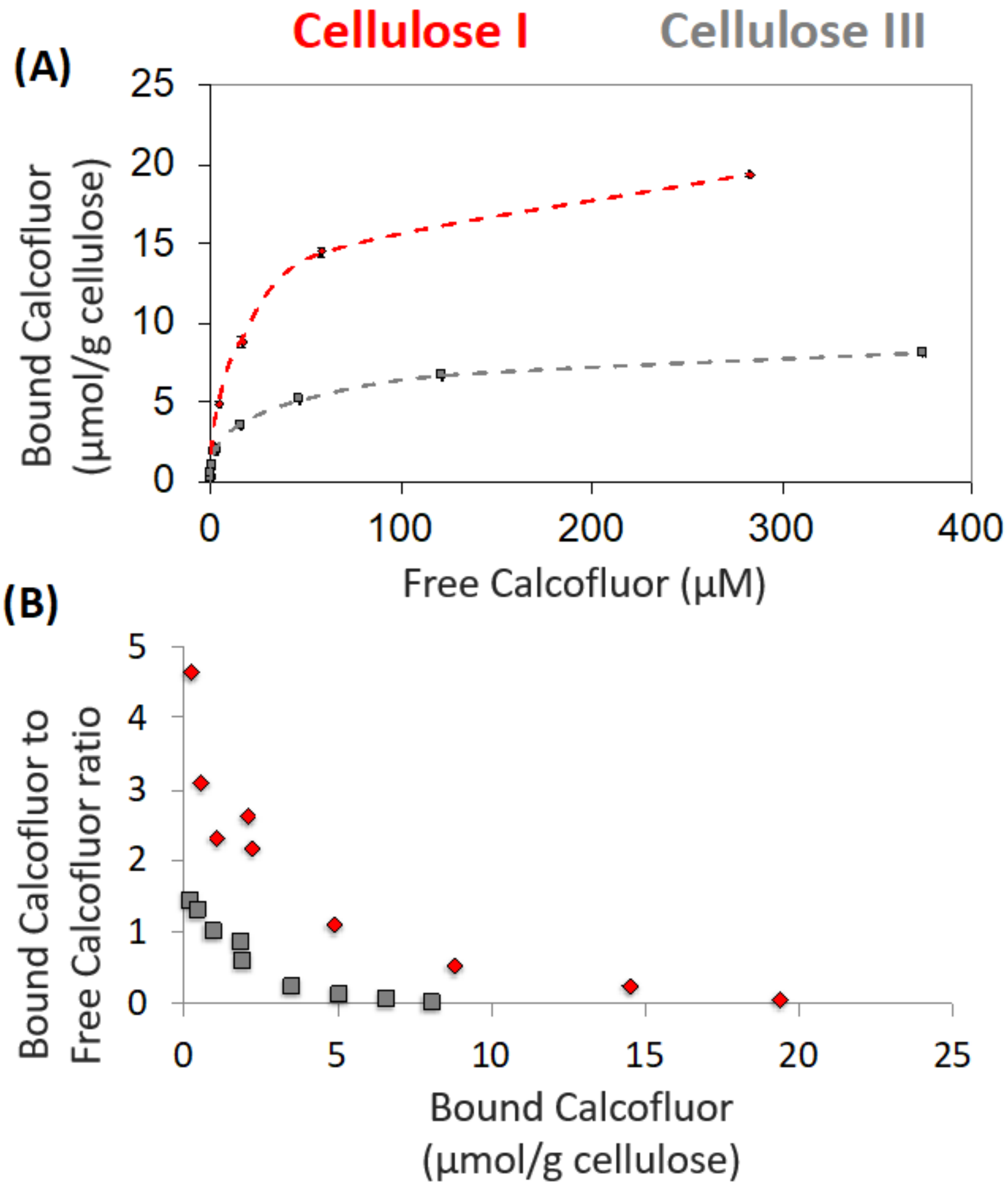

Fig. S16. Buffon needle problem inspired geometric probability model to determine likely orientations of CBM protein on flat cellulose binding surface. (A) Crystal structure of CBM1 (PDB code: 1CBH) represented as a needle (solid red line) of length 2.08 nm, (B) Hydrophobic face of cellulose I represented as an array of parallel lines (dotted black line) with a spacing of 0.8 nm, (C) Buffon needle inspired CBM-cellulose model schematic to determine geometric probability of all possible orientations of CBM1 on cellulose surface. The original Buffon model formulation is discussed in his classical 1777 paper (44)**.** (D) Monte Carlo simulation methodology flowchart (see SI Appendix Experimental Procedures section).

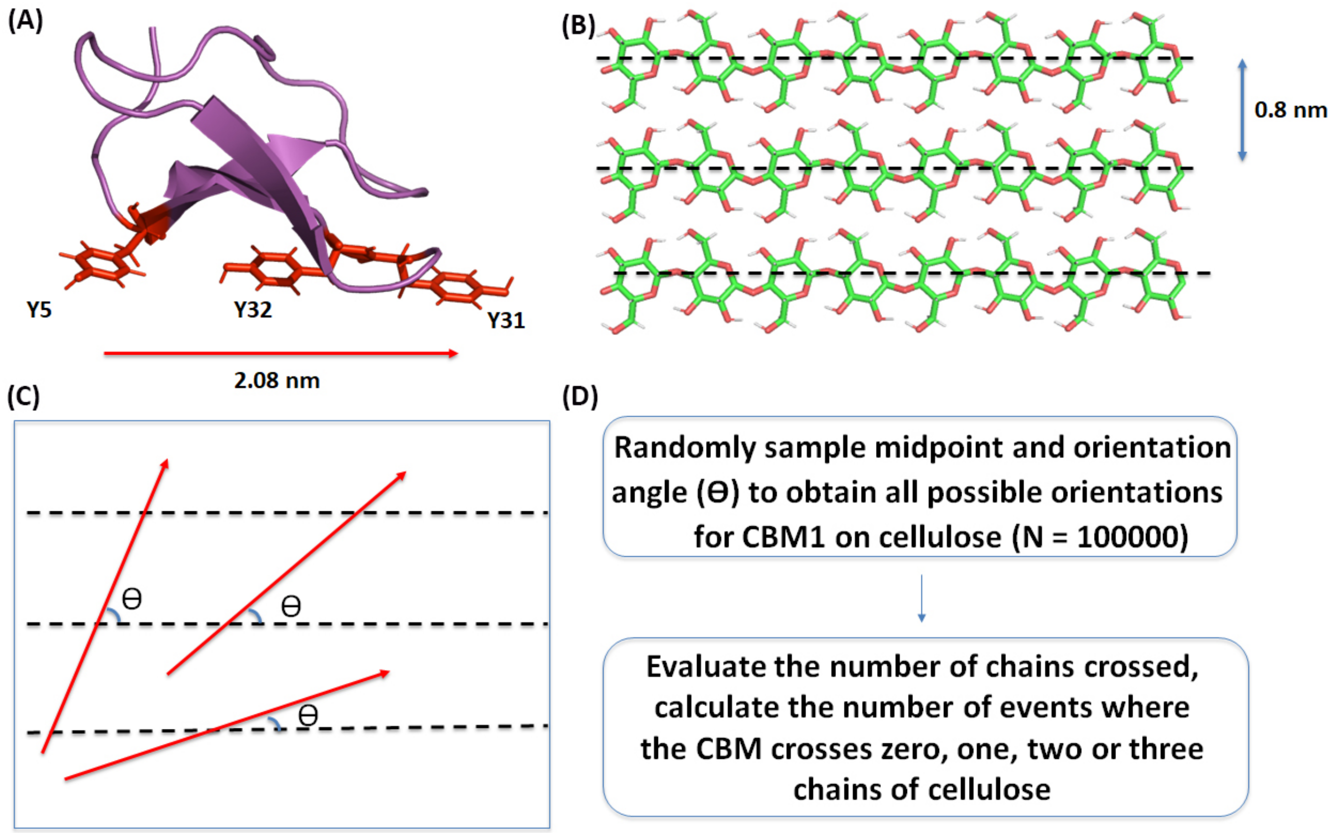

Table S1. Specific activity of Cel7A cellulase alone towards Cladophora cellulose I and cellulose III at 0.5, 2.5, and 10 mg enzyme per g cellulose loading. Specific activity (mean ± s.d.) is reported here in terms of nmol cellobiose released per μmol enzyme per minute of cellulose hydrolysis reaction.

	Cellulose - I	Cellulose-III
Enzyme loading (mg/g)	Specific Activity (nmol cellobiose per μmol enzyme per min)	
0.5	783.5 ± 324	909.3 ± 285
2.5	225.8 ± 23.6	787.8 ± 74.8
10	146.0 ± 73.0	426.0 ± 123

Table S2. Sensitivity analysis was performed by changing the predicted Langmuir one-site model parameters by 10% or 20% and checking for goodness of fit (i.e., root-mean square error or RMSE) of the original CBM1 binding dataset.

Sensitivity analysis for one-site model		
		RMSE
Original data predicted n_{max} & K_{d}		0.17
10% change in original model parameters	$0.90n_{\mathrm{max}}$	0.23
	$1.10n_{\mathrm{max}}$	0.17
	$0.90K_{\mathrm{d}}$	0.17
	$1.10K_{\mathrm{d}}$	0.17
20% change in original model parameters	$0.80n_{\mathrm{max}}$	0.35
	$1.20n_{\mathrm{max}}$	0.35
	$0.80K_{\mathrm{d}}$	0.20
	$1.20K_{\mathrm{d}}$	0.19

Table S3. CBM-cellulose Buffon needle model simulation predicts that wild-type (WT) CBM1 is equally likely to align and bind with its aromatic binding residues aligned both along a single (58% probability) or across multiple (42% probability) cellulose chains. However, the mutant CBM1 (Y31A) with a 'shortened' needle is more likely to align and bind along a single cellulose chain (90% probability).

Protein Type	Along the chain events	Across the chain events
CBM1 WT	58%	42%
CBM1 Y31A	90%	10%

Movie S1. Snapshot of side-view (top) and cross-sectional (bottom) view of CBM1-cellulose I unbiased MD simulations.

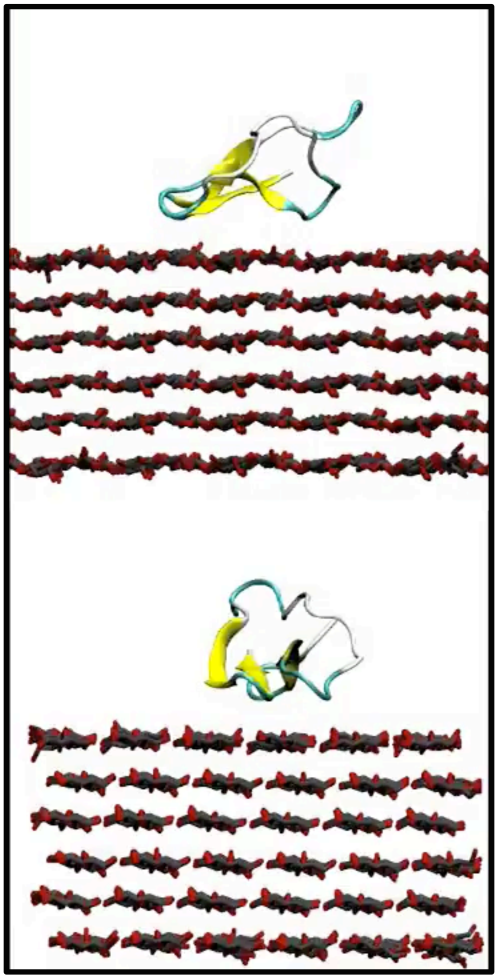

Movie S2. Snapshot of side-view (top) and cross-sectional (bottom) view of CBM1-cellulose III (high crystallinity) unbiased MD simulations.

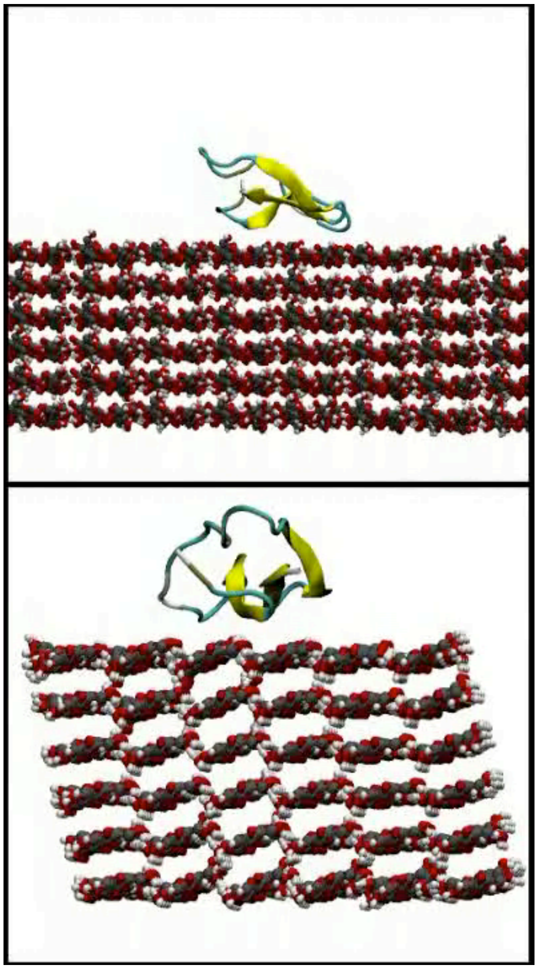

Movie S3. Snapshot of side-view (top) and cross-sectional (bottom) view of CBM1-cellulose III (low crystallinity) unbiased MD simulations.

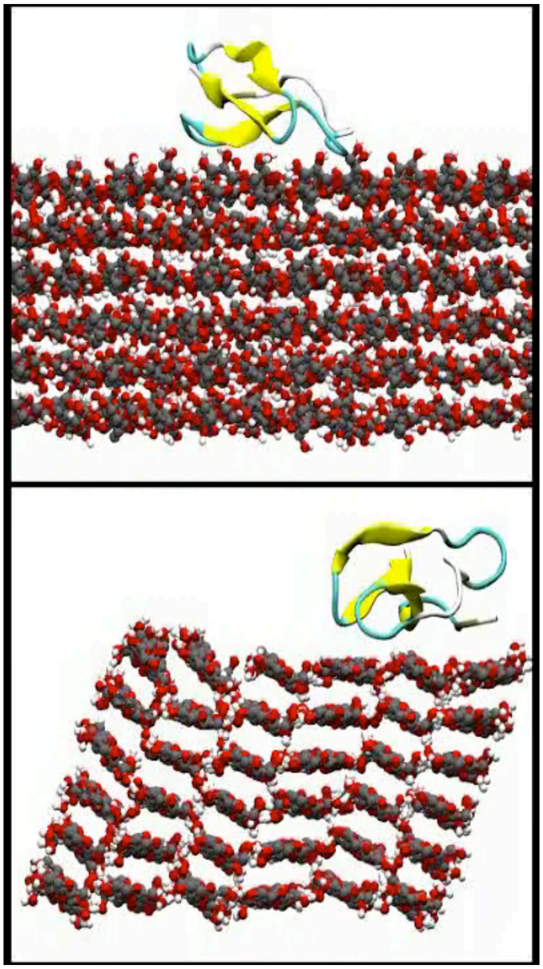

SI Appendix References:

1. Brady, S. K., Sreelatha, S., Feng, Y., Chundawat, S. P. S., and Lang, M. J. (2015) Cellobiohydrolase 1 from *Trichoderma reesei* degrades cellulose in single cellobiose steps. *Nat. Commun.* **6**, 10149

2. Chundawat, S. P. S., Bellesia, G., Uppugundla, N., Sousa, L., Gao, D., Cheh, A., Agarwal, U., Bianchetti, C., Phillips, G., Langan, P., Balan, V., Gnanakaran, S., and Dale, B. E. (2011) Restructuring the crystalline cellulose hydrogen bond network enhances its depolymerization rate. *J. Am. Chem. Soc.* **133**, 11163–11174

3. Sousa, L. da C., Humpula, J., Balan, V., Dale, B. E., and Chundawat, S. P. S. (2019) Impact of ammonia pretreatment conditions on the cellulose III allomorph ultrastructure and its enzymatic digestibility. *ACS Sustain. Chem. Eng.* **7**, 14411–14424

4. Ruland, W. (1961) X-ray determination of crystallinity and diffuse disorder scattering. *Acta Cryst.* **14**, 1180–1185

5. Park, S., Baker, J., Himmel, M., Parilla, P., and Johnson, D. (2010) Cellulose crystallinity index: measurement techniques and their impact on interpreting cellulase performance. *Biotechnol. Biofuels*. **3**, 10

6. French, A. D. (2013) Idealized powder diffraction patterns for cellulose polymorphs. *Cellulose*. **21**, 885–896

7. Hult, L., Iversen, T., and Sugiyama, J. (2003) Characterization of the supramolecular structure of cellulose in wood pulp fibres. *Cellulose*. **10**, 103–110

8. He, J., Cui, S., and Wang, S.-Y. (2008) Preparation and crystalline analysis of high-grade bamboo dissolving pulp for cellulose acetate. *J Appl Polym Sci*. **107**, 1029–1038

9. Garvey, C. J., Parker, I. H., and Simon, G. P. (2005) On the interpretation of x-ray diffraction powder patterns in terms of the nanostructure of cellulose I fibres. *Macromol. Chem. Phys.* **206**, 1568–1575

10. Chundawat, S. P. S., Sousa, L. daCosta, Roy, S., Yang, Z., Gupta, S., Pal, R., Zhao, C., Liu, S.-H., Petridis, L., O'Neill, H., and Pingali, S. V. (2020) Ammonia-salt solvent promotes cellulosic biomass deconstruction under ambient pretreatment conditions to enable rapid soluble sugar production at ultra-low enzyme loadings. *Green Chem.* **22**, 204–218

11. Atalla, R. H., and Vanderhart, D. L. (1984) Native cellulose: a composite of two distinct crystalline forms. *Science (80-.).* **223**, 283–285

12. Agarwal, U., Reiner, R., and Ralph, S. (2010) Cellulose I crystallinity determination using FT–Raman spectroscopy: univariate and multivariate methods. *Cellulose*. **17**, 721–733

13. Agarwal, U. P. (2014) 1064 nm FT-Raman spectroscopy for investigations of plant cell walls and other biomass materials. *Front. Plant Sci.* **5**, 490

14. Atalla, R. J., and Vanderhart, D. L. (1987) Studies on the structure of cellulose using raman spectroscopy and solid state 13C-NMR. in *Institute for Paper Chemistry (IPC Technical Paper Series 217)*

15. Wiley, J. H., and Atalla, R. H. (1987) Band assignments in the raman spectra of celluloses. *Carbohydr. Res.* **160**, 113–129

16. Chundawat, S. P. S., Lipton, M. S., Purvine, S. O., Uppugundla, N., Gao, D., Balan, V., and Dale, B. E. (2011) Proteomics based compositional analysis of complex cellulase-hemicellulase mixtures. *J. Proteome Res.* **10**, 4365–4372

17. Gao, D., Chundawat, S. P. S., Sethi, A., Balan, V., Gnanakaran, S., and Dale, B. E. (2013) Increased enzyme binding to substrate is not necessary for more efficient cellulose hydrolysis. *Proc. Natl. Acad. Sci.* **110**, 10922–10927

18. Gao, D., Chundawat, S. P. S., Krishnan, C., Balan, V., and Dale, B. E. (2010) Mixture optimization of six core glycosyl hydrolases for maximizing saccharification of ammonia fiber expansion (AFEX) pretreated corn stover. *Bioresour. Technol.* **101**, 2770–2781

19. Whitehead, T. A., Bandi, C. K., Berger, M., Park, J., and Chundawat, S. P. S. (2017) Negatively supercharging cellulases render them lignin-resistant. *ACS Sustain. Chem. Eng.* **5**, 6247–6252

20. Lim, S., Chundawat, S. P. S., and Fox, B. G. (2014) Expression, purification and characterization of a functional carbohydrate-binding module from *Streptomyces sp. SirexAA-E*. *Protein Expr. Purif.* **98**, 1–9

21. Klock, H. E., Lesley, S. A., and Doyle, S. A. (2009) The Polymerase Incomplete Primer Extension (PIPE) Method Applied to High-Throughput Cloning and Site-Directed Mutagenesis High Throughput Protein Expression and Purification. *Methods Mol. Biol.* **498**, 91–103

22. Takasuka, T. E. ., Walker, J. A. ., Bergeman, L. F. ., Vander Meulen, K. A. ., Makino, S. I. ., Elsen, N. L. ., and Fox, B. G. (2014) Cell-free translation of biofuels enzymes. *Methods Mol. Biol.* **1118**, 71–95

23. Takasuka, T. E., Book, A. J., Lewin, G. R., Currie, C. R., and Fox, B. G. (2013) Aerobic deconstruction of cellulosic biomass by an insect-associated Streptomyces. *Sci. Rep.* **3**, 1030

24. Studier, F. W. (2005) Protein production by auto-induction in high-density shaking cultures. *Protein Expr. Purif.* **41**, 207–234

25. Kavoosi, M., Meijer, J., Kwan, E., Creagh, A. L., Kilburn, D. G., and Haynes, C. A. (2004) Inexpensive one-step purification of polypeptides expressed in Escherichia coli as fusions with the family 9 carbohydrate-binding module of xylanase 10A from T. maritima. *J. Chromatogr. B*. **807**, 87–94

26. Hong, J., Ye, X., Wang, Y., and Zhang, Y. H. P. (2008) Bioseparation of recombinant cellulose-binding module-proteins by affinity adsorption on an ultra-high-capacity cellulosic adsorbent. *Anal. Chim. Acta*. **621**, 193–199

27. McLean, B. W., Bray, M. R., Boraston, A. B., Gilkes, N. R., Haynes, C. A., and Kilburn, D. G. (2000) Analysis of binding of the family 2a carbohydrate-binding module from Cellulomonas fimi xylanase 10A to cellulose: specificity and identification of functionally important amino acid residues. *Protein Eng.* **13**, 801–809

28. Hu, W., Xie, J., Chau, H. W., and Si, B. C. (2015) Evaluation of parameter uncertainties in nonlinear regression using Microsoft Excel Spreadsheet. *Environ. Syst. Res.* 10.1186/s40068-015-0031-4

29. Linder, M., and Teeri, T. T. (1996) The cellulose-binding domain of the major cellobiohydrolase of *Trichoderma reesei* exhibits true reversibility and a high exchange rate on crystalline cellulose. *Proc. Natl. Acad. Sci.* **93**, 12251–12255

30. Goldstein, M. A., Takagi, M., Hashida, S., Shoseyov, O., Doi, R. H., and Segel, I. H. (1993) Characterization of the cellulose-binding domain of the Clostridium cellulovorans cellulose-binding protein A. *J. Bacteriol.* **175**, 5762–5768

31. Bhagia, S., Dhir, R., Kumar, R., and Wyman, C. E. (2018) Deactivation of cellulase at the air-liquid interface is the main cause of incomplete cellulose conversion at low enzyme loadings. *Sci. Rep.* **8**, 1350

32. Lindorff-Larsen, K., Piana, S., Palmo, K., Maragakis, P., Klepeis, J. L., Dror, R. O., and Shaw, D. E. (2010) Improved side-chain torsion potentials for the Amber ff99SB protein force field. *Proteins Struct. Funct. Bioinforma.* **78**, 1950–1958

33. Boonstra, S., Onck, P. R., and van der Giessen, E. (2016) CHARMM TIP3P water model suppresses peptide folding by solvating the unfolded state. *J. Phys. Chem. B*. **120**, 3692–3698

34. Kirschner, K. N., Yongye, A. B., Tschampel, S. M., González-Outeiriño, J., Daniels, C. R., Foley, B. L., and Woods, R. J. (2008) GLYCAM06: A generalizable biomolecular force field. Carbohydrates. *J. Comput. Chem.* **29**, 622–655

35. López, C. A., Bellesia, G., Redondo, A., Langan, P., Chundawat, S. P. S., Dale, B. E., Marrink, S. J., and Gnanakaran, S. (2015) MARTINI coarse-grained model for crystalline cellulose microfibers. *J. Phys. Chem. B*. **119**, 465–473

36. Nishiyama, Y., Langan, P., and Chanzy, H. (2002) Crystal structure and hydrogen-bonding system in cellulose Iβ from synchrotron x-ray and neutron fiber diffraction. *J. Am. Chem. Soc.* **124**, 9074–9082

37. Wada, M., Chanzy, H., Nishiyama, Y., and Langan, P. (2004) Cellulose III crystal structure and hydrogen bonding by synchrotron x-ray and neutron fiber diffraction. *Macromolecules*. **37**, 8548–8555

38. Beckham, G. T., Matthews, J. F., Bomble, Y. J., Bu, L., Adney, W. S., Himmel, M. E., Nimlos, M. R., and Crowley, M. F. (2010) Identification of amino acids responsible for processivity in a family 1 carbohydrate-binding module from a fungal cellulase. *J. Phys. Chem. B*. **114**, 1447–1453

39. Nimlos, M. R., Beckham, G. T., Matthews, J. F., Bu, L., Himmel, M. E., and Crowley, M. F. (2012) Binding preferences, surface attachment, diffusivity, and orientation of a family 1 carbohydrate-binding module on cellulose. *J. Biol. Chem.* **287**, 20603–20612

40. Nishiyama, Y., Sugiyama, J., Chanzy, H., and Langan, P. (2003) Crystal structure and hydrogen bonding system in cellulose Iα from synchrotron x-ray and neutron fiber diffraction. *J. Amer. Chem. Soc.* **125**, 14300–14306

41. Brunecky, R., Subramanian, V., Yarbrough, J. M., Donohoe, B. S., Vinzant, T. B., Vanderwall, T. A., Knott, B. C., Chaudhari, Y. B., Bomble, Y. J., Himmel, M. E., and Decker, S. R. (2020) Synthetic fungal multifunctional cellulases for enhanced biomass conversion. *Green Chem.* **22**, 478–489

42. Lee, S.-H. (2018) Optimal integration of wide field illumination and holographic optical tweezers

for multimodal microscopy with ultimate flexibility and versatility. *Opt. Express*. **26**, 8049

43. Moran-Mirabal, J. M., Bolewski, J. C., and Walker, L. P. (2011) Reversibility and binding kinetics of *Thermobifida fusca* cellulases studied through fluorescence recovery after photobleaching microscopy. *Biophys. Chem.* **155**, 20–8

44. Buffon, G. (1777) Essai d'arithmétique morale. in *Histoire naturelle, générale er particulière, Supplément 4*, pp. 46–123

45. Wood, G. R., and Robertson, J. M. (1998) Buffon got it straight. *Stat. Probab. Lett.* **37**, 415–421

46. Creagh, A. L., Ong, E., Jervis, E., Kilburn, D. G., and Haynes, C. A. (1996) Binding of the cellulose-binding domain of exoglucanase Cex from *Cellulomonas fimi* to insoluble microcrystalline cellulose is entropically driven. *Proc. Natl. Acad. Sci. U. S. A.* **93**, 12229–12234

47. Sugiyama, J., Harada, H., and Saiki, H. (1987) Crystalline morphology of Valonia macrophysa cellulose III_I revealed by direct lattice imaging. *Int. J. Biol. Macromol.* **9**, 122–130

48. Bellesia, G., Chundawat, S. P. S., Langan, P., Dale, B. E., and Gnanakaran, S. (2011) Probing the early events associated with liquid ammonia pretreatment of native crystalline cellulose. *J. Phys. Chem. B*. **115**, 9782–9788

49. Sawada, D., Hanson, L., Wada, M., Nishiyama, Y., and Langan, P. (2014) The initial structure of cellulose during ammonia pretreatment. *Cellulose*. **21**, 1117–1126

50. Wada, M., Nishiyama, Y., Bellesia, G., Forsyth, T., Gnanakaran, S., and Langan, P. (2011) Neutron crystallographic and molecular dynamics studies of the structure of ammonia-cellulose I: rearrangement of hydrogen bonding during the treatment of cellulose with ammonia. *Cellulose*. **18**, 191–206

51. Abbott, D. W., and Boraston, A. B. (2012) Quantitative approaches to the analysis of carbohydrate-binding module function. *Methods Enzymol.* **510**, 211–31

52. Sugimoto, N., Igarashi, K., Wada, M., and Samejima, M. (2012) Adsorption characteristics of fungal family 1 cellulose-binding domain from *Trichoderma reesei* cellobiohydrolase I on crystalline cellulose: Negative cooperative adsorption via a steric exclusion effect. *Langmuir*. **28**, 14323–9

53. Lehtio, J., Sugiyama, J., Gustavsson, M., Fransson, L., Linder, M., and Teeri, T. T. (2003) The binding specificity and affinity determinants of family 1 and family 3 cellulose binding modules. *Proc. Natl. Acad. Sci. U. S. A.* **100**, 484–489

54. Wada, M., Heux, L., and Sugiyama, J. (2004) Polymorphism of cellulose I family: Reinvestigation of cellulose IV_I. *Biomacromolecules*. **5**, 1385–1391

55. Kankare, J. (2002) Sauerbrey equation of quartz crystal microbalance in liquid medium. *Langmuir*. **18**, 7092–7094

56. Hu, F., Zhang, Y., Wang, P., Wu, S., Jin, Y., and Song, J. (2018) Comparison of the interactions between fungal cellulases from different origins and cellulose nanocrystal substrates with different polymorphs. *Cellulose*. **25**, 1185–1195

57. Benziman, M., Haigler, C. H., Brown, R. M., White, A. R., and Cooper, K. M. (1980) Cellulose

biogenesis: Polymerization and crystallization are coupled processes in Acetobacter xylinum. *Proc. Natl. Acad. Sci.* **77**, 6678–6682

58. Wood, P. J. (1980) Specificity in the interaction of direct dyes with polysaccharides. *Carbohydr. Res.* **85**, 271–287

59. Herth, W., and Schnepf, E. (1980) The fluorochrome, calcofluor white, binds oriented to structural polysaccharide fibrils. *Protoplasma*. **105**, 129–133

60. Scatchard, G. (1949) The attractions of proteins for small molecules and ions. *Ann. N. Y. Acad. Sci.* **51**, 660–672

61. Alekozai, E. M., GhattyVenkataKrishna, P. K., Uberbacher, E. C., Crowley, M. F., Smith, J. C., and Cheng, X. (2014) Simulation analysis of the cellulase Cel7A carbohydrate binding module on the surface of the cellulose I_{β}. *Cellulose*. **21**, 951–971
